# Data of history: An open-source and multiformat wall image dataset of Panam city, a historical place

**DOI:** 10.1016/j.dib.2024.110774

**Published:** 2024-07-31

**Authors:** Md Taimur Ahad, Yousuf Rayhan Emon, Sumaya Mustofa

**Affiliations:** Daffodil International University, Daffodil Smart City (DSC), Birulia, Savar, Dhaka 1216, Bangladesh

**Keywords:** Heritage preservation, Architectural heritage, Augmented reality, Virtual reality, Wall images

## Abstract

Historical data on monuments offers valuable insights into that period's past sculpture, architecture, and preferences. Realising the importance of historical data and the scarcity of data on historical places, this study presents a dataset collected from Panam City. Panam City, established in the late 1300s century, was the capital of the fifteenth-century Bengal ruler Isa Khan. The city was once an important trading and political centre and is now considered a world heritage site by the United Nations Educational Scientific and Cultural Organisation (UNESCO). Panam City is located in Sonargaon, Dhaka, Bangladesh. The aim of data collection is to capture past architectural design, materials used for the building, and the current state of the walls and structures of Panam City. This dataset can benefit researchers, architects, archaeologists, and cultural organisations. Historians and architects can gain insights into the wall's construction methods and materials, informing future restoration efforts. Historic datasets can create exciting AR/VR experiences by digitizing and 3D modelling historical artefacts and environments, integrating them into AR/VR platforms using game engines and development tools, and enhancing the user experience with interactive storytelling and educational content. Tourism boards and cultural heritage organisations can leverage this resource to develop engaging experiences that highlight the rich history and significance of Panama City. By making this data accessible, this study contributes to understanding and appreciating Panam City's historical significance while promoting innovative approaches to heritage preservation in the digital age. This dataset contains 2292 images of degraded wall classes such as Artistic, Corroded Brick, Corroded Plaster, Fungus, and Living Plant.

Specifications Table

**Table:** 

Subject	Artificial Intelligence, Computer Science Applications, Computer Vision and Pattern Recognition, Architecture.
Specific subject area	Deep learning-based image detection and classification of historical place's wall image types and features.
Data format	Raw: JPEG Annotation: TXT Annotation: XML
Type of data	Image
Data collection	The dataset is about the collection of pictures of Historic places. It includes images of degraded walls like Artistic, Corroded Brick, Corroded Plastic, Fungus, and Living Plant. This dataset was collected in Sonargaon, a place in the Narayanganj district, Dhaka Division of Bangladesh. The exact location of this place is 23.6421599°N and 90.6023361°E. These pictures were taken over two days, from October 6 to October 7, 2023. An iPhone was used to take these photos. The images were saved as JPEG files. JPEG is appropriate for these kinds of images as it can make the file size smaller without losing much information about the image. The dimensions of the images were chosen to be 3024×4032 pixels, then converted into 1080×1440 pixels. The resolution was originally 72 dpi. An essential part of this study was understanding the different classes of the degraded walls. An archaeologist helped with this part and classified the destroyed walls into five classes. Consisting of five degrading wall phases, the dataset has 2292 images. A subset of this dataset was manually annotated so segmentation-based experiments could be conducted efficiently using algorithms such as YOLO (You Only Look Once) and SSD (Single Shot Detection). These annotations were converted into TXT and XML formats using the Makesenseai web-based annotation tool.
Data source location	City: Panam City, Sonargaon. Country: Bangladesh Latitude and Longitude for collected data: 23.6421599°N and 90.6023361°E
Data accessibility	Repository name: Mendeley Data Data identification number: 10.17632/6h6rhwfkpy.1 Direct URL to data: https://data.mendeley.com/datasets/6h6rhwfkpy/1 The Panam Historic Place dataset is accessible through a user-friendly web interface, allowing users to browse, search easily, and download images and associated metadata. The interface is intuitive and requires no specialised knowledge, making it accessible to non-specialists. Users can download the dataset from .jpeg, .txt, and XML. The design of the Panam Historic Place dataset ensures that it is user-friendly for non-specialists. The web interface's simplicity, comprehensive documentation, and support resources ensure that users without a background in machine learning or VR/AR development can still effectively utilise the dataset. Including visualisation tools and metadata makes it easier for users to understand and analyse the images, promoting wider use and engagement with the dataset.

## 1 Value of the Data

• Researchers and organisations involved in preserving historical sites could use this dataset to analyse the degradation of walls and structures over time. It can assist in understanding the impact of various factors like weather, vegetation, and materials used on these structures, aiding in better preservation strategies.
• Utilizing this dataset, identifying patterns of decay and growth in historical structures is possible. This knowledge can contribute to creating more effective restoration methods and preventive conservation strategies, helping preserve cultural landmarks for future generations.
• Tourism boards, travel agencies, and cultural heritage organisations could utilise this dataset to create augmented reality (AR) or virtual reality (VR) experiences. These experiences could showcase the historical significance of different walls and structures within Panam City, enhancing tourist experiences and promoting cultural heritage.
• For machine learning and computer vision, researchers can use it to develop and train models that classify and analyse different wall types, specialise in identifying material types and degradation patterns, and enhance the capabilities of AI in cultural heritage preservation, contributing to advancements in image classification and object recognition techniques.
• As an educational resource, historical places datasets are essential for studies like environmental epidemiology, urbanisation, and landscape ecology, as they hold detailed information [1]. It offers visual support for their theoretical learning and fosters a deeper appreciation of cultural heritage.
• The dataset is accessible 2. **Technical Specification and Accessibility:** While the manuscript outlines the general use cases of the dataset, a more detailed technical description regarding the accessibility and usability of the dataset for different stakeholders would be beneficial. How user-friendly is the dataset for non-specialists in machine learning or VR/AR developers? Are there any tools or interfaces provided to facilitate its use?

## 2 Background

The dataset focused on *Panam City*, a UNESCO World Heritage Site in *South Asia*. This dataset aims to provide a detailed snapshot of *Panam City's* current state, offering high-resolution data on architectural features, urban textures, and vegetation. Its primary function is to be a reference point for future urban planning, historic preservation, and environmental analysis research. Compiling a historical place's image dataset for managing large metropolitan areas with inaccessible walls, identifying effective design solutions, and aiding in built heritage conservation decisions [2]. This dataset is valuable to architects, data scientists, and conservationists and encourages shared methodologies in cultural heritage study and preservation. Historical architectural data helps historians analyse and conserve historical objects, structures, and environments [3]. This dataset aligns with the ongoing efforts to protect digital assets for future use and research. Moreover, addressing the sustainability challenges in digital preservation, the dataset offers a structured, quantitative analysis of heritage sites [4]. Additionally, it highlights the potential of digital technologies to document and analyse sites of significant cultural value.

## 3 Data Description

The dataset presents a collection of historical wall images from Panam City, a UNESCO World Heritage Site in Sonargaon, Dhaka, Bangladesh. This Dataset includes 2292 images, classified into five classes: *Artistic, Corroded Brick, Corroded Plastic, Fungus, and Living Plant* [5]. Researchers involved in studying historical places in various fields and architects and organisations working on preserving historical sites could use this dataset. The leading directory labelled “Historic_Place_Dataset” is categorised into three subdirectories. Each serves a unique purpose:

*Pixel Modified Images:* The raw images are captured into 3024 * 4032 pixels with maximum details. Then, the images are modified into 1024 * 1440 pixels suitable for machine learning model training, balancing computational efficiency with sufficient information for the machine learning analysis task. The images are classified into five classes.

*Annotations:* Annotation is needed in machine learning and deep learning applications due to precise detection of the aimed object, where the rarest and most valuable samples represent few elements among thousands of annotated objects [6]. The annotated dataset is available in text and XML format in these sections.

**Table 1:** Brief description of the collected data.

No	Particulars	Descriptions
1	Walls	Historical Places
2	Numbers of features	5
3	Photo time	6–7 October 2023, Shooting All Day. The times are all daytime hours.
4	Geographical location	23.6421599°N and 90.6023361°E
5	Weather	Sunny Day
6	Temperature	26–28 °C

**Table 2:** Description of the dataset for wall images of historical places (Raw image).

Class	Artistic	Corroded Brick	Corroded Plaster	Fungus	Living Plant
No of Images	64	781	549	666	232

**Table 3:** Description of the dataset for wall images of historical places (Annotation).

Class	Artistic	Corroded Brick	Corroded Plastic	Fungus	Living Plant
No of Images	50	50	39	40	40

### 3.1 Description of the classes

The historic place dataset images were categorised into five classes: Artistic, Corroded Brick, Corroded Plaster, Fungus, and Living Plant. The selection of the specific wall features was carefully considered based on their significance in heritage conservation and architectural research. Each class represents a critical aspect of Panam City, Bangladesh's historic and architectural integrity. Here is a detailed justification for their inclusion:

#### 3.1.1 Artistic

**Fig. 1:**
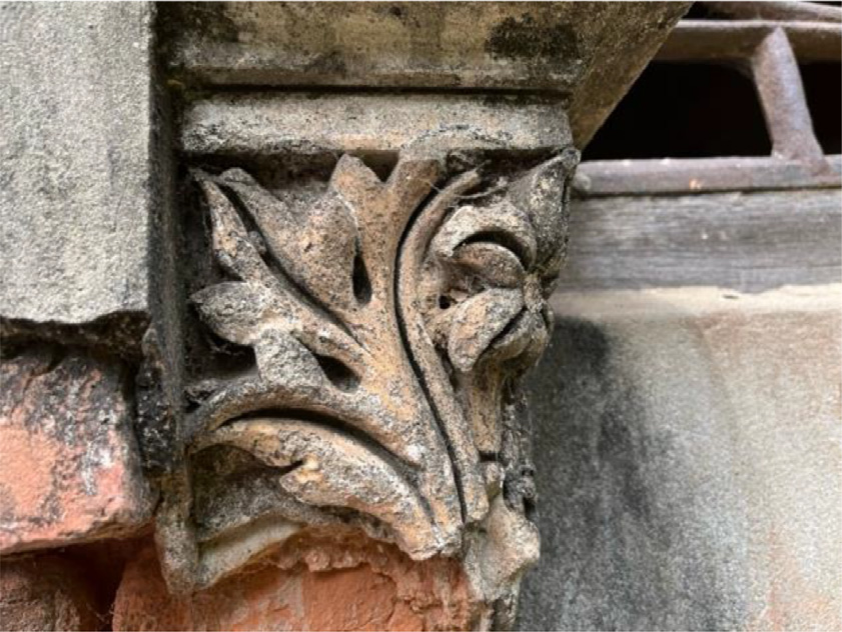
Artistic.

Artistic elements such as carvings, murals, and decorative motifs are integral to understanding the cultural and historical context of Panam City. That is the reason for collecting images of the artistic class. This class shows a close view of stone carving on an old wall. These features reflect the aesthetic and artistic values of the period in which they were created. The artistic images are typical of historical places and represent the detailed and creative work found in architecture from the past. The design includes leaf shapes, which are common in classic architecture, and despite being aged and weathered, they exemplify the high level of skill in traditional stone craftsmanship. Such features are essential for maintaining cultural identity and continuity [7] (See Fig. 1).

#### 3.1.2 Corroded brick

**Fig. 2:**
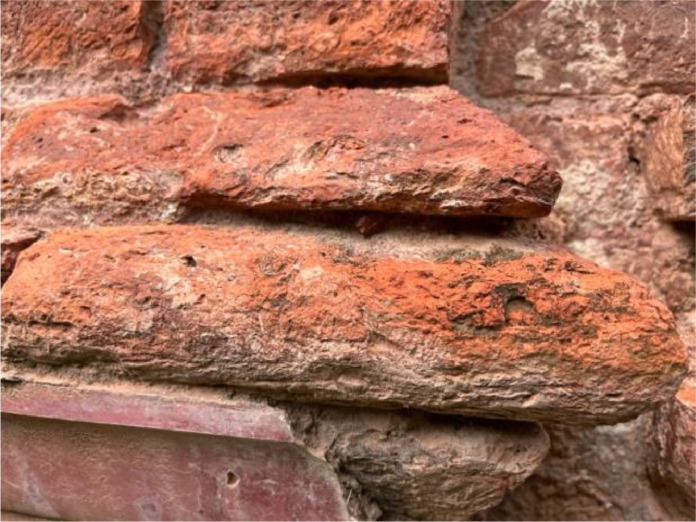
Corroded brick.

Corroded bricks are a common issue in historic buildings, impacting their structural integrity. Documenting areas with corroded bricks helps assess the extent of damage and plan restoration efforts [8]. This class has been included to study the worn down walls that have been damaged over the years because of long-term exposure to weather like rain, wind, and possibly pollution and the different chemical reasons which need to be exposed to the architects to plan an efficient reserving process of this historical places. (See Fig. 2).

#### 3.1.3 Corroded plaster

**Fig. 3:**
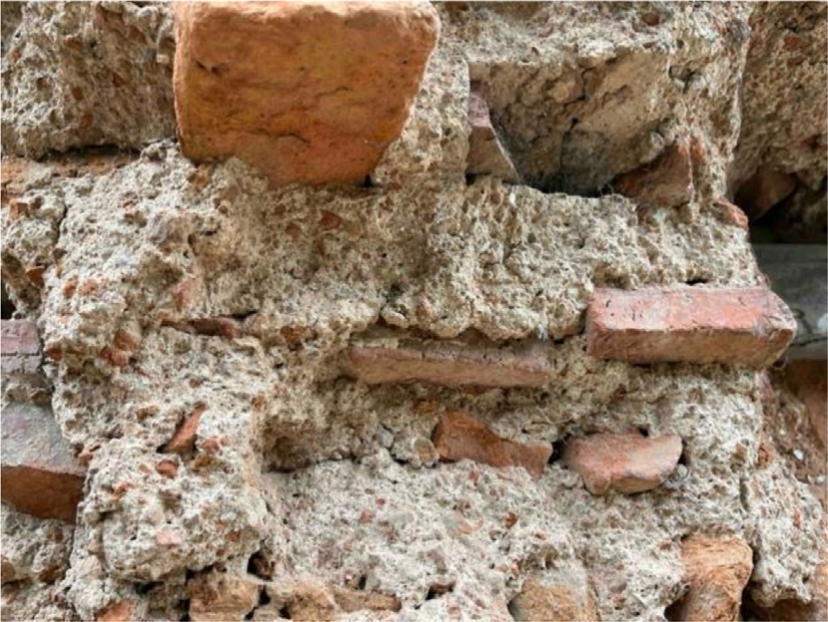
Corroded plaster.

Plaster often covers brick or stone walls, serving protective and decorative functions. Corroded plaster indicates underlying issues such as moisture ingress or material degradation [9]. It is a topic for studying how the ancient architects built the walls and the materials they utilised. Analysis of the images of this class will explain why these architectural structures have stood the test of time despite earthquakes, storms, and other natural disasters. This knowledge will be combined with modern knowledge to help design more robust architectures (See Fig. 3).

#### 3.1.4 Fungus

**Fig. 4:**
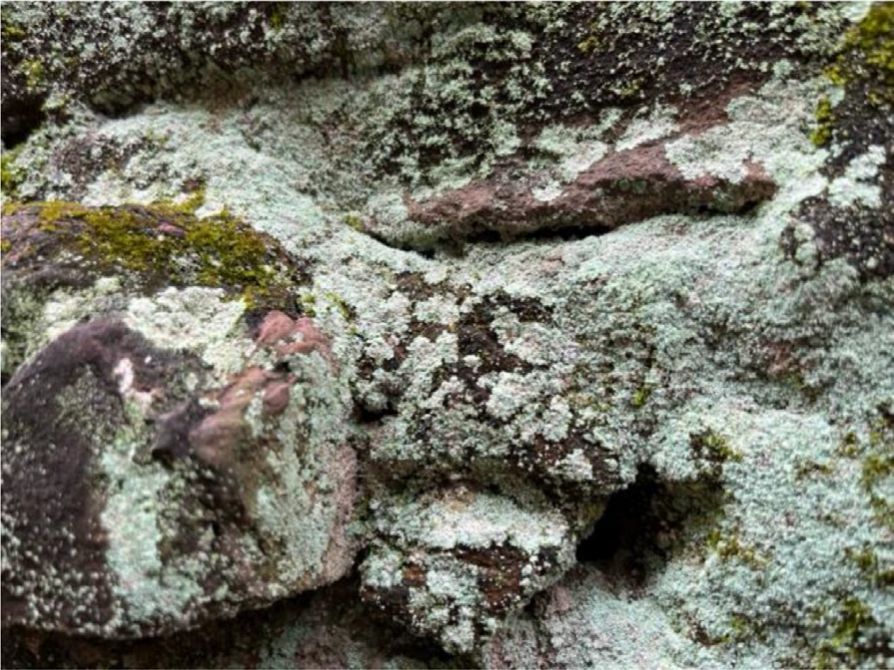
Fungus.

The fungus on walls indicates high humidity and poor ventilation, which are common problems in historic buildings. The fungus can accelerate organic materials' decay and compromise the building's structural integrity [10]. This class will reveal the presence of biological growth on the stone surface, characterised by fungal colonies. The growth patterns of these organisms can be quite telling of the micro-environmental conditions on and around the historical walls, offering biological insights and the process for the stone's conservation (See Fig. 4).

#### 3.1.5 Living plant

**Fig. 5:**
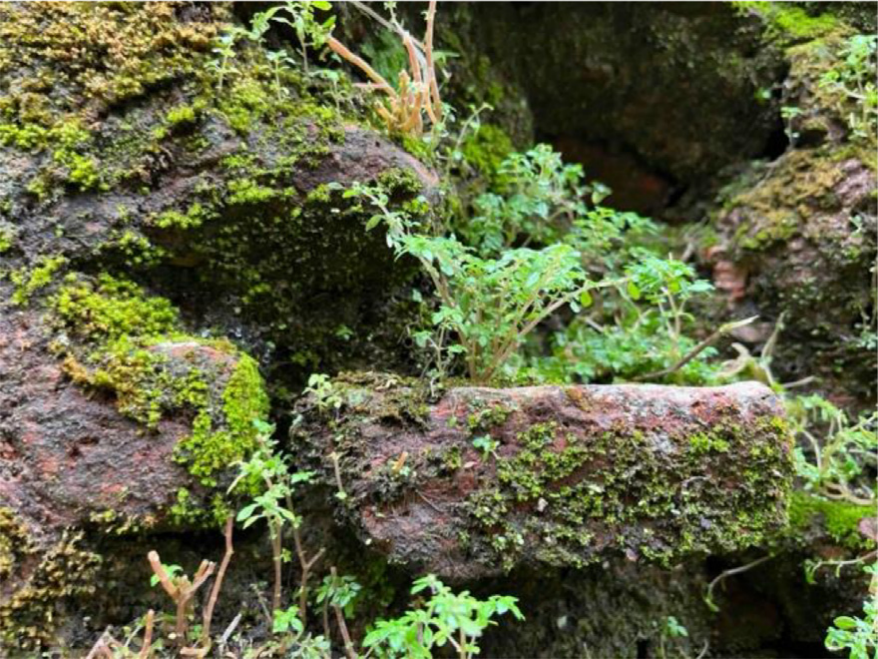
Living plant.

Plants growing on or near historic walls can cause physical damage through root growth and moisture retention. They can displace bricks or stones and introduce moisture, leading to further deterioration [11]. This class captures the interaction between flora and the built environment, demonstrating how nature can reclaim human-made structures and the potential impact of such biological growth on the integrity of historical constructions (See Fig. 5).

### 3.2 Significance of the dataset

The importance of historic data for Panam City, Bangladesh, is multi-faceted, encompassing cultural preservation, tourism, education, and research. The historical dataset of images is a critical tool for preserving and managing Panam Cityʼs rich cultural heritage. High-resolution photographs, 3D scans, and drone imagery allow for detailed documentation of its historic sites, ensuring accurate records for future generations. Prior research studies highlighted that data related to Bangladesh for artificial intelligence, machine learning and deep learning is scarce [[12], [13], [14], [15], [16], [17], [18]]. From a Bangladeshi perspective, therefore, the dataset is significant.

Images of Panam Cityʼs historic sites play a pivotal role in promoting tourism, a significant economic driver for the region. High-quality visuals attract visitors and can be utilised in marketing campaigns to boost tourist numbers. According to a 2022 report by the World Tourism Organization, digital imagery and virtual tours have become essential in travel planning, significantly influencing tourists' destination choices. Educational institutions and researchers rely heavily on image data to study and teach the history, art, architecture, and archaeology of Panam City. Digital archives provide access to rare and significant artefacts and sites that might not be easily accessible. This has been incredibly beneficial during the COVID-19 pandemic, where travel restrictions made physical visits difficult, prompting a surge in virtual learning and remote research.

In the aftermath of natural disasters, accurate image data of Panam Cityʼs historic places is essential for assessing damage and planning recovery efforts. These images are crucial in community engagement and fostering a sense of identity. They help the local community connect with their past, celebrate their heritage, and educate younger generations. Local governments and organisations often use these images in exhibitions, public displays, and digital archives to promote cultural awareness and pride.

Recent technological advancements, such as augmented reality (AR) and virtual reality (VR), have revolutionised how we interact with historic sites. These technologies rely heavily on high-quality image data to create immersive experiences that can be accessed by people worldwide, regardless of their physical location. This democratises access to Panam City's cultural heritage and supports inclusive education.

**Table 4:** Brief description of the dataset.

No	Particulars	Description
1	Wall	Historical Place
2	Classes	5
3	Original Image	JPEG; 1080×1440 pixels:72 dpi
4	Annotated file format	TXT and XML
5	Original dataset size	Size of each image: 700–900 KB Folder size: 1.43 GB
6	Annotated dataset size (TXT)	Annotation folder size: 1.48 GB
7	Annotated dataset size (XML)	Annotation folder size: 166 KB
8	Dataset	Total folder size: 2.92 GB

**Table 5:** Brief description of the folders.

No	Folder Name	File Format in the folder	Description
1		zip	It is conveniently Packaged for download.
2		All format together	
3	Original Image	jpeg	Image file for detailed view
4	Annotations	TXT and XML	Annotated file with information such as category, location and size.

### 3.3 Description of the dataset folder

#### 3.3.1 Original images

The main folder contains all the images in the dataset, with a folder size of 2.92 GB. The images have varying sizes, with the highest 1000 KB and the lowest 590 KB. All images are in the 1080×1440 pixel format and have a resolution of 72 dpi. This folder is divided into five subfolders, each containing images of specific classes such as Artistic, Corroded Brick, Corroded Plastic, Fungus, and Living Plant. We arranged the images this way to make it easier for researchers to find images based on their classification needs. Each image was named by numbering, such as IMG_3369.jpeg, IMG_4881.jpeg, IMG_5034.jpeg, etc.

#### 3.3.2 Annotations

An open-source software, MakesenseAi, was used to annotate the image files. Annotation is the labelling of the region of interest of an image. The annotation format of an image allows the detection, classification, and grouping of images recognisable to machines through machine learning. Annotation files contain the bounding boxes in images for object detection tasks. In this format, each image in the dataset should have a corresponding text file with the same name as the image, containing the bounding box annotations for that image. The annotation folder contains 219 TXT files and 219 images. Each image was renamed according to its class, such as IMG_3369.jpeg and IMG_3369.txt. The other annotation folder contains 219 XML files as same.

## 4 Experimental Design, Materials and Methods

### 4.1 Theoretical knowledge gathering

An extensive literature review was conducted to gather theoretical knowledge to study historical wall images. This review focused on academic research papers, historical preservation websites, and specialised articles on Panam Cityʼs historical sites. This literature review aimed to identify key features and factors relevant to the historical wall images in the dataset. This source provides an understanding of Panam Cityʼs walls' relevant historical and cultural aspects. This approach provides the necessary theoretical background to support the research.

### 4.2 Site selection and data collection

After collecting theoretical knowledge about the walls of historical places, the next step is to select the appropriate class and time for collecting the data. The selection process included accessibility and sufficient classes to be studied. However, Panam City's wall from Sonargaon, Bangladesh, has been selected among several available Historical Places. From there, we collected a large amount of data and found five classes focusing on degraded walls.

### 4.3 Digital image acquisition

Accurate detection, identification, and classification require proper image acquisition. Taking clear and high-quality pictures is essential to avoid incorrect identification and classification. The accuracy of the models also depends on the clarity and precision of the photographs, as models cannot provide accurate results if the dataset is not precise enough. Therefore, this study specialises in this section during image collection to ensure the best possible results. Initially, the iPhone 13 was used to capture all the pictures. Captured images were the size of 3024×4032 pixels with a 72-dpi resolution. Initially, images were taken in jpeg format.

The section of the resolution of 72 dpi for images sized at 1080×1440 pixels was chosen to ensure manageable file sizes and broad accessibility, making the dataset usable by a wider audience, including those with limited technical resources. While this resolution may limit detailed architectural analyses, it remains sufficient for general research, educational purposes, and preliminary assessments. Higher-resolution images can be provided upon request for studies requiring finer details.

**Table 6:** Descriptions of camera devices.

No	Particulars	Device
1	Phone	iPhone
2	Phone Model	iPhone 13
3	Camera Manufacturer	Apple
4	Camera sensor	Sony sensor
5	Camera pixel	12 Megapixels
6	Aperture Value	Wide: ƒ/1.6
7	Exposure time	1/60 s
8	Camera flash	No
9	Focal length	5 mm

### 4.4 Feature classification

**Fig. 6:**
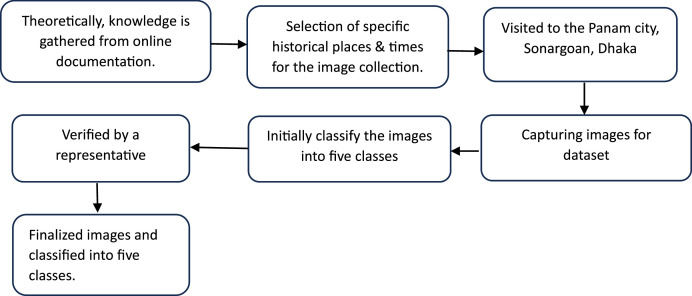
Process of image collection.

To ensure the accuracy of the classification model, images were collected based on their corresponding features (See Fig. 6). The dataset has five(5) classes: Artistic, Corroded Brick, Corroded Plastic, Fungus, and Living Plant. A representative from the archaeologist, an expert in this field, helped to identify the classes, and the gathered theoretical knowledge has been applied in the data collection process. The classification was carried out following a set of criteria:

### 4.5 Image processing

The images were resized by reducing their pixel count, making them more manageable for researchers. This process involves converting high-resolution images into lower-resolution ones and compressing their file size. A software tool called ‘Microsoft Powertoys’ was used to resize and enhance the images. The final image size was 640×480 pixels, with a resolution of 72 dpi. Additional details on image processing can be found in Section 2.3.

### 4.6 Image annotation

Image annotation is mandatory for specific deep learning models (such as YOLO, R-CNN, SSD, U-Net, and FCN). Considering the situation of the researchers interested in those specific models that use annotation, A part of the dataset was annotated. From each class, 115 images were taken for annotation. All the annotation files are in txt format.

## Limitations

Among the limitations, besides high-resolution images, resolution low-resolution images could be included. Data scientists could find important insight from two (2) version scientists could find important insight from two (2) versions of images. More images from other historical areas could be collected to understand the historical place dataset comprehensively. However, in the future, we aim to overcome such limitations.

In the future, we aim to collect more data and increase the number of classes. Thus, more annotated files will be created. Although potential applications of the dataset are discussed, a pilot study demonstrating the datasetʼs application in heritage conservation, machine learning model training, or VR/AR development will be undertaken in the future. The pilot project is expected to demonstrate the utilisation of the dataset.

Among other limitations, a few numbers of images were annotated in this dataset; however, in the future, a complete annotation of the dataset will be added to the next version. There are more formats, such as COCO, JSON, etc. This study further aims to add these popular and mainly used formats.

## Ethics Statement

Neither plants nor animals contracted an infection while the data were being gathered. The current work does not use human subjects, animal trials, or data gathered from social media sites. The authors strictly maintained the ethical code of data in brief during the dataset collection experiment.

## CRediT Author Statement

**Md Taimur Ahad:** Original Draft, Data Curation, Conceptualization, Supervision, Review & Editing; **Yousuf Rayhan Emon:** Investigation, Methodology, Writing - Original Draft, Visualization, Data Curation, Resources. **Sumaya Mustofa:** Data Curation, Conceptualization, Review & Editing.

## Data Availability

A multi-format open-source historic wall image dataset for architects, historians, data scientists to detect, classify, and analyze (Original data) (Mendeley Data).

## Appendix Supplementary materials

Image, application 1
