# Supplementary figures and images for: Data of history: An open-source and multiformat wall image dataset of Panam city, a historical place
Source: Data Brief. 2024 Jul 31;56:110774. doi: 10.1016/j.dib.2024.110774 (PMC11364048; doi:10.1016/j.dib.2024.110774)

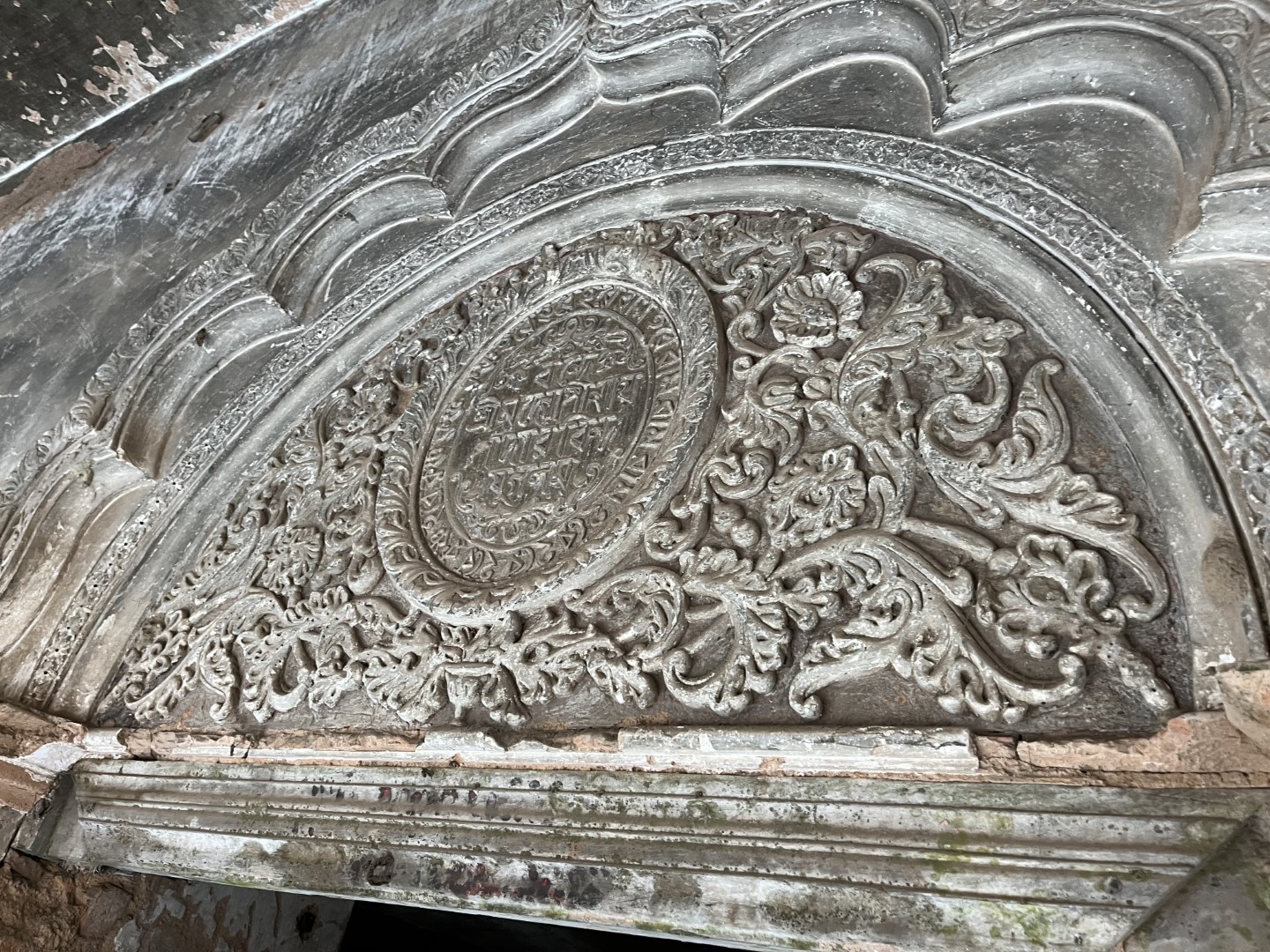

Supplement: Supplementary file 1 [file mmc1.zip › Demo_Historic_Place_Dataset/Artistic/IMG_3369.JPG]

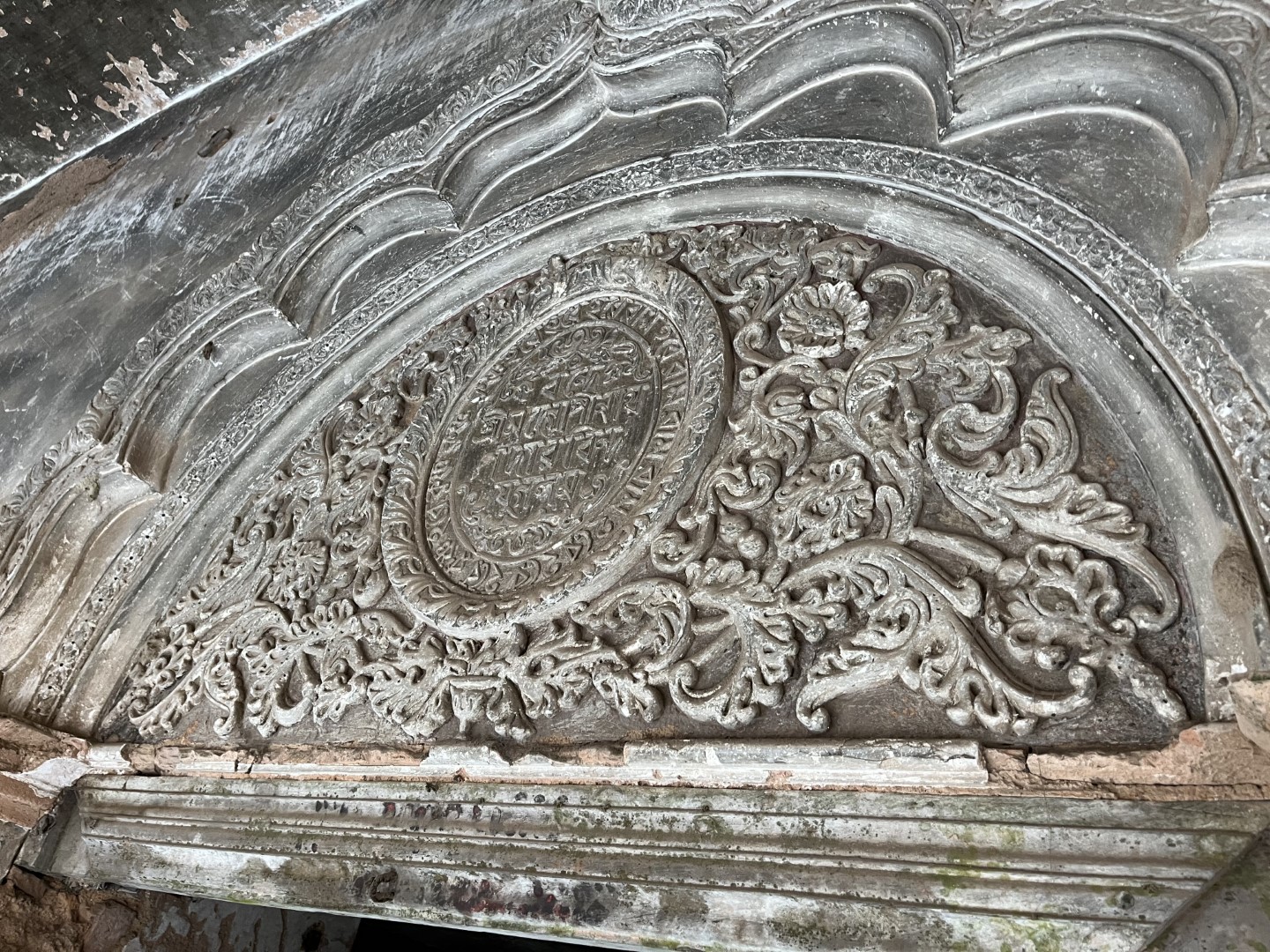

Supplement: Supplementary file 1 [file mmc1.zip › Demo_Historic_Place_Dataset/Artistic/IMG_3370.JPG]

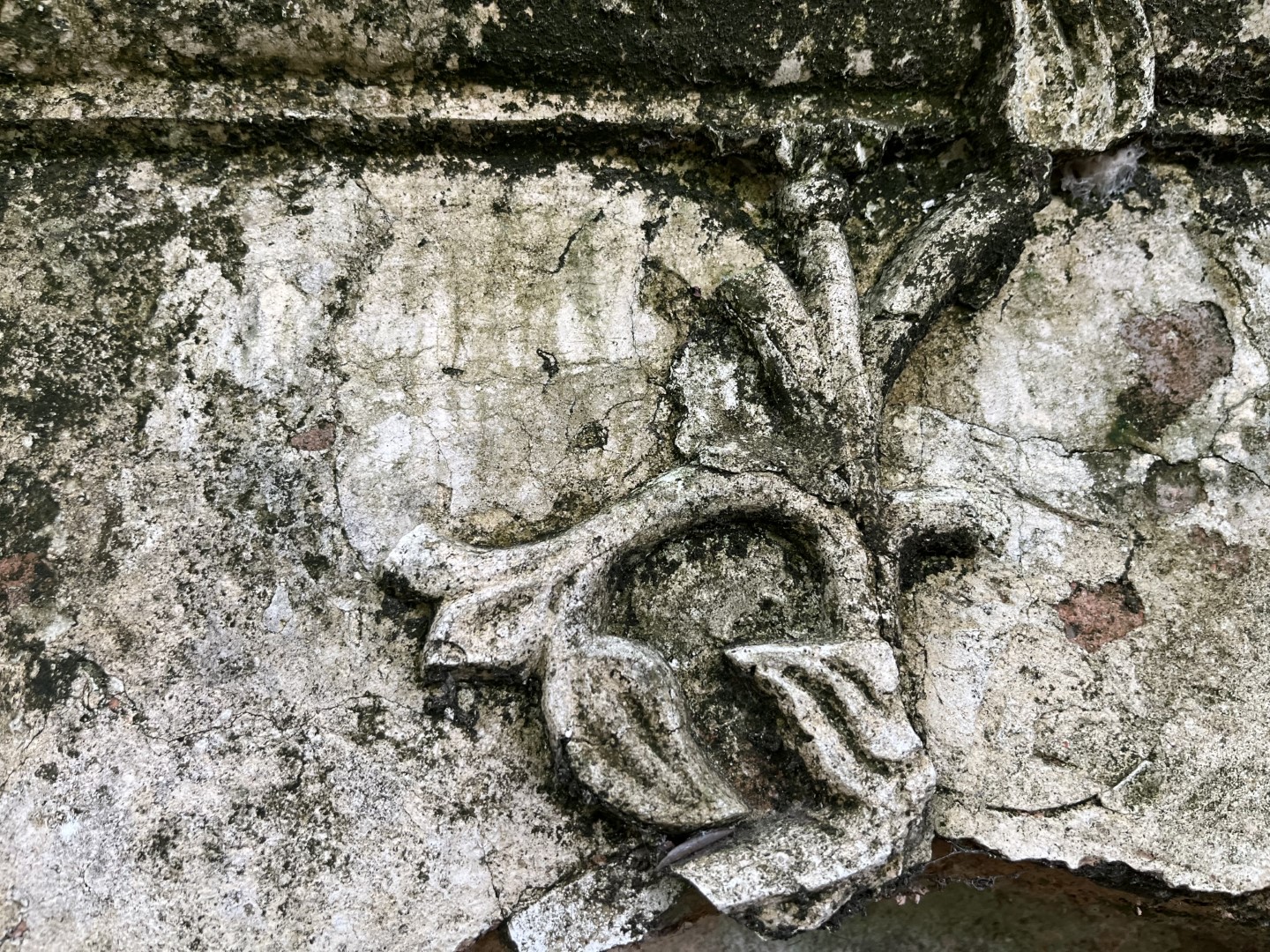

Supplement: Supplementary file 1 [file mmc1.zip › Demo_Historic_Place_Dataset/Artistic/IMG_3384.JPG]

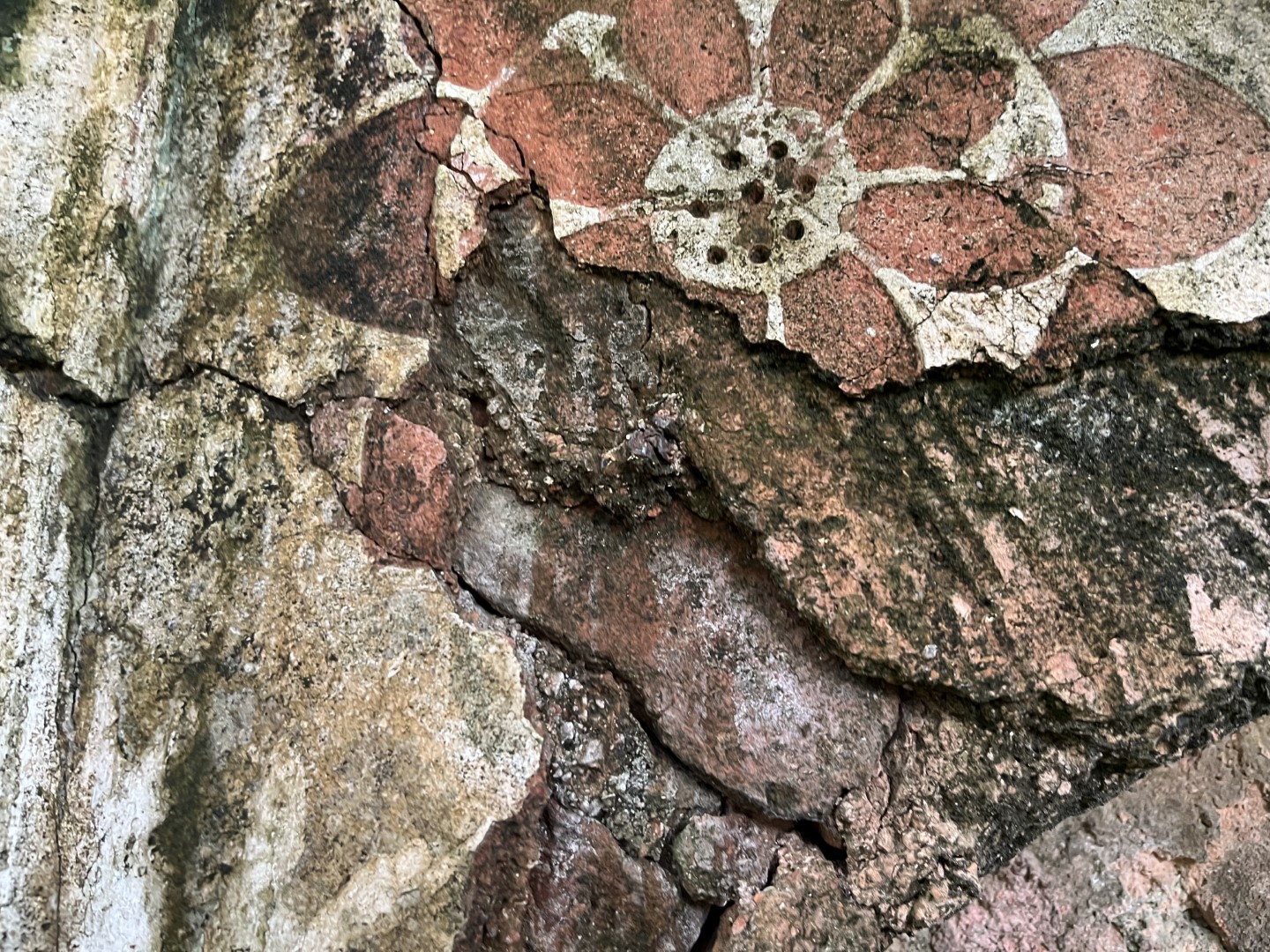

Supplement: Supplementary file 1 [file mmc1.zip › Demo_Historic_Place_Dataset/Artistic/IMG_3385.JPG]

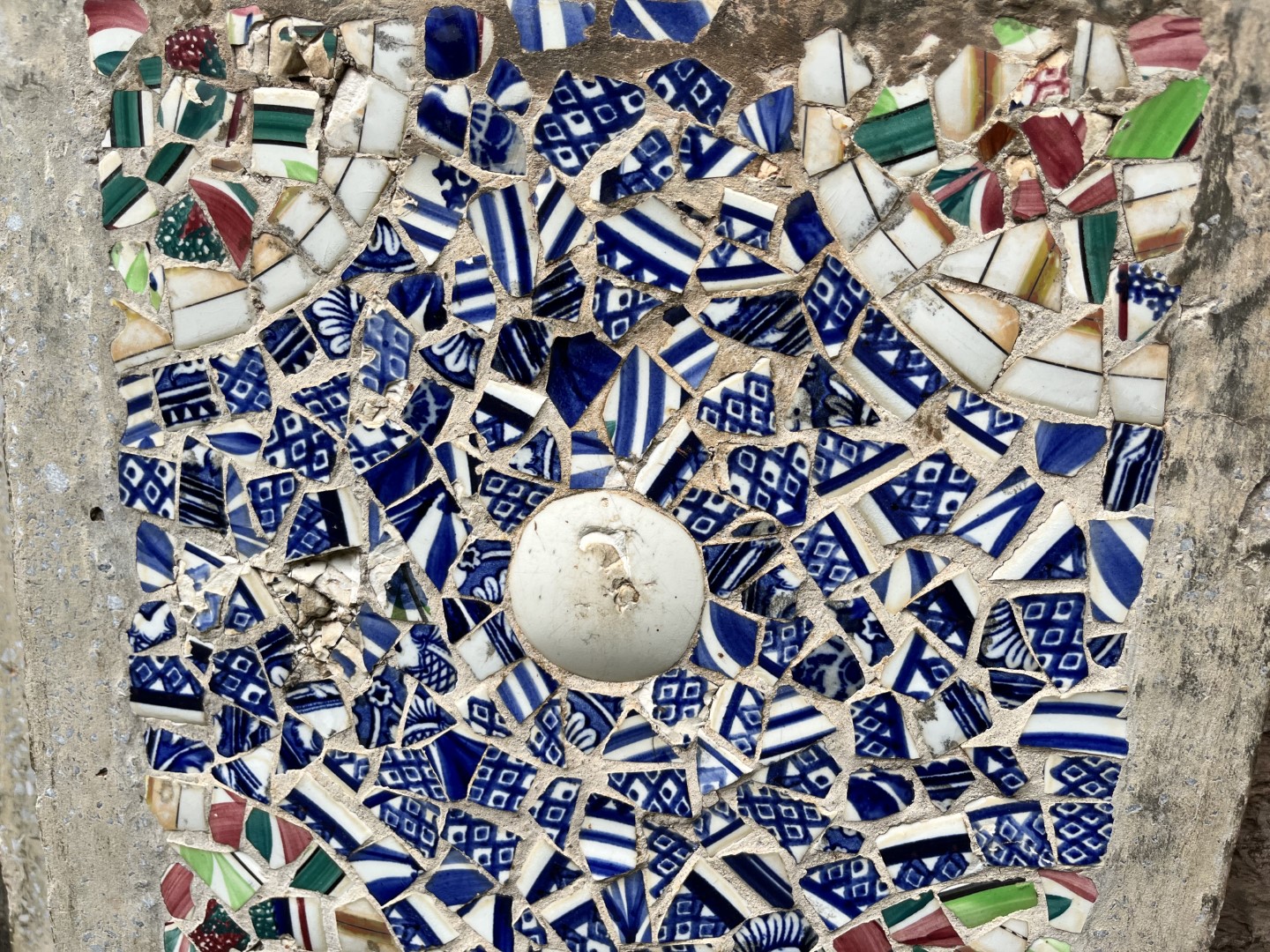

Supplement: Supplementary file 1 [file mmc1.zip › Demo_Historic_Place_Dataset/Artistic/IMG_3391.JPG]

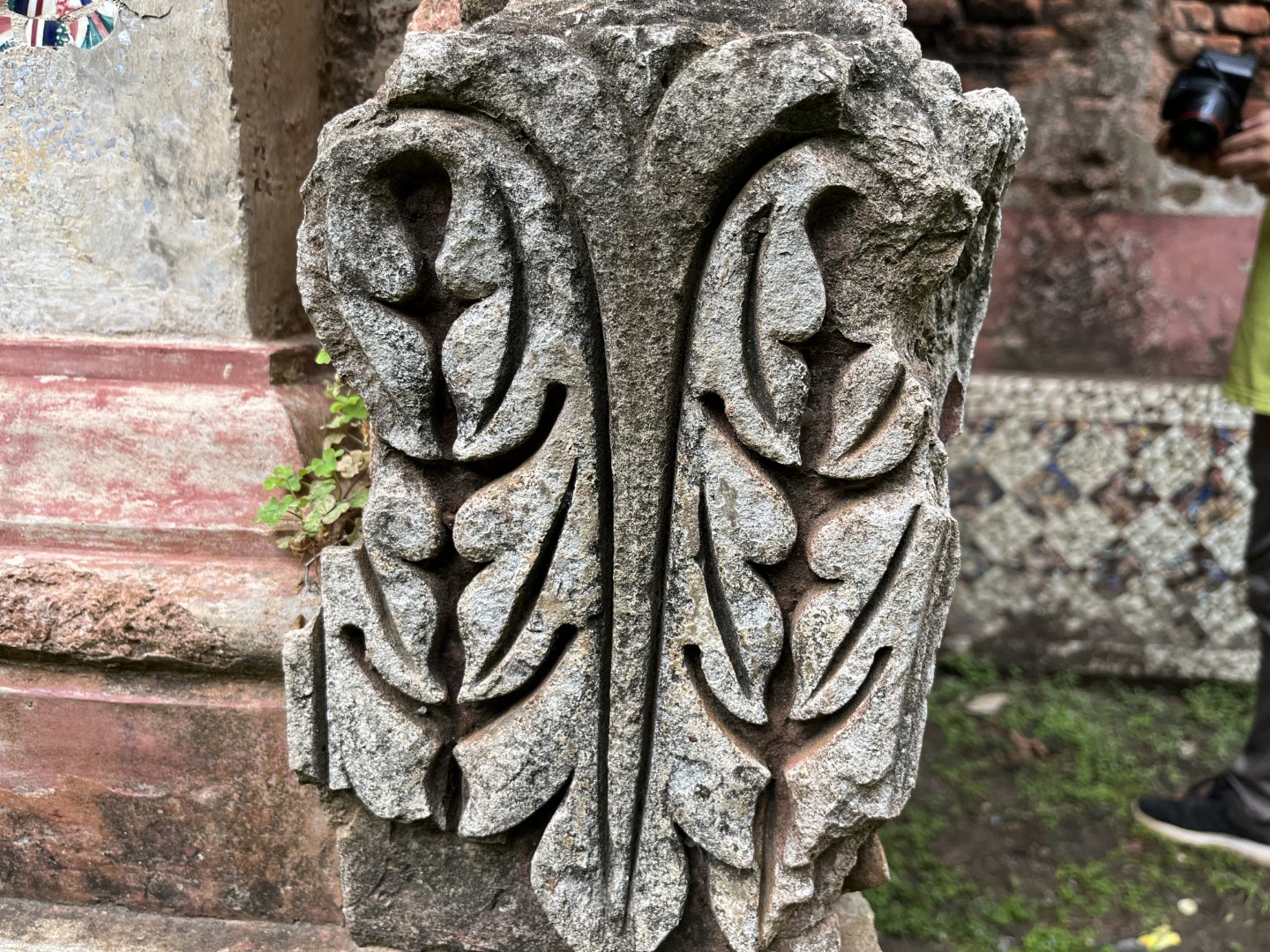

Supplement: Supplementary file 1 [file mmc1.zip › Demo_Historic_Place_Dataset/Artistic/IMG_3392.JPG]

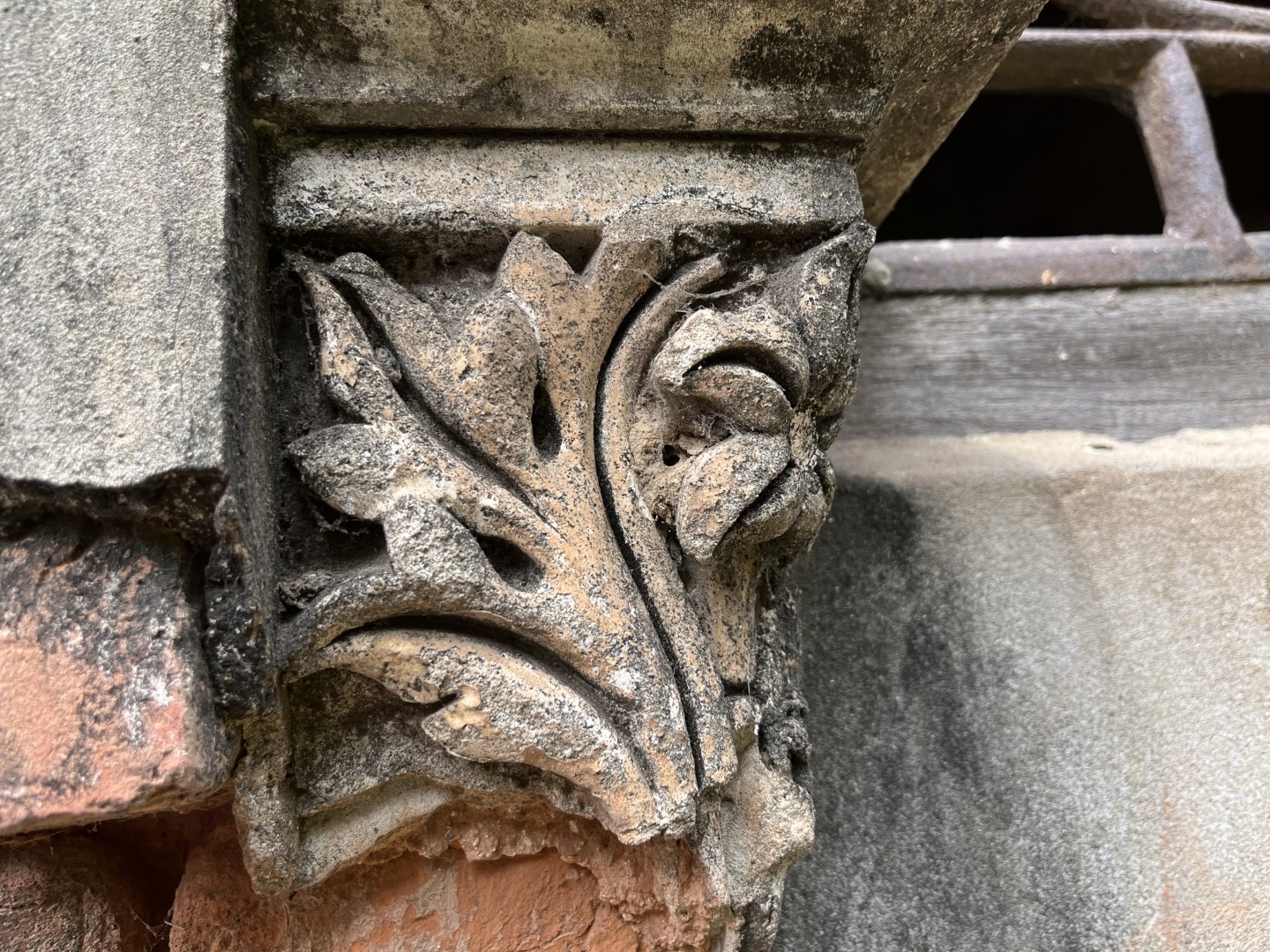

Supplement: Supplementary file 1 [file mmc1.zip › Demo_Historic_Place_Dataset/Artistic/IMG_3393.JPG]

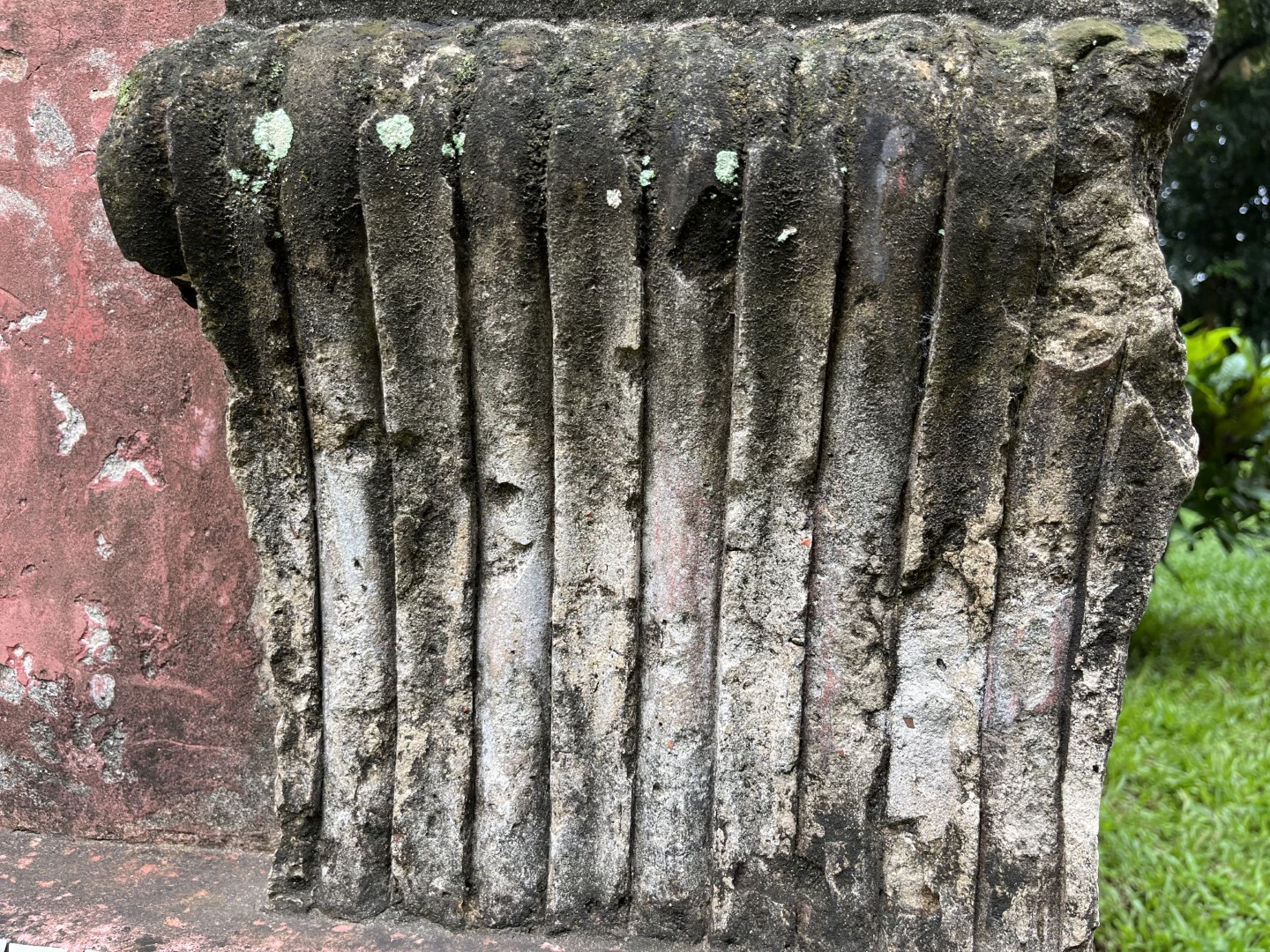

Supplement: Supplementary file 1 [file mmc1.zip › Demo_Historic_Place_Dataset/Artistic/IMG_3395.JPG]

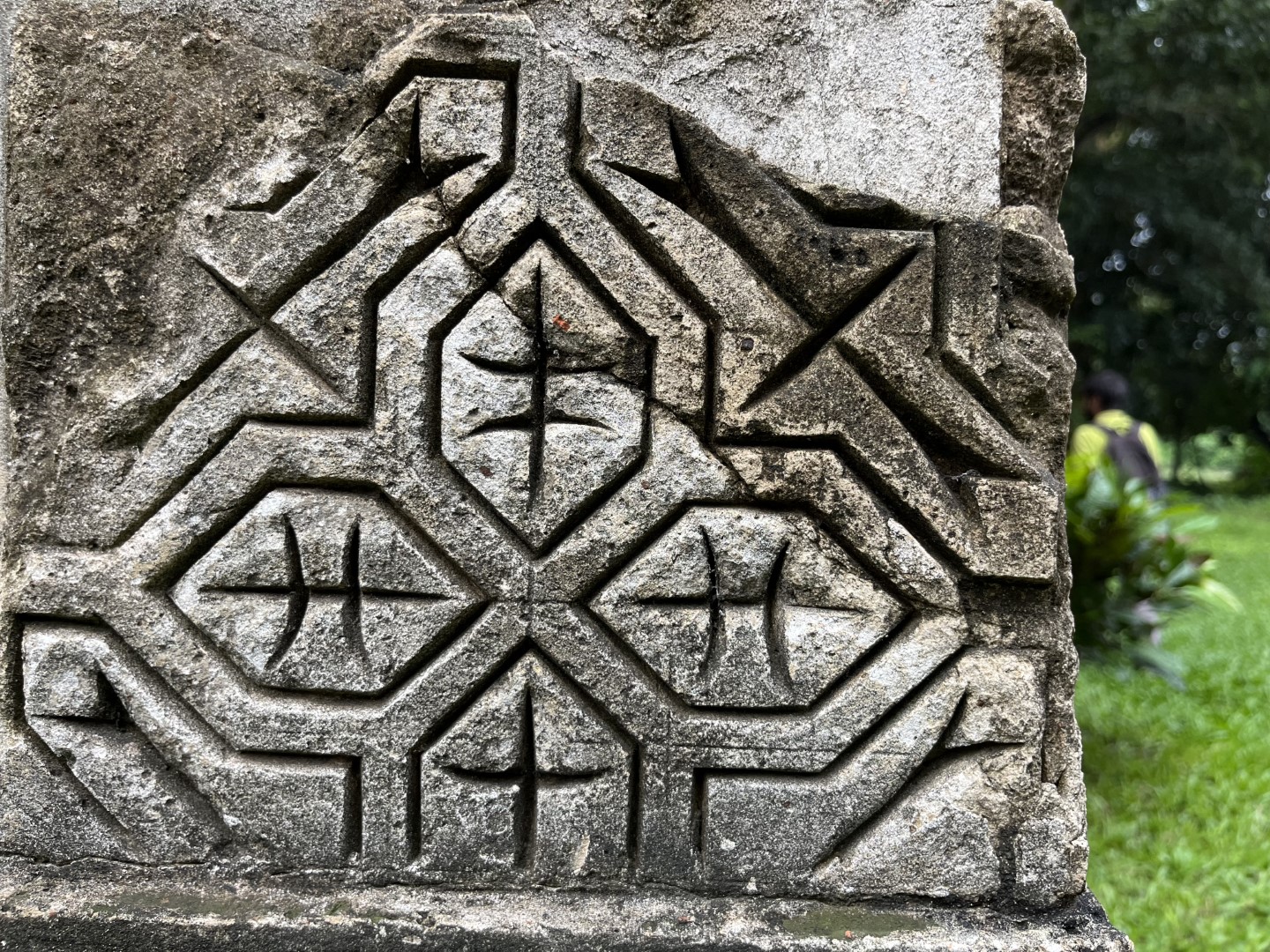

Supplement: Supplementary file 1 [file mmc1.zip › Demo_Historic_Place_Dataset/Artistic/IMG_3396.JPG]

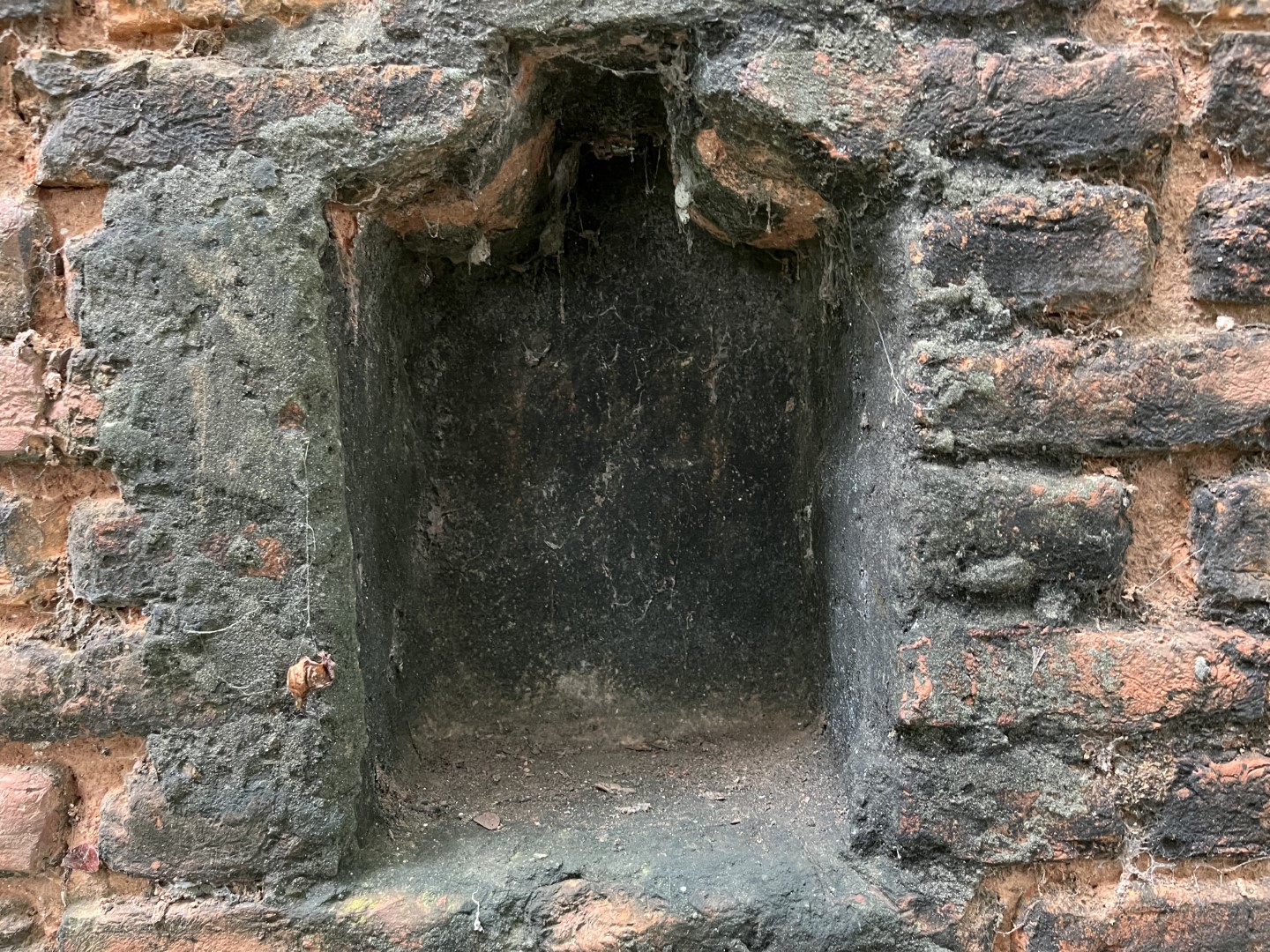

Supplement: Supplementary file 1 [file mmc1.zip › Demo_Historic_Place_Dataset/Artistic/IMG_3400.JPG]

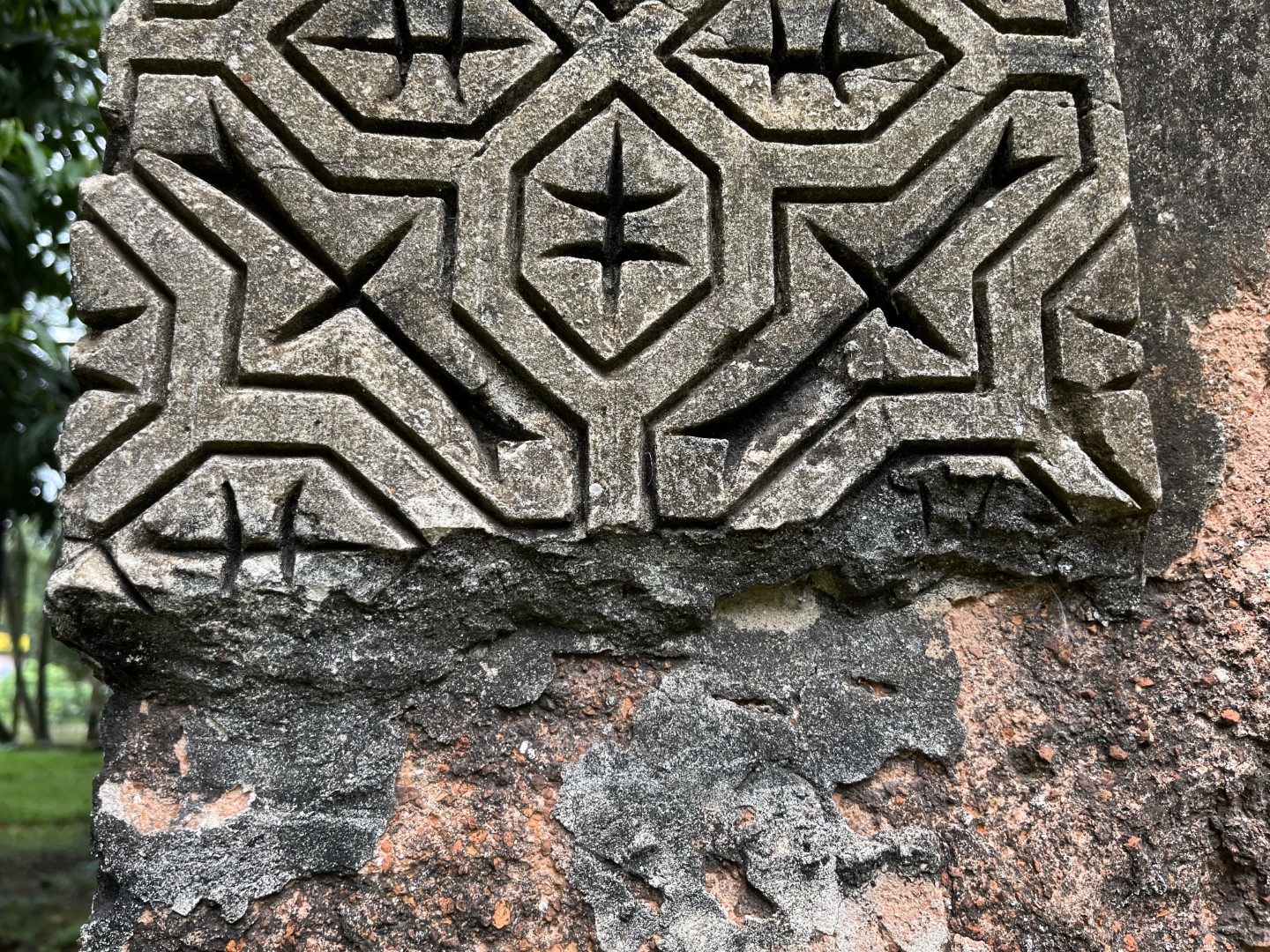

Supplement: Supplementary file 1 [file mmc1.zip › Demo_Historic_Place_Dataset/Artistic/IMG_3409.JPG]

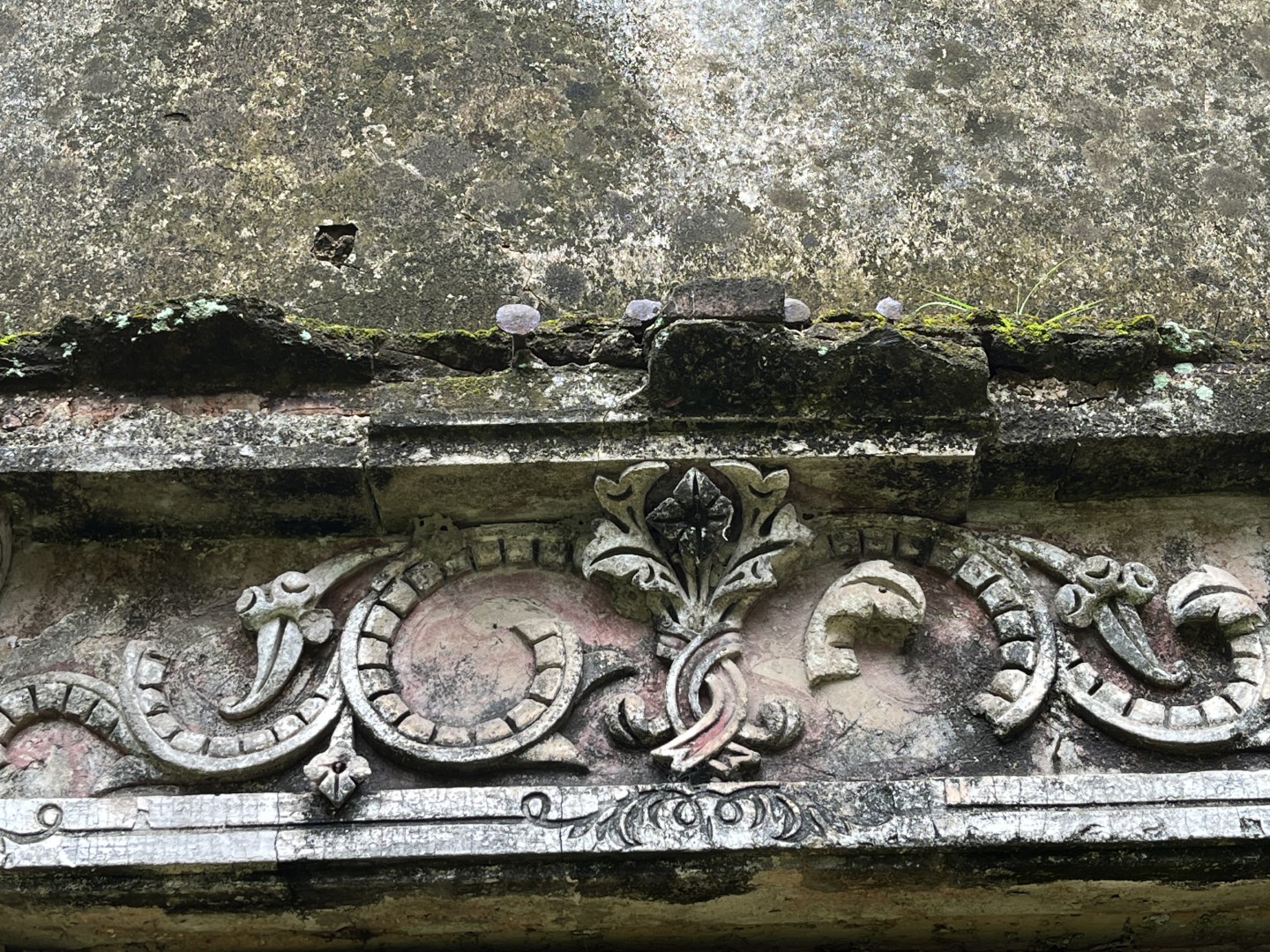

Supplement: Supplementary file 1 [file mmc1.zip › Demo_Historic_Place_Dataset/Artistic/IMG_3412.JPG]

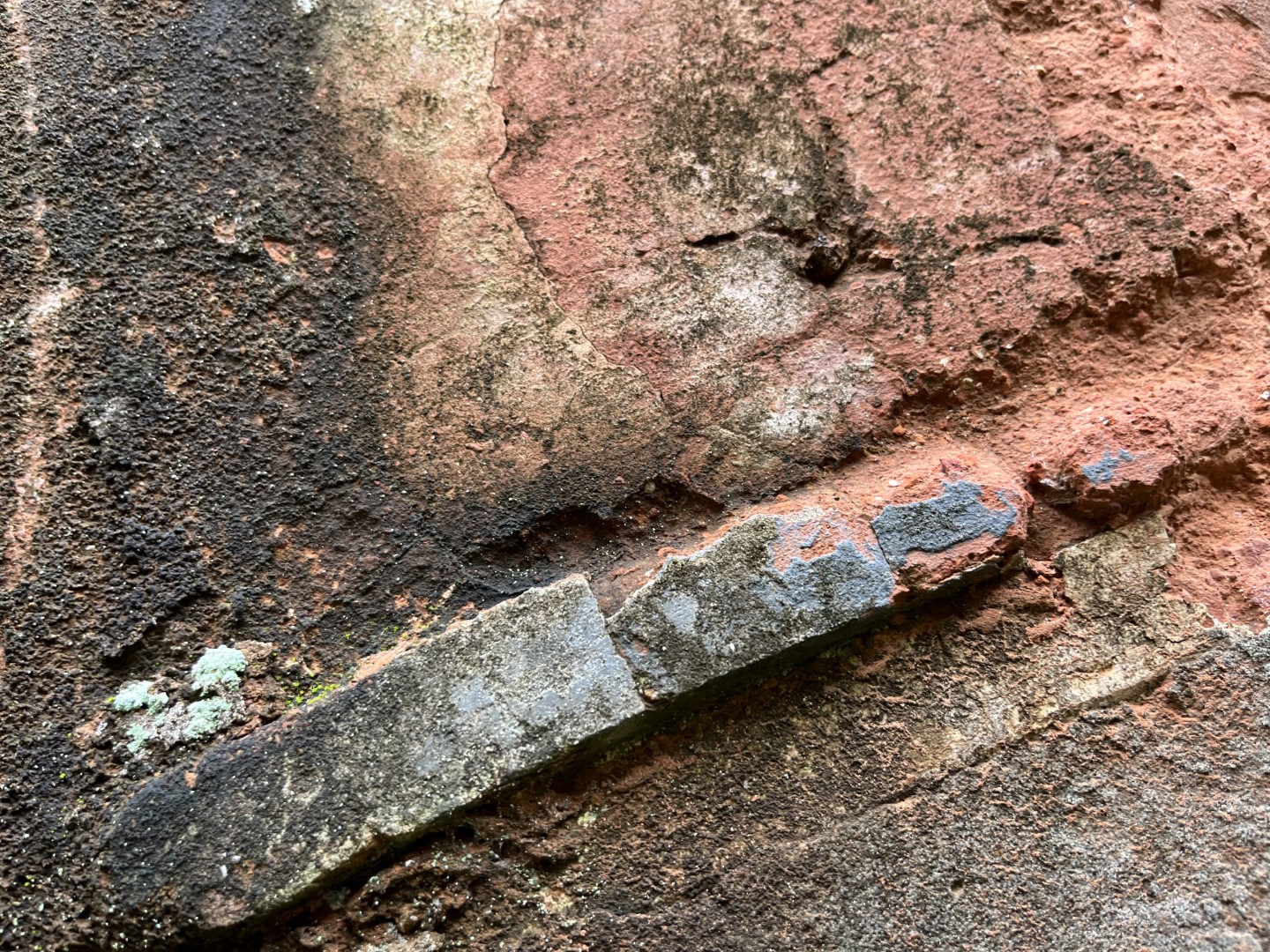

Supplement: Supplementary file 1 [file mmc1.zip › Demo_Historic_Place_Dataset/Artistic/IMG_3483.JPG]

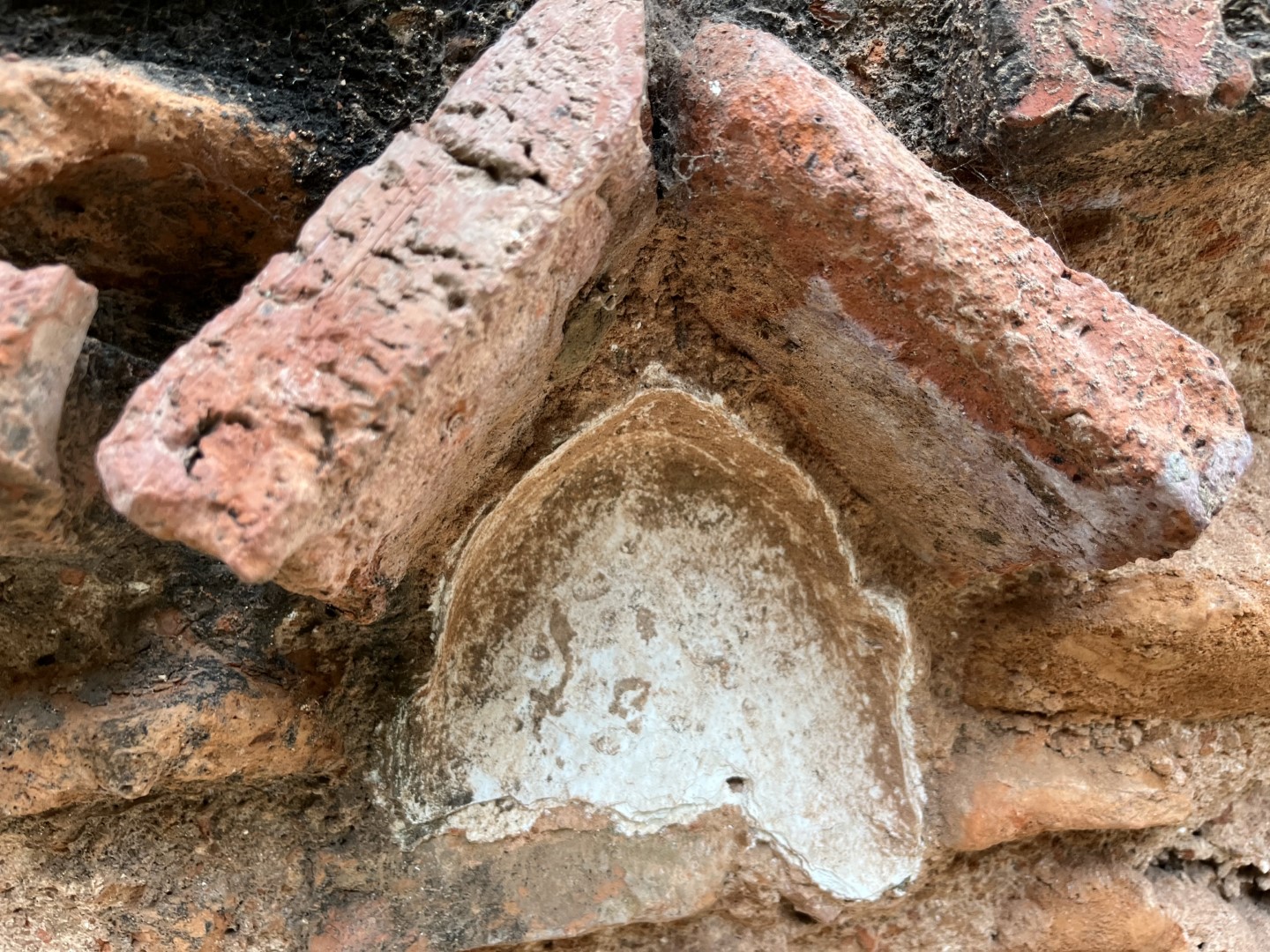

Supplement: Supplementary file 1 [file mmc1.zip › Demo_Historic_Place_Dataset/Artistic/IMG_3546.JPG]

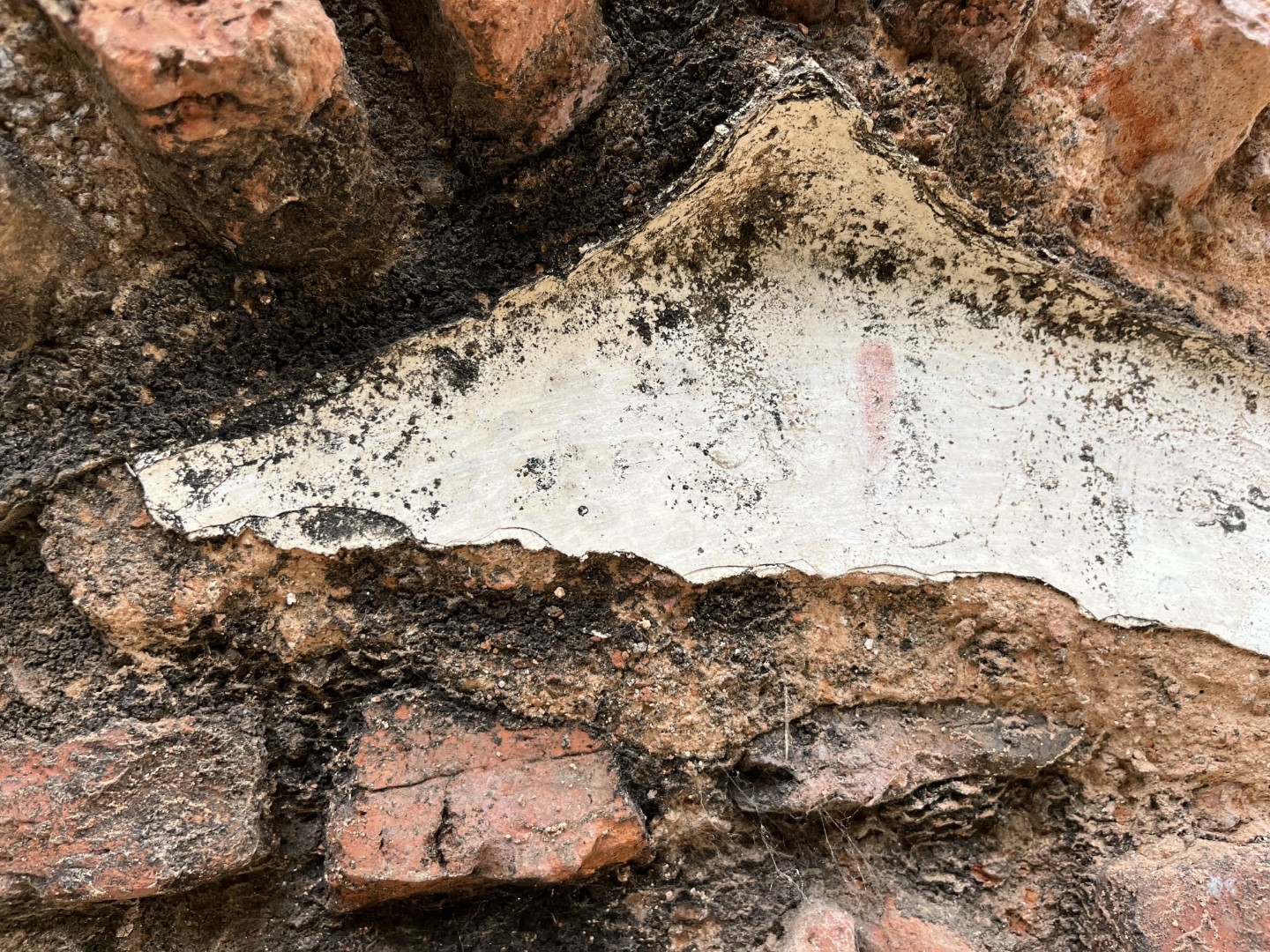

Supplement: Supplementary file 1 [file mmc1.zip › Demo_Historic_Place_Dataset/Artistic/IMG_3547.JPG]

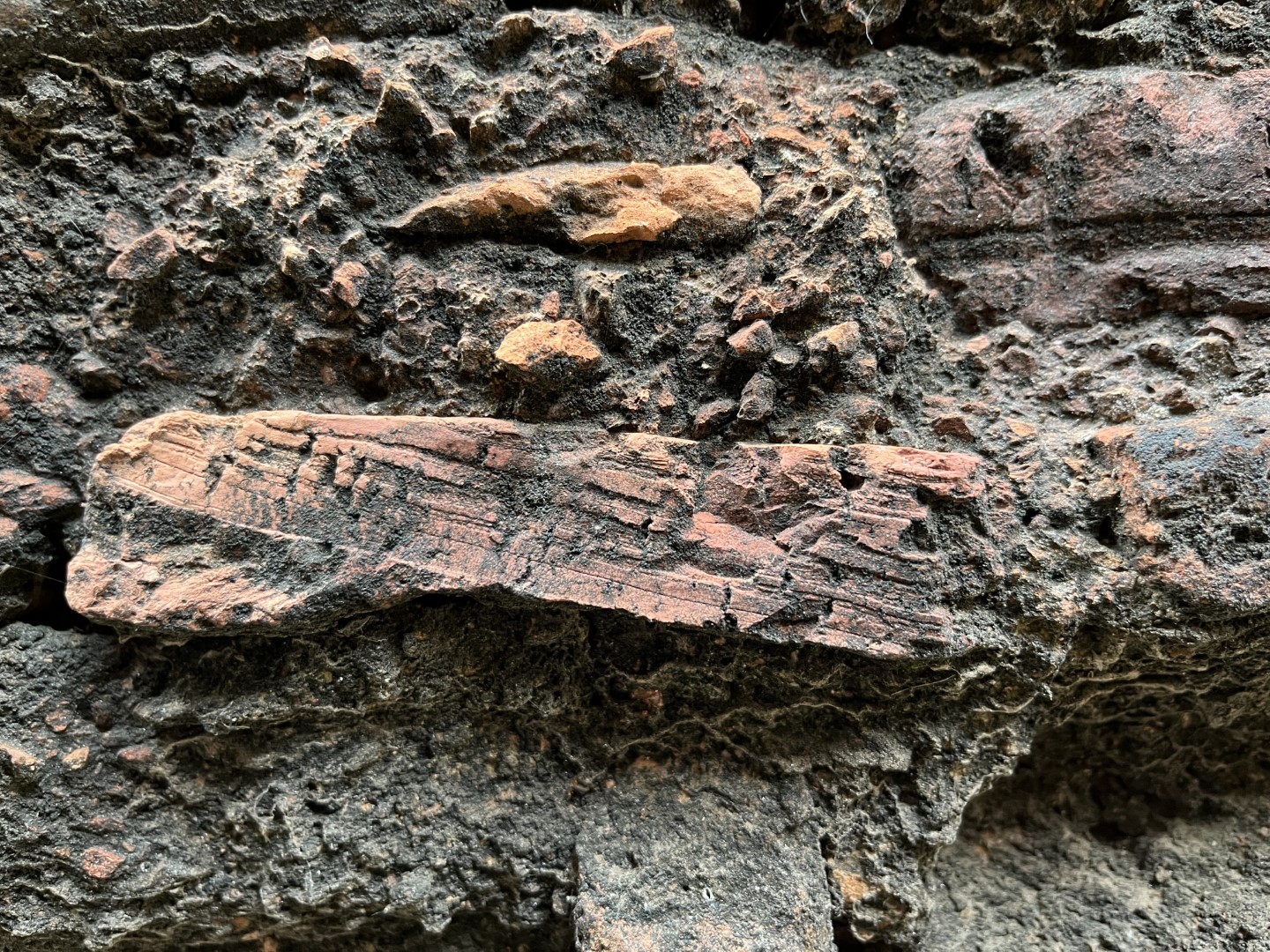

Supplement: Supplementary file 1 [file mmc1.zip › Demo_Historic_Place_Dataset/Corroded brick/IMG_3360.JPG]

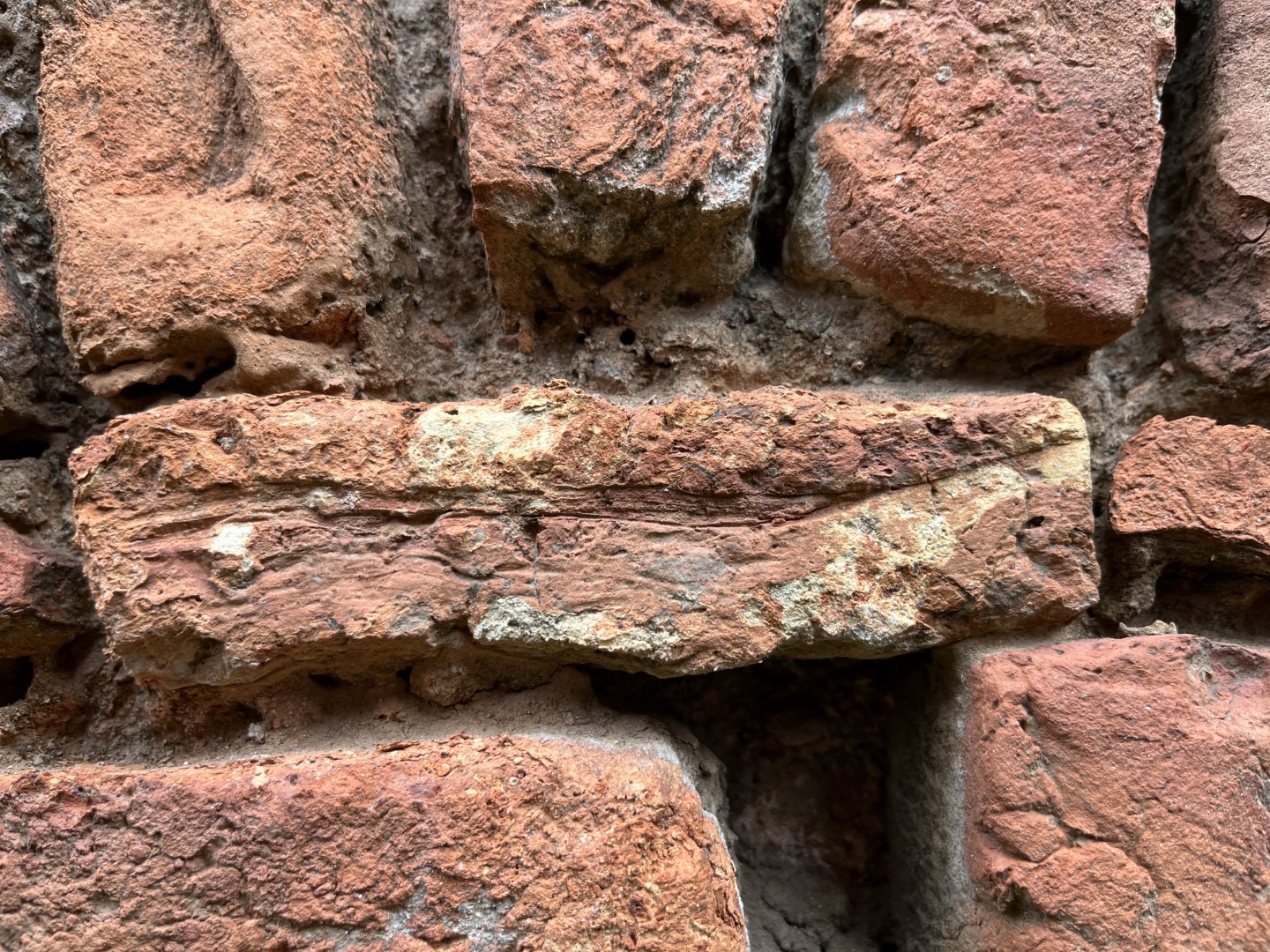

Supplement: Supplementary file 1 [file mmc1.zip › Demo_Historic_Place_Dataset/Corroded brick/IMG_3363.JPG]

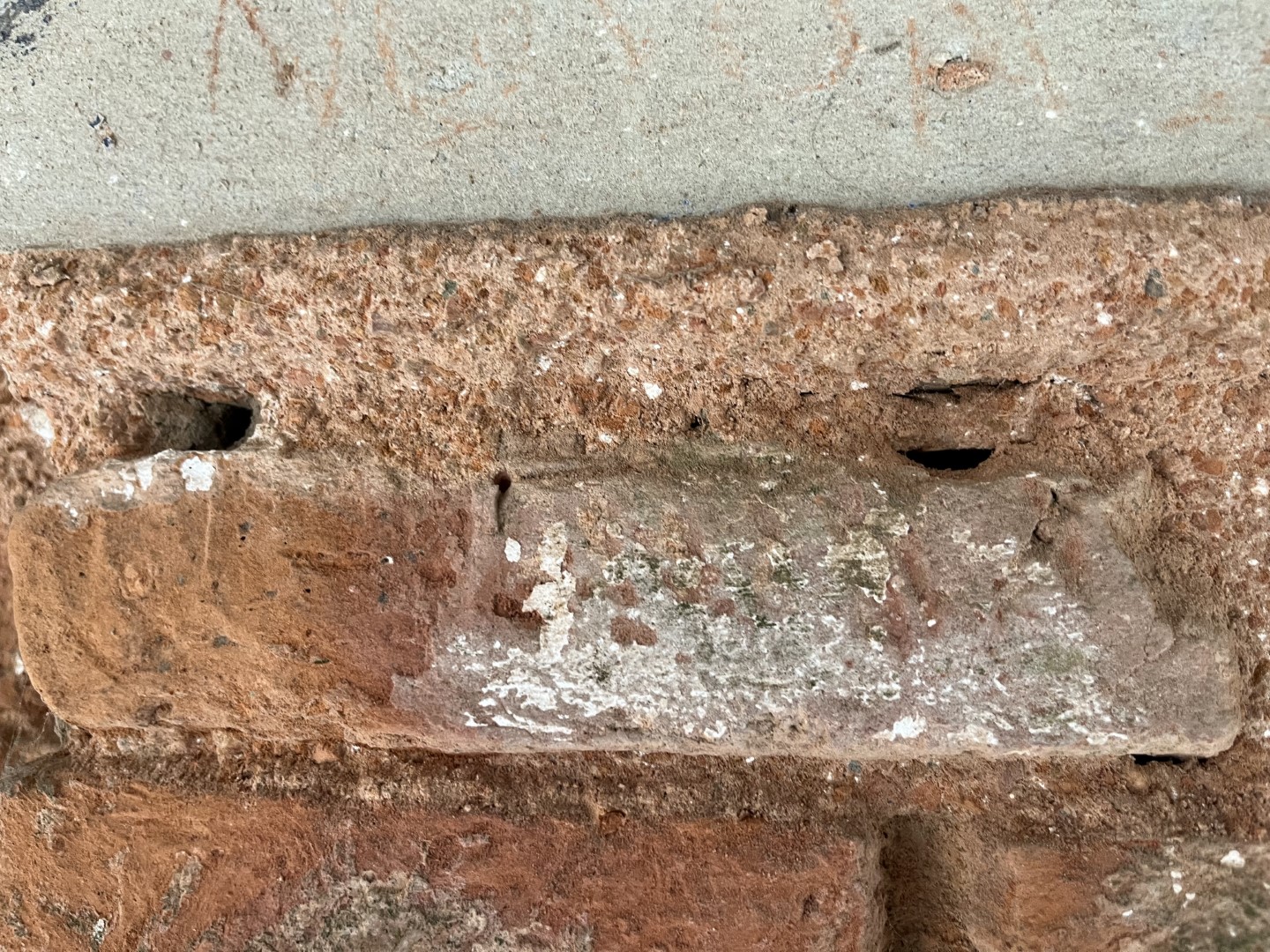

Supplement: Supplementary file 1 [file mmc1.zip › Demo_Historic_Place_Dataset/Corroded brick/IMG_3371.JPG]

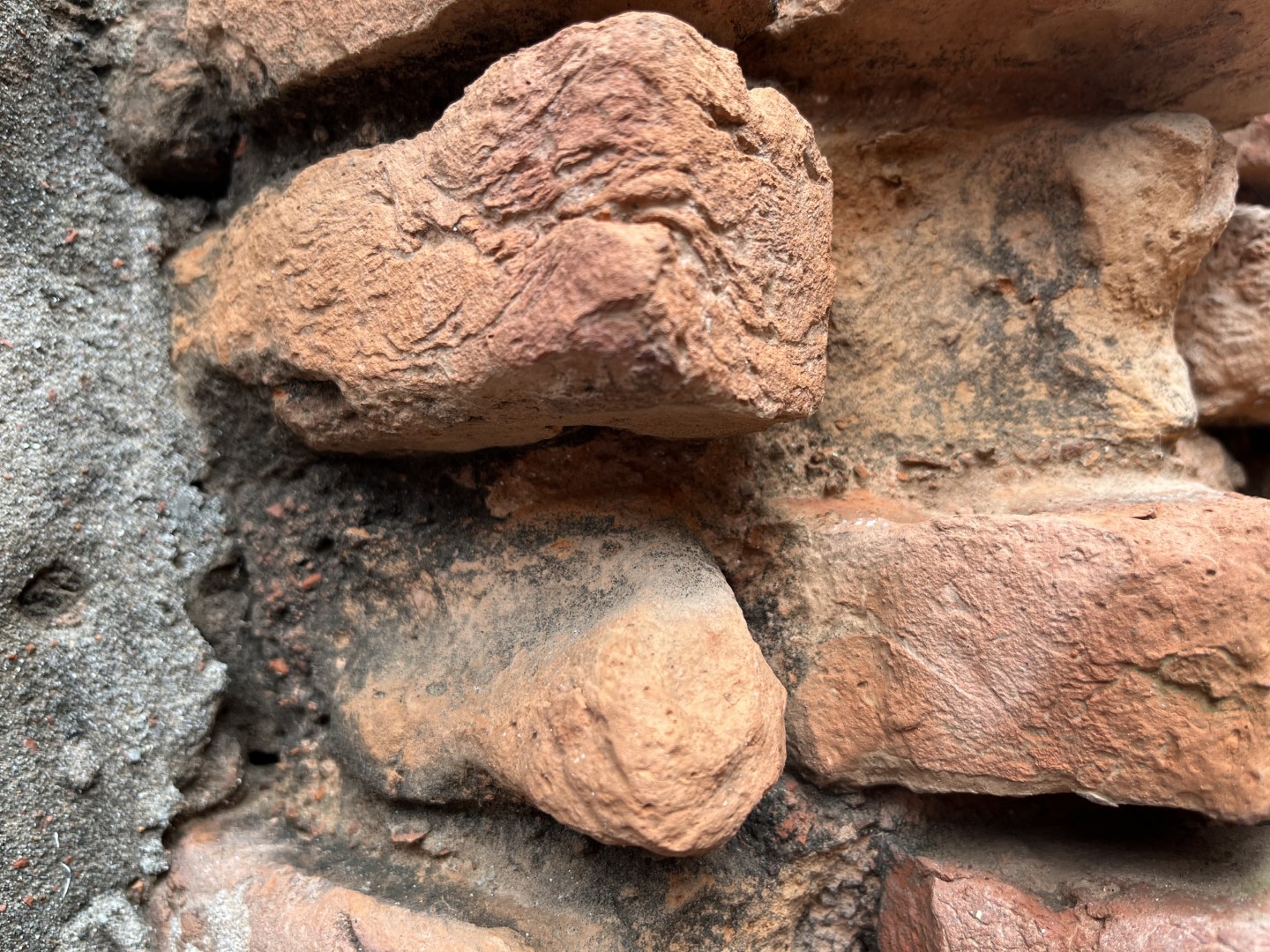

Supplement: Supplementary file 1 [file mmc1.zip › Demo_Historic_Place_Dataset/Corroded brick/IMG_3377.JPG]

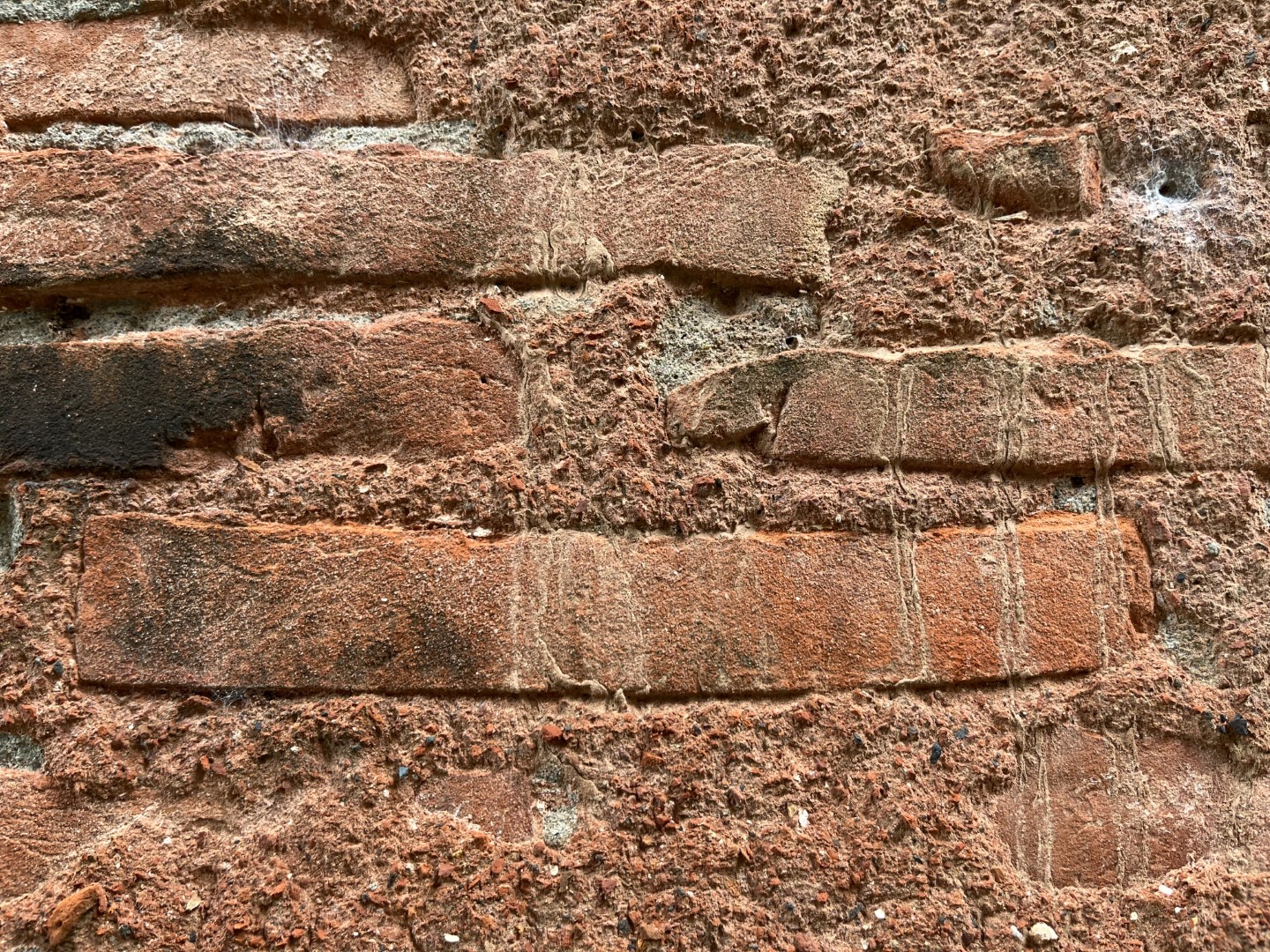

Supplement: Supplementary file 1 [file mmc1.zip › Demo_Historic_Place_Dataset/Corroded brick/IMG_3381.JPG]

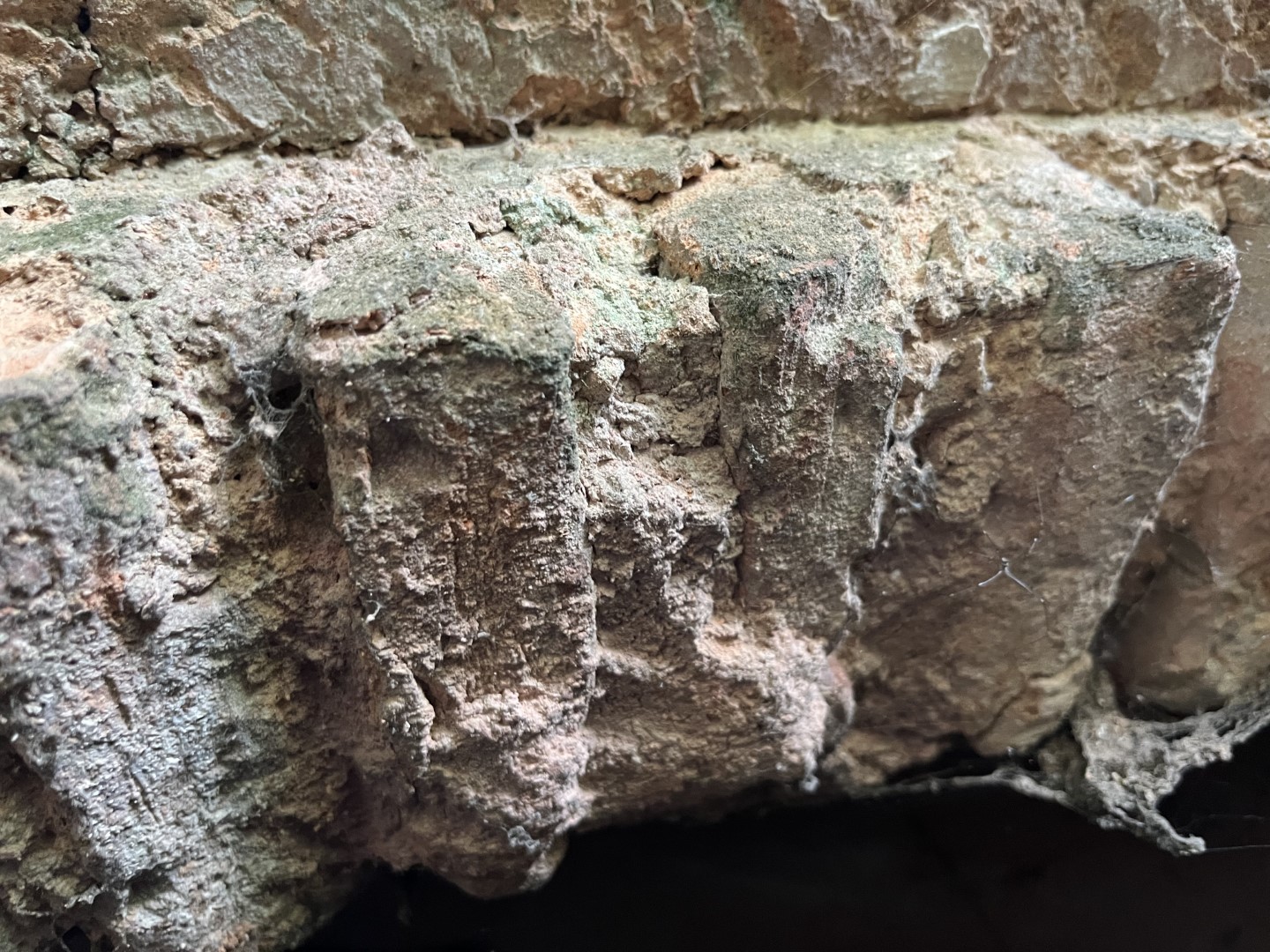

Supplement: Supplementary file 1 [file mmc1.zip › Demo_Historic_Place_Dataset/Corroded brick/IMG_3386.JPG]

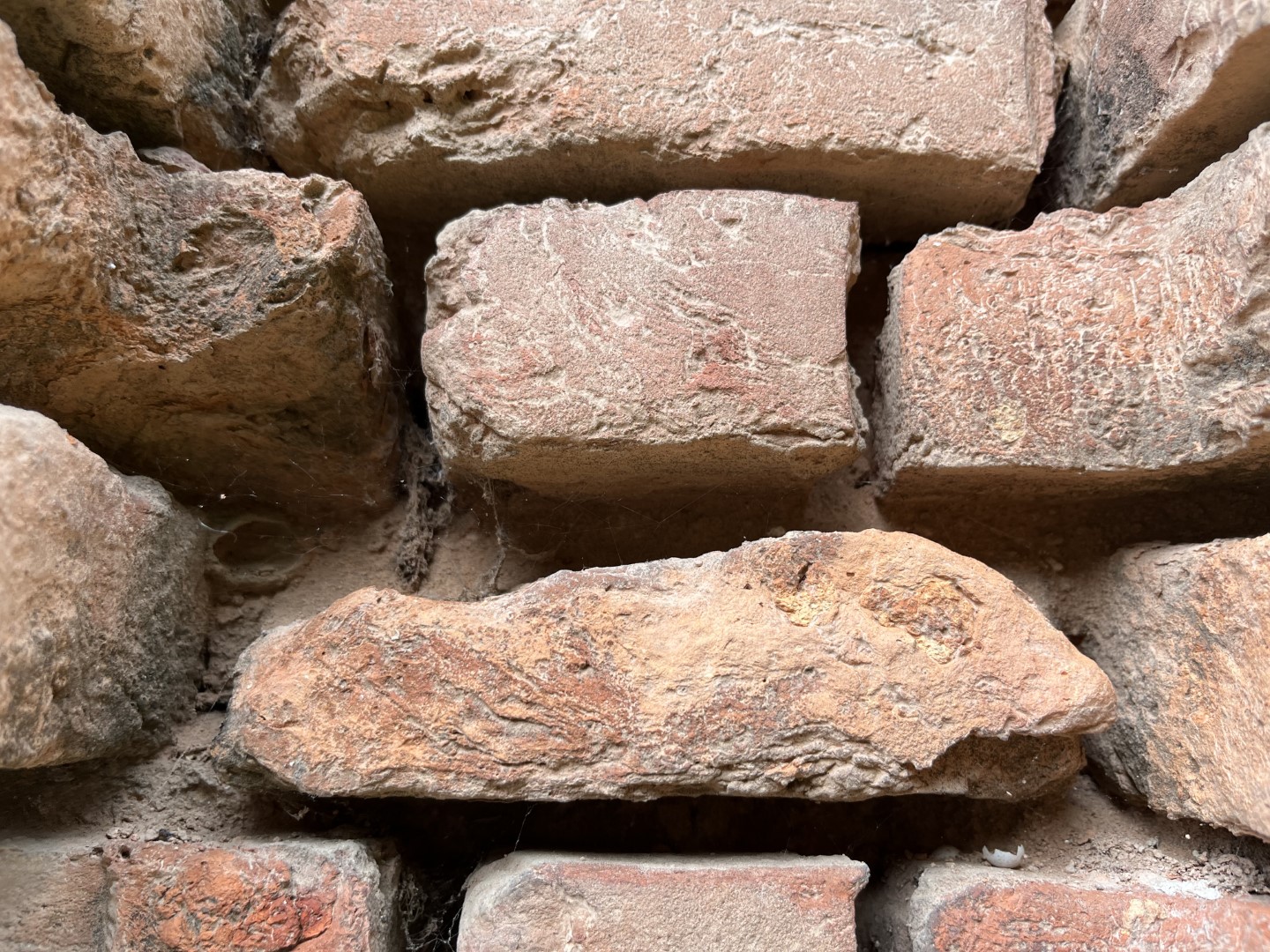

Supplement: Supplementary file 1 [file mmc1.zip › Demo_Historic_Place_Dataset/Corroded brick/IMG_3389.JPG]

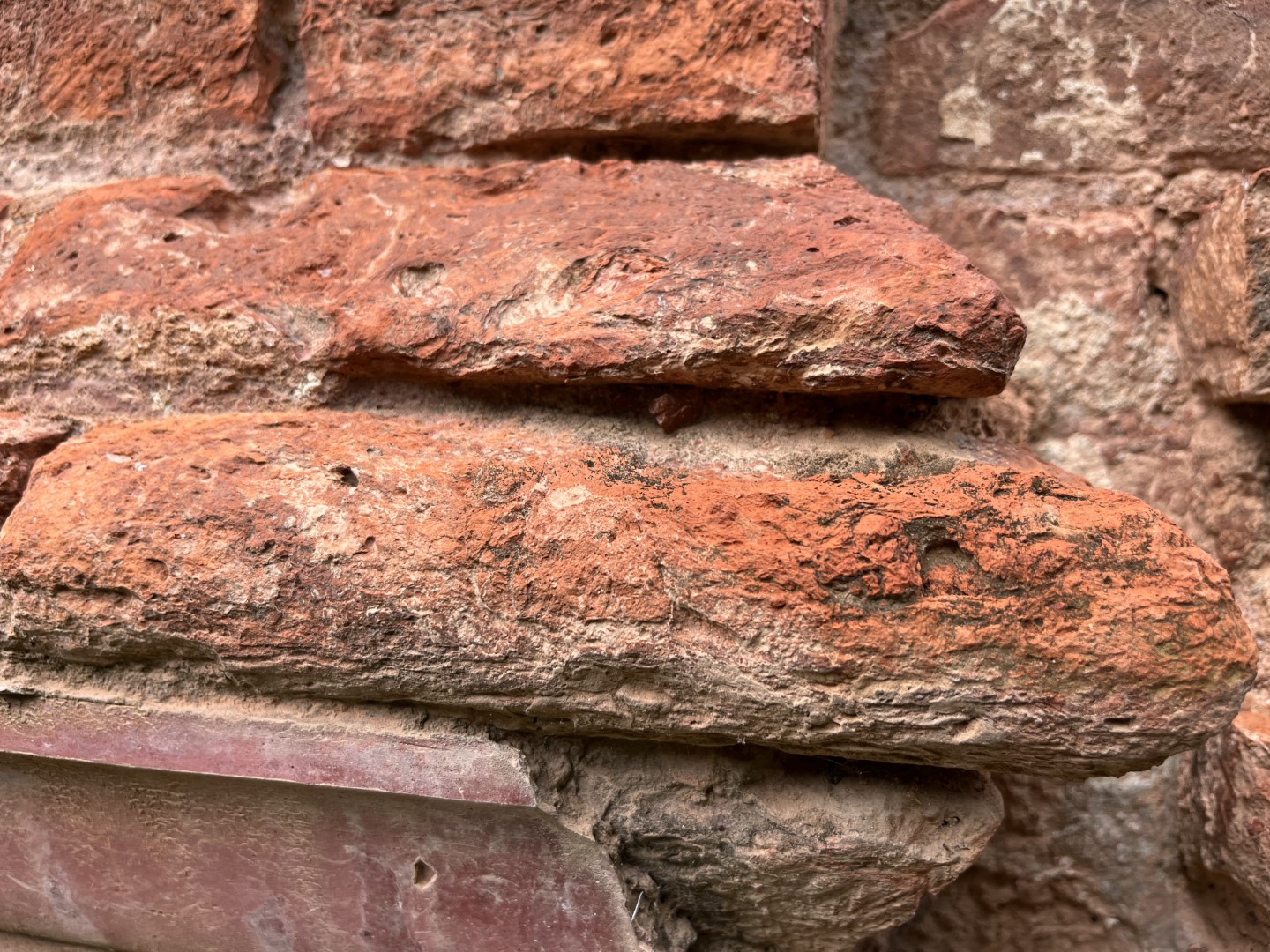

Supplement: Supplementary file 1 [file mmc1.zip › Demo_Historic_Place_Dataset/Corroded brick/IMG_3390.JPG]

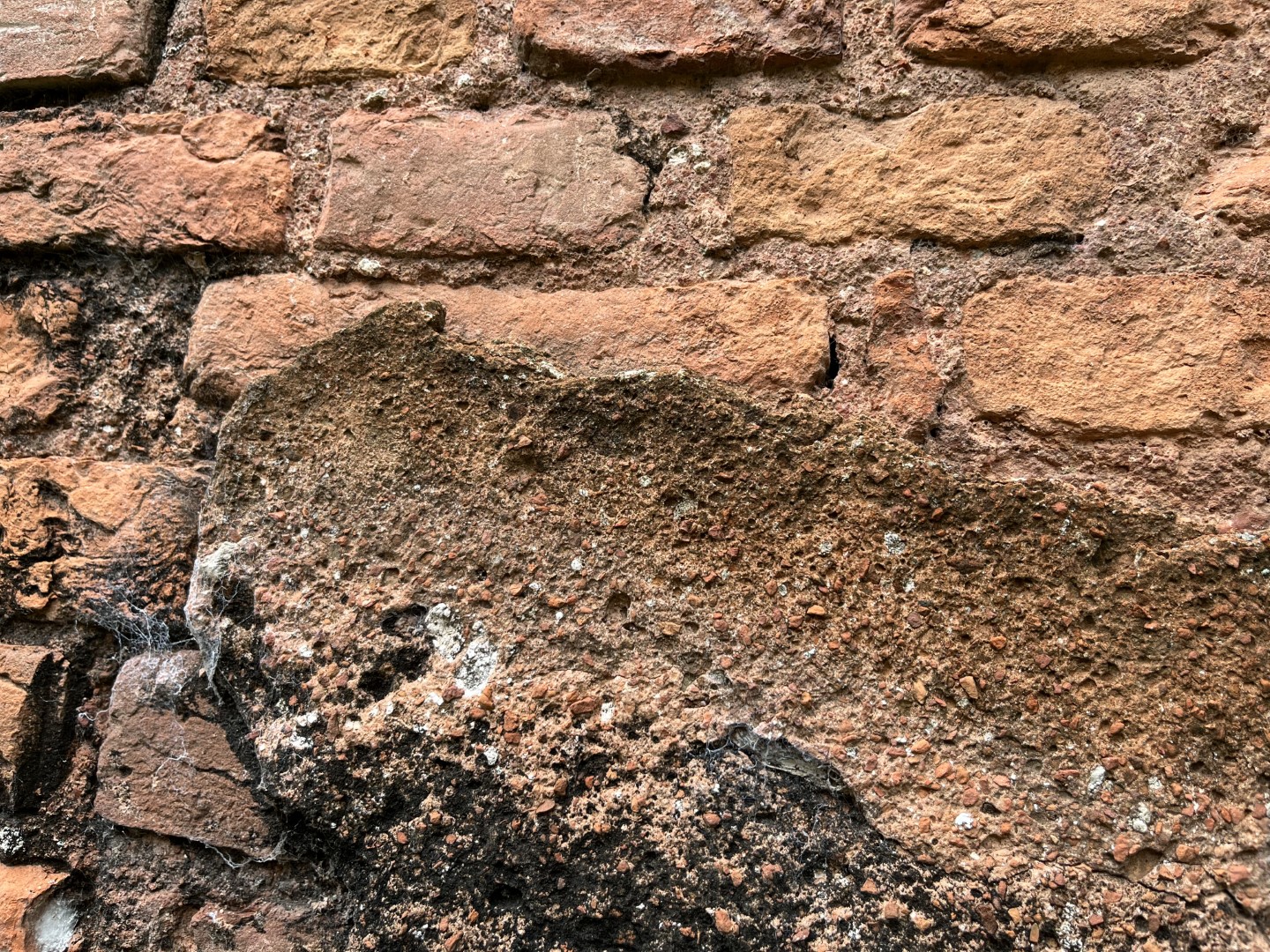

Supplement: Supplementary file 1 [file mmc1.zip › Demo_Historic_Place_Dataset/Corroded brick/IMG_3397.JPG]

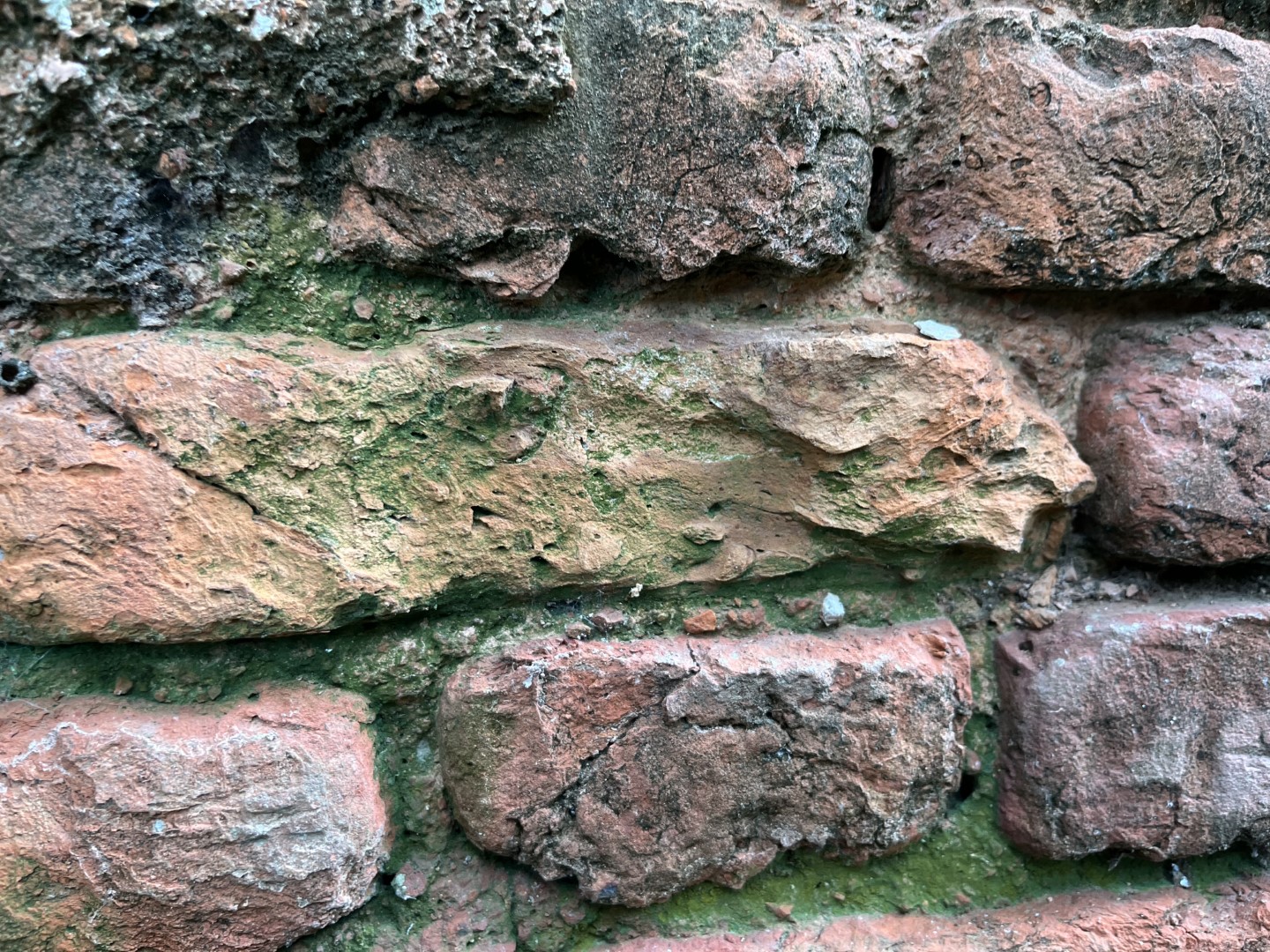

Supplement: Supplementary file 1 [file mmc1.zip › Demo_Historic_Place_Dataset/Corroded brick/IMG_3405.JPG]

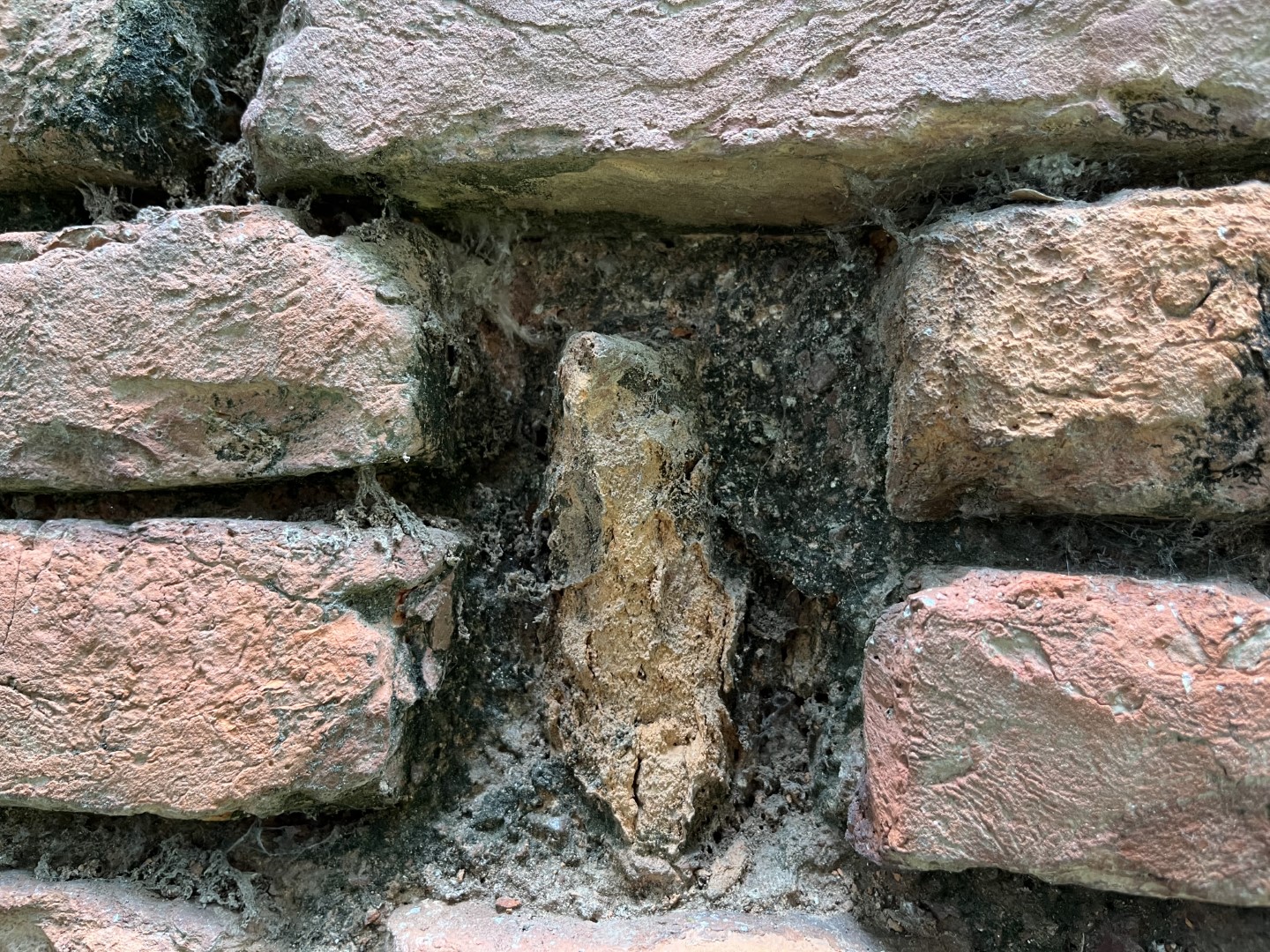

Supplement: Supplementary file 1 [file mmc1.zip › Demo_Historic_Place_Dataset/Corroded brick/IMG_3406.JPG]

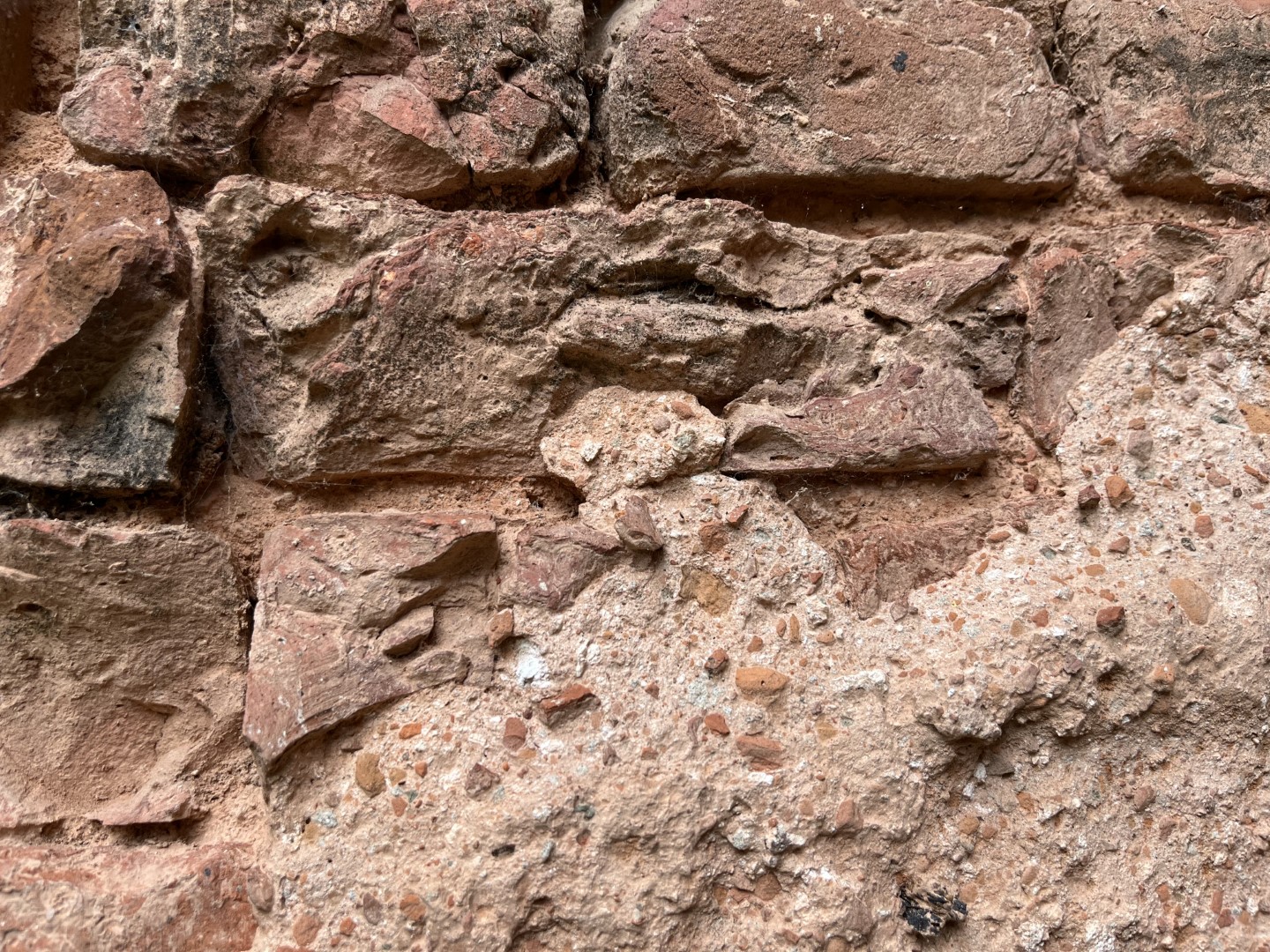

Supplement: Supplementary file 1 [file mmc1.zip › Demo_Historic_Place_Dataset/Corroded brick/IMG_3410.JPG]

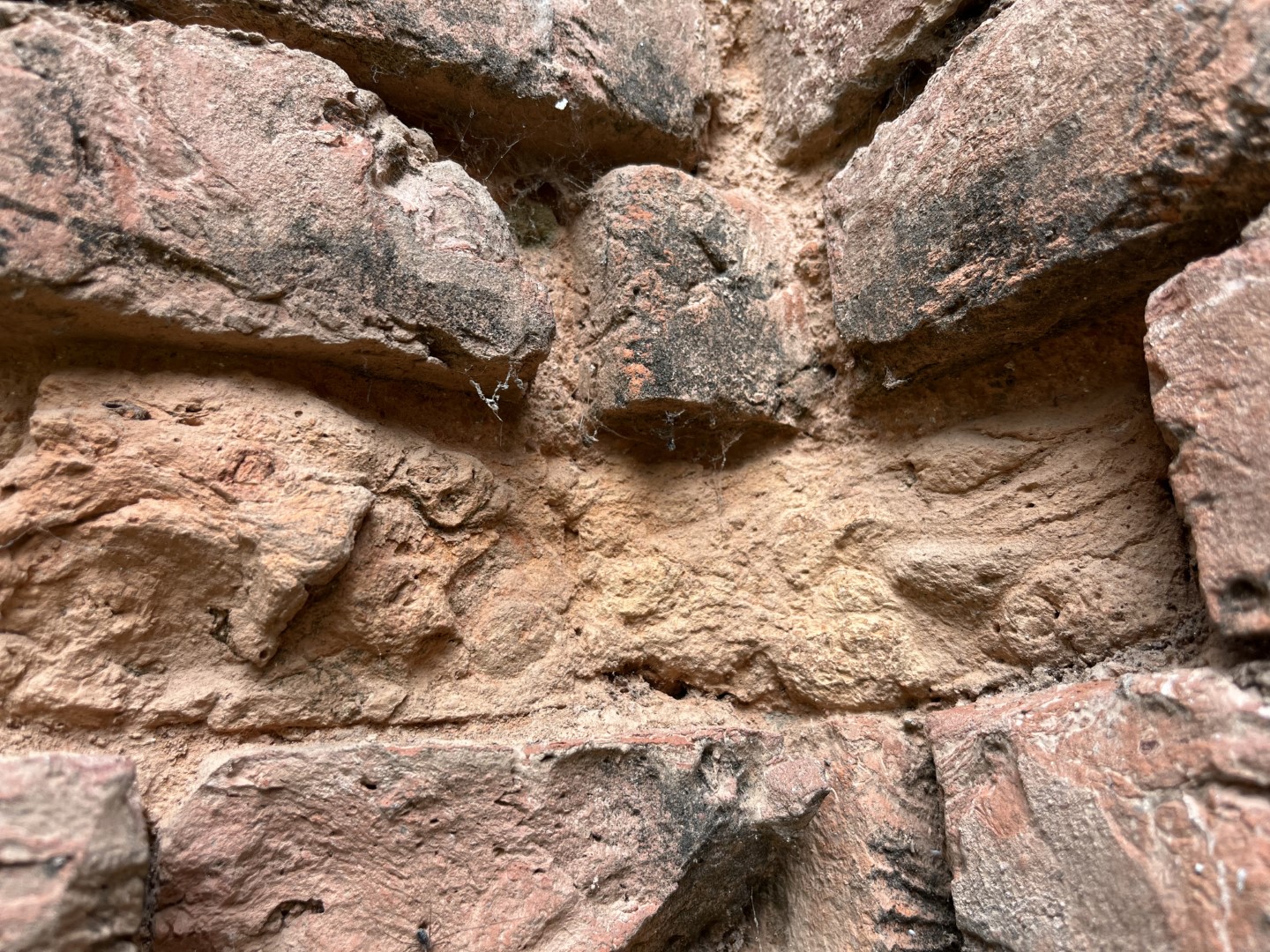

Supplement: Supplementary file 1 [file mmc1.zip › Demo_Historic_Place_Dataset/Corroded brick/IMG_3411.JPG]

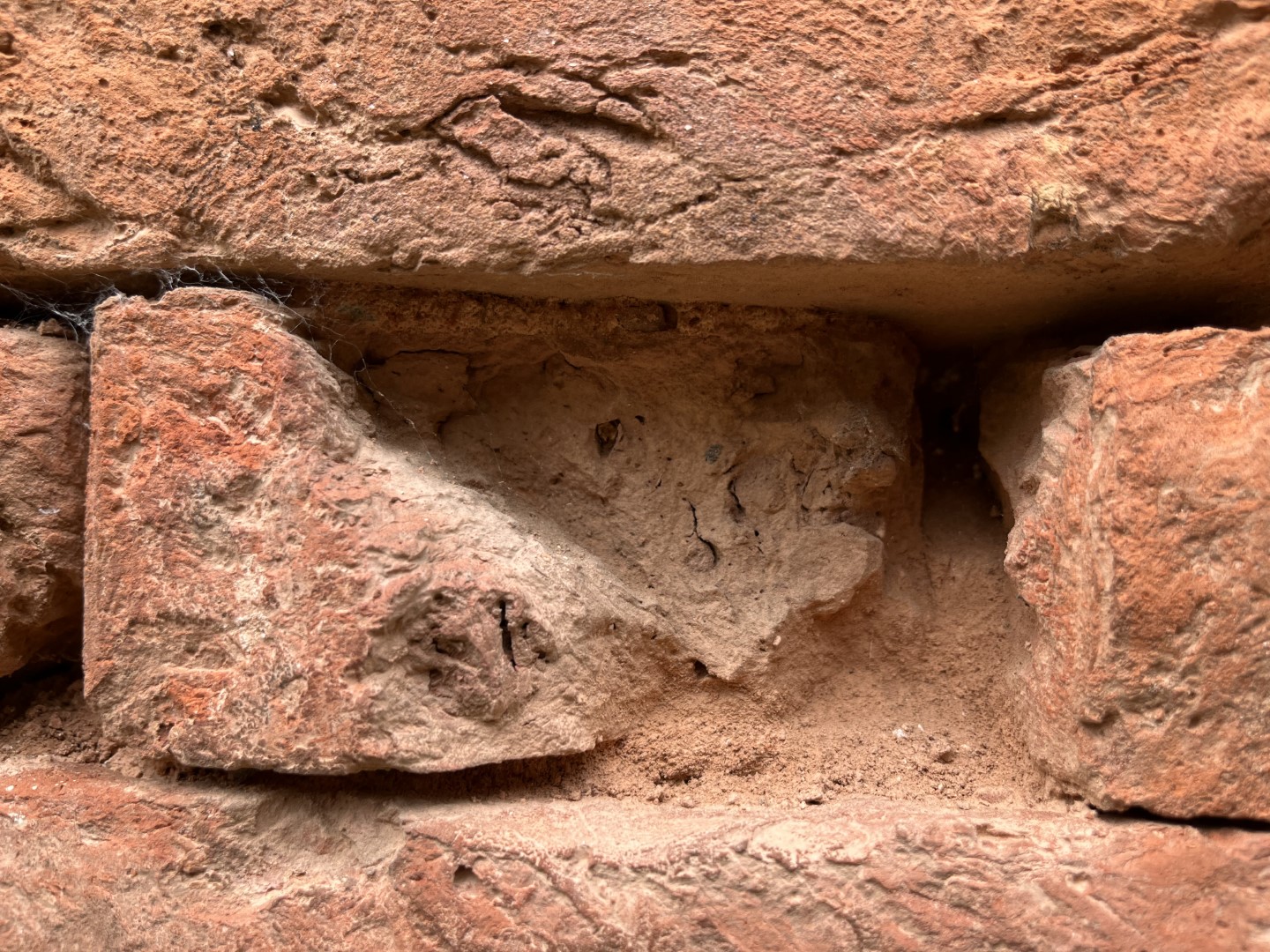

Supplement: Supplementary file 1 [file mmc1.zip › Demo_Historic_Place_Dataset/Corroded brick/IMG_3414.JPG]

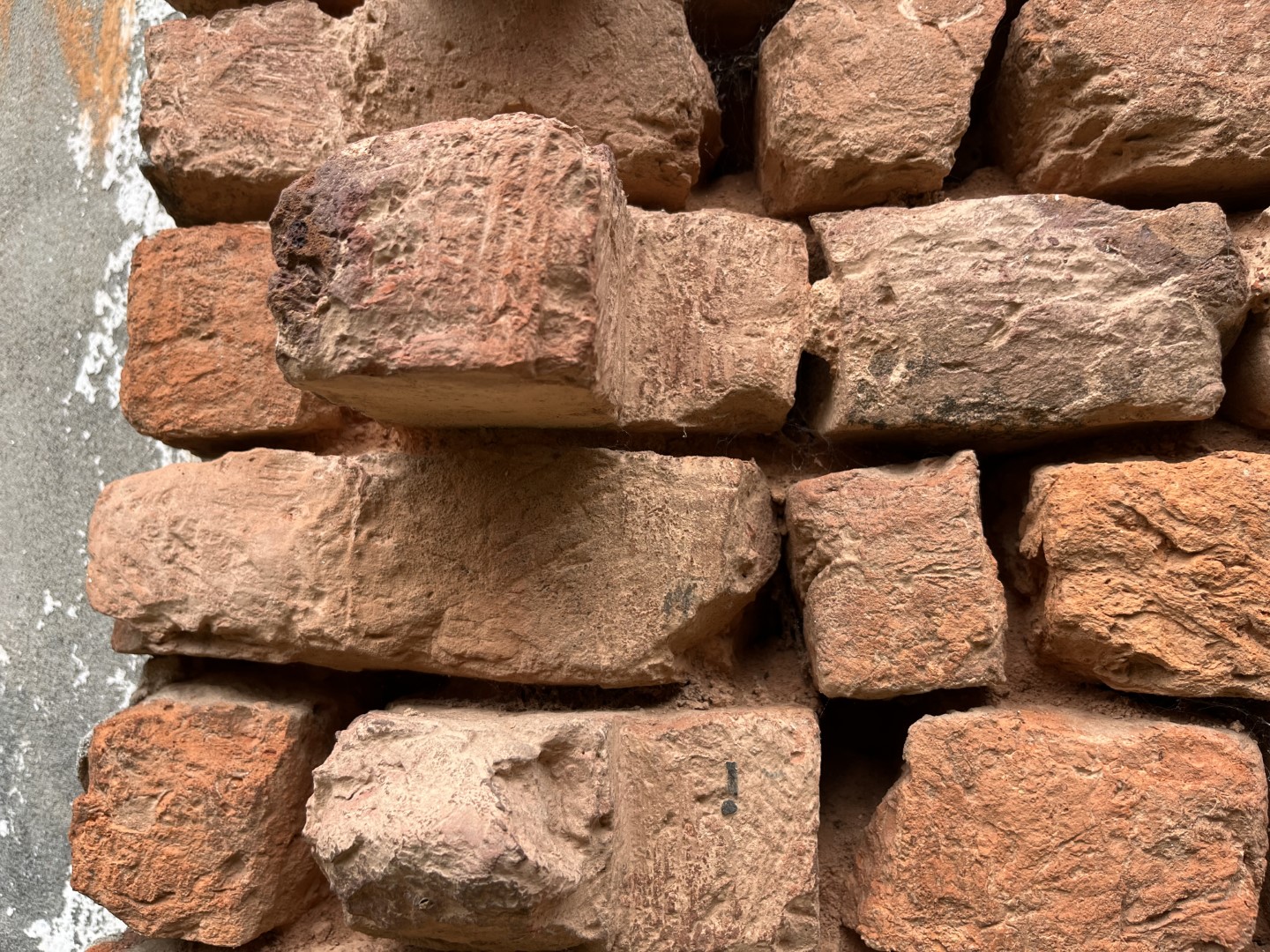

Supplement: Supplementary file 1 [file mmc1.zip › Demo_Historic_Place_Dataset/Corroded brick/IMG_3415.JPG]

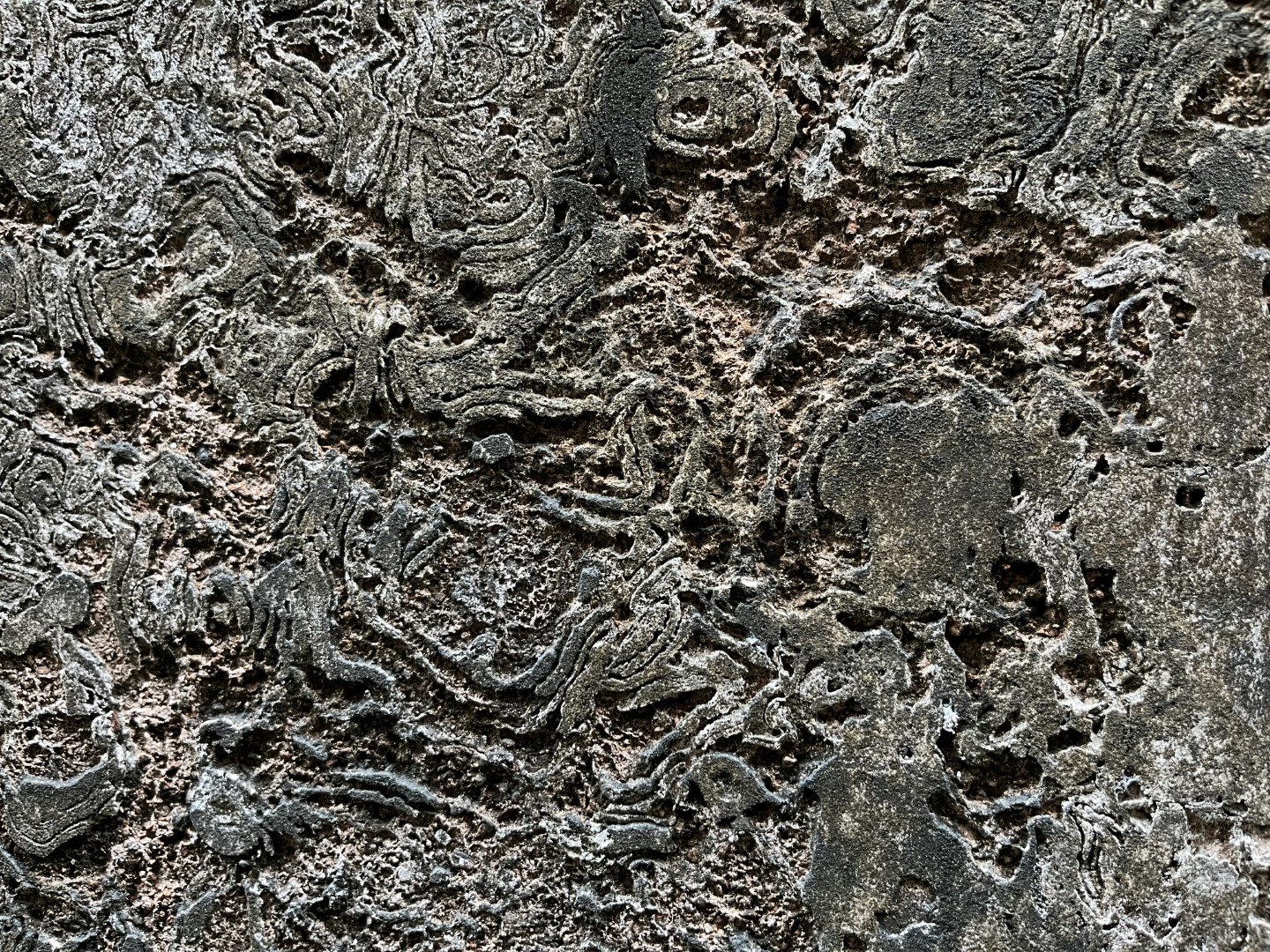

Supplement: Supplementary file 1 [file mmc1.zip › Demo_Historic_Place_Dataset/Corroded plaster/IMG_3361.JPG]

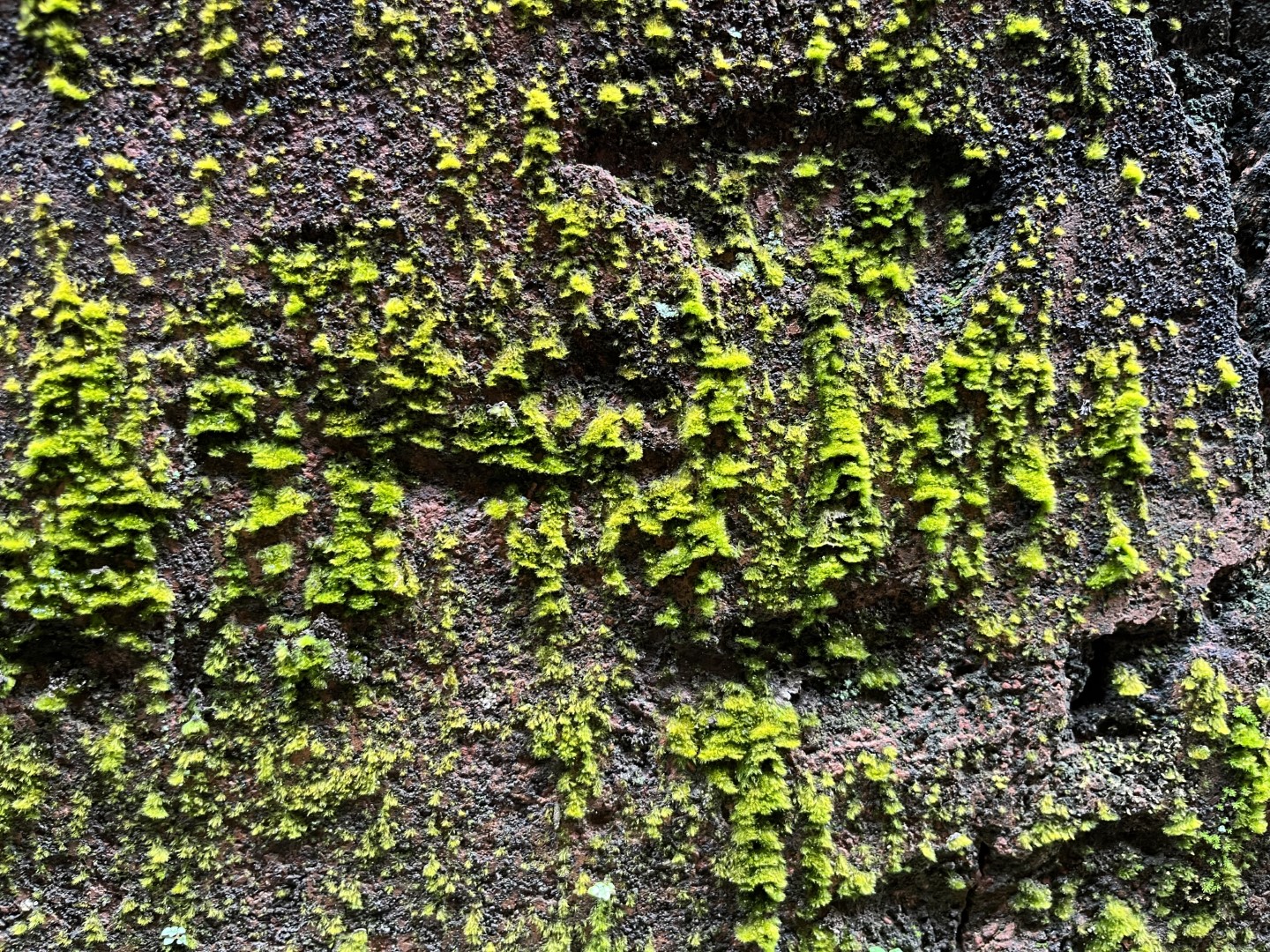

Supplement: Supplementary file 1 [file mmc1.zip › Demo_Historic_Place_Dataset/Corroded plaster/IMG_3362.JPG]

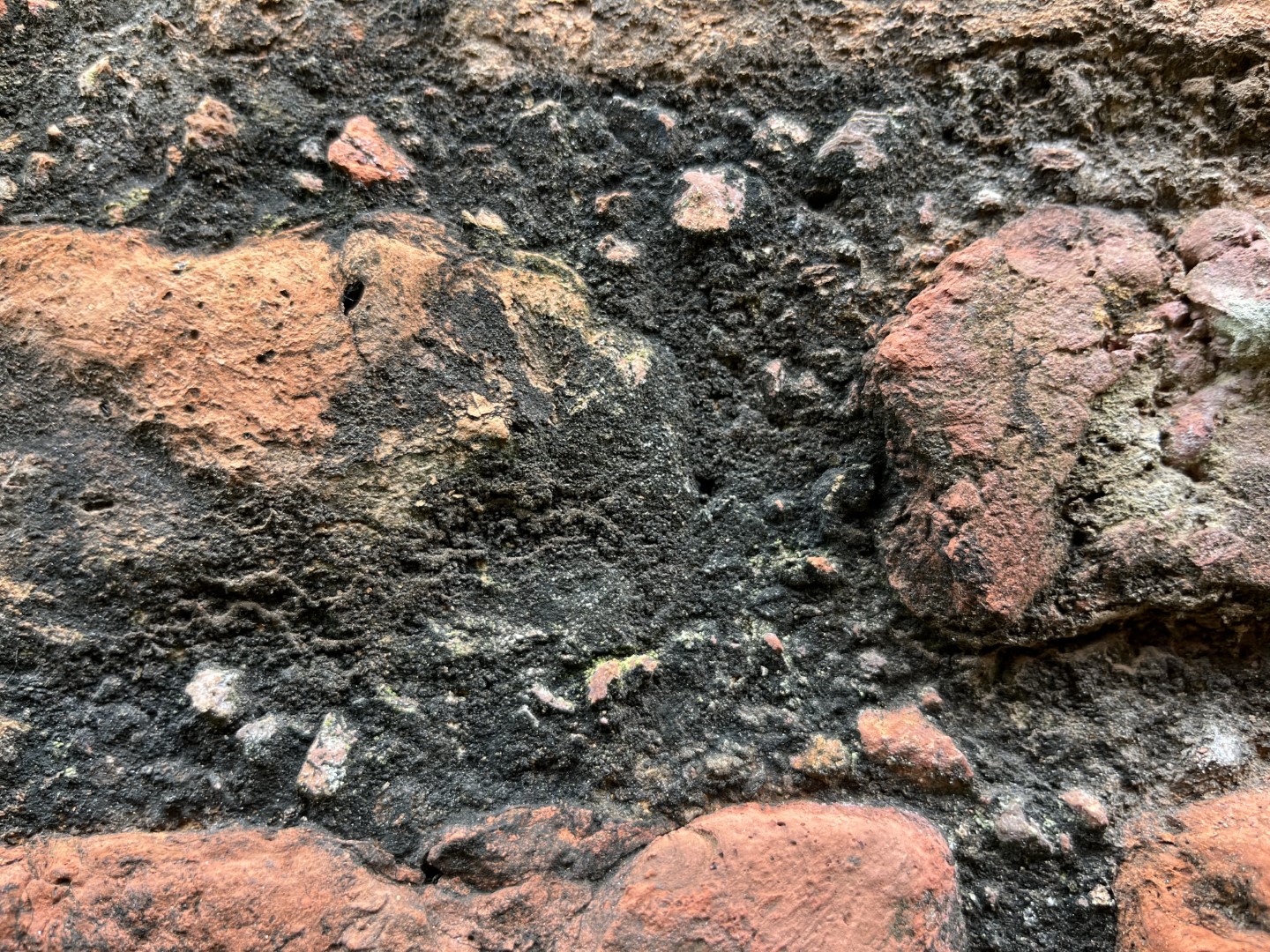

Supplement: Supplementary file 1 [file mmc1.zip › Demo_Historic_Place_Dataset/Corroded plaster/IMG_3364.JPG]

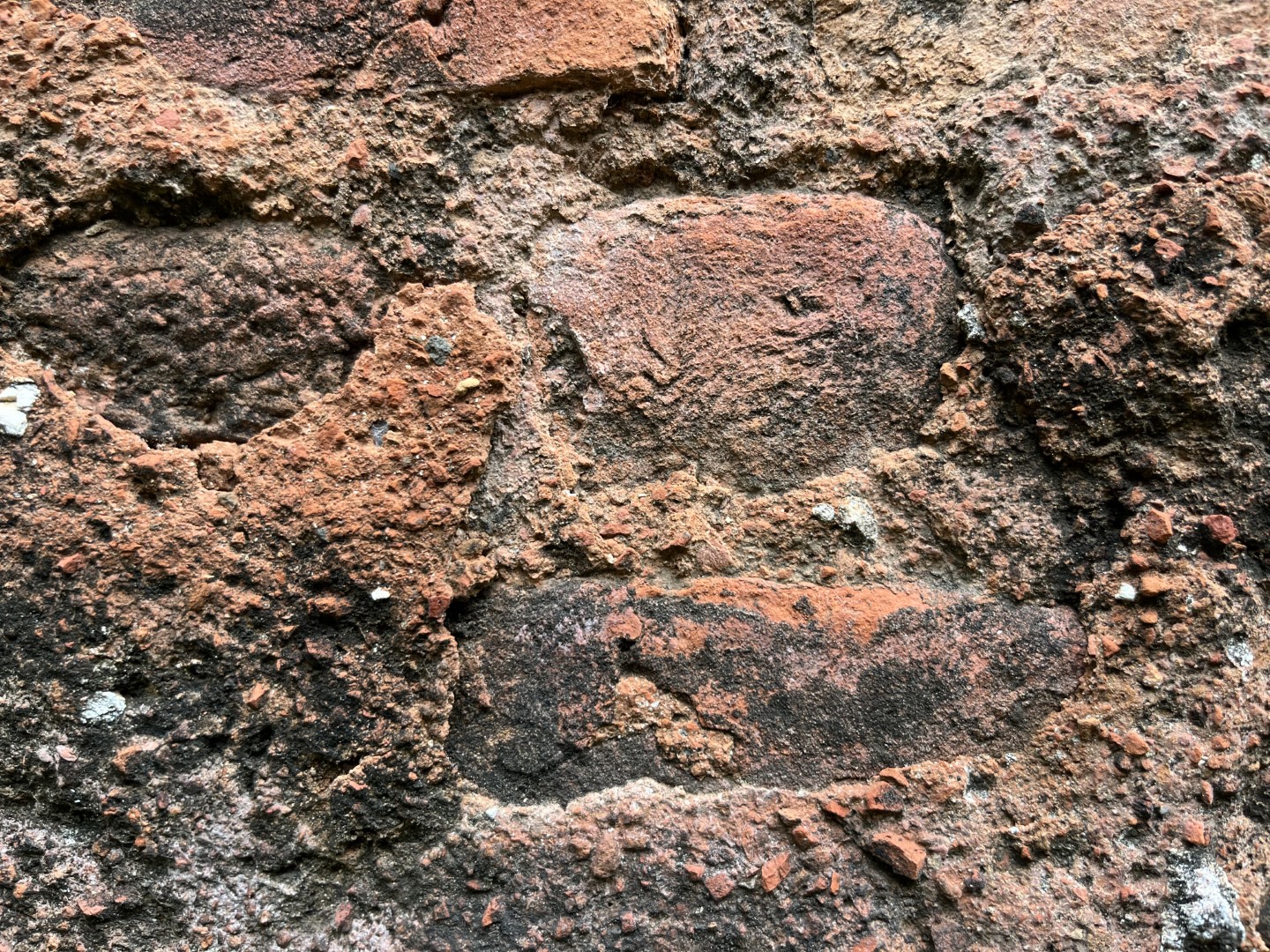

Supplement: Supplementary file 1 [file mmc1.zip › Demo_Historic_Place_Dataset/Corroded plaster/IMG_3368.JPG]

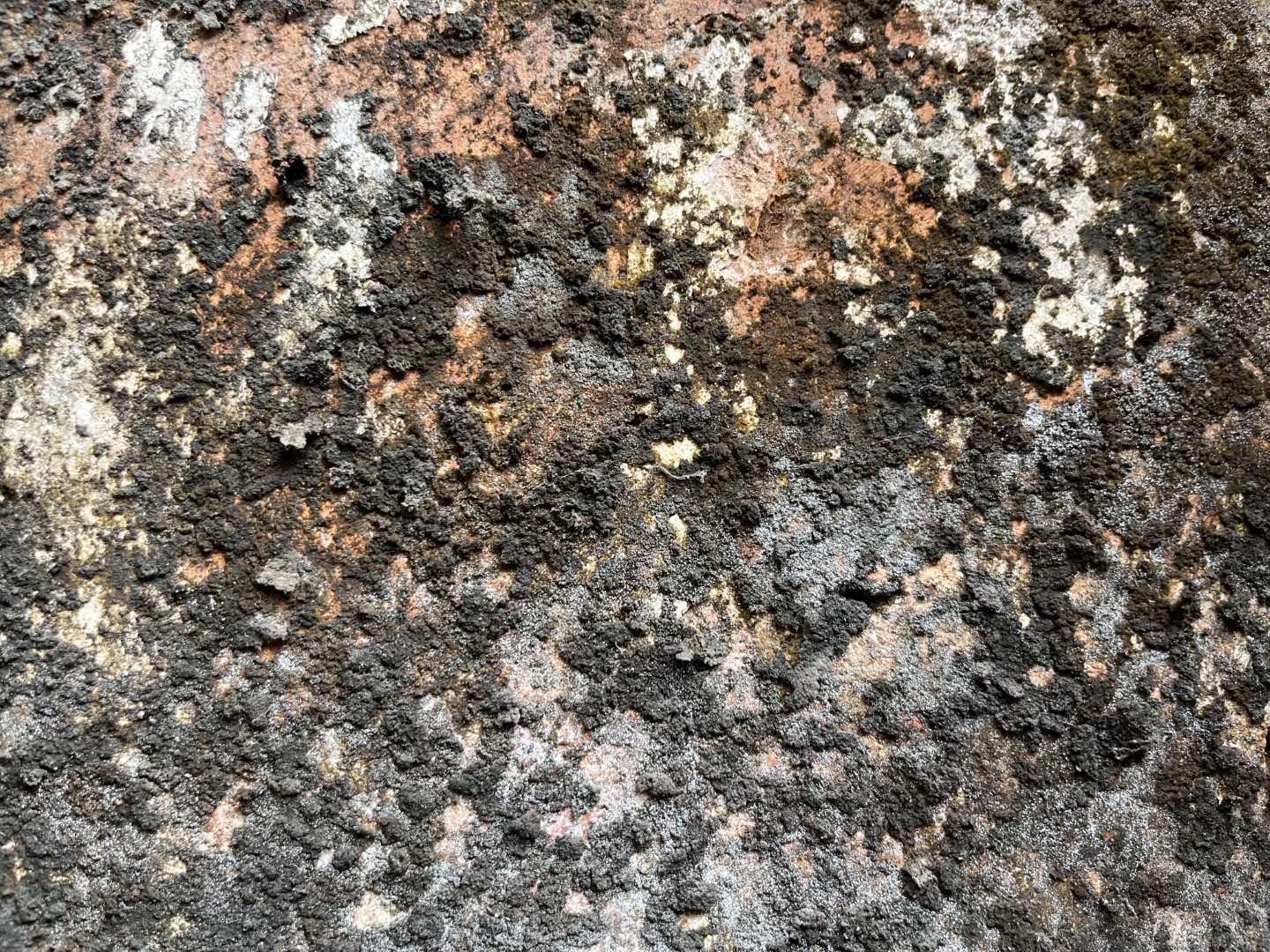

Supplement: Supplementary file 1 [file mmc1.zip › Demo_Historic_Place_Dataset/Corroded plaster/IMG_3372.JPG]

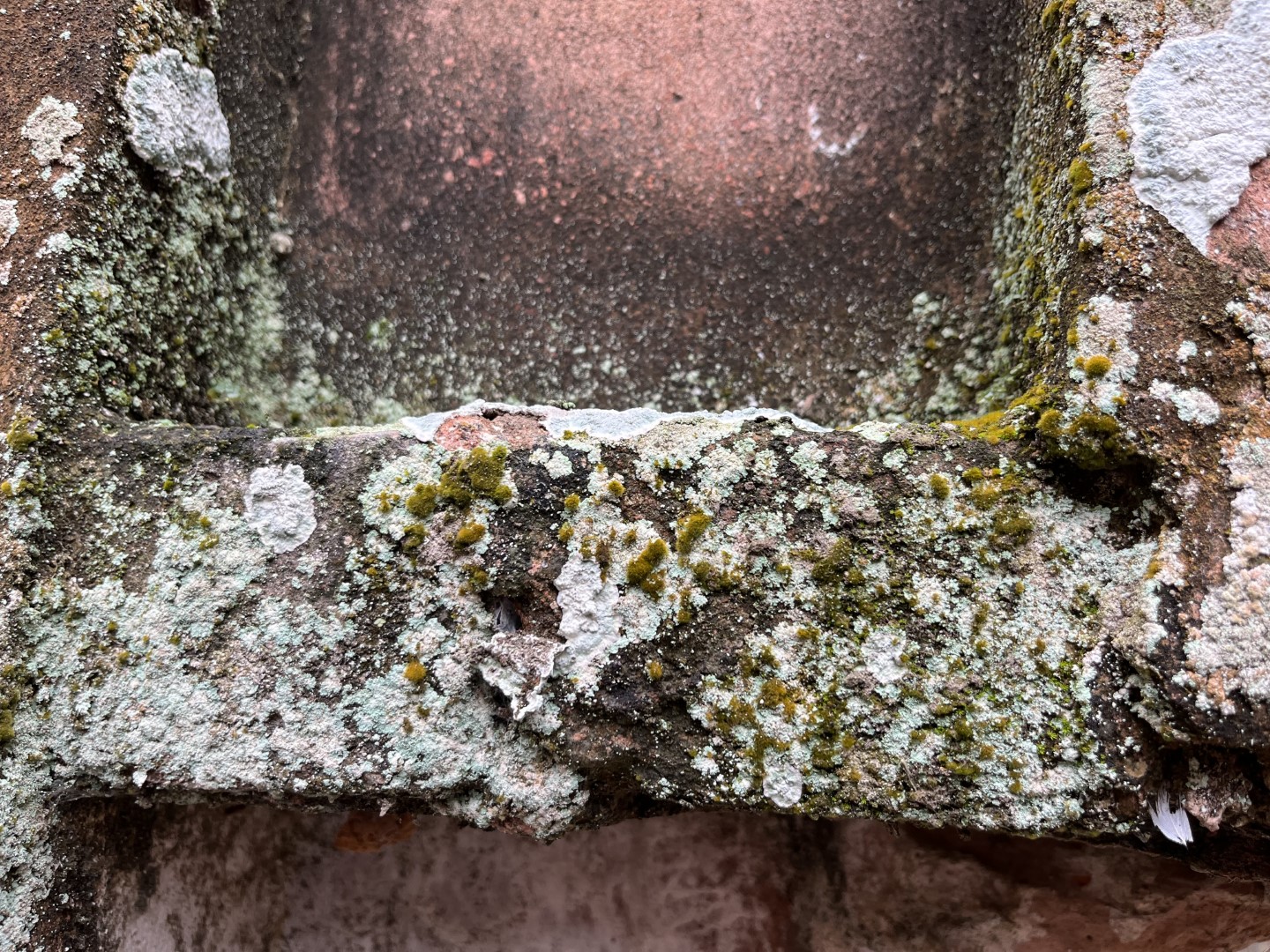

Supplement: Supplementary file 1 [file mmc1.zip › Demo_Historic_Place_Dataset/Corroded plaster/IMG_3373.JPG]

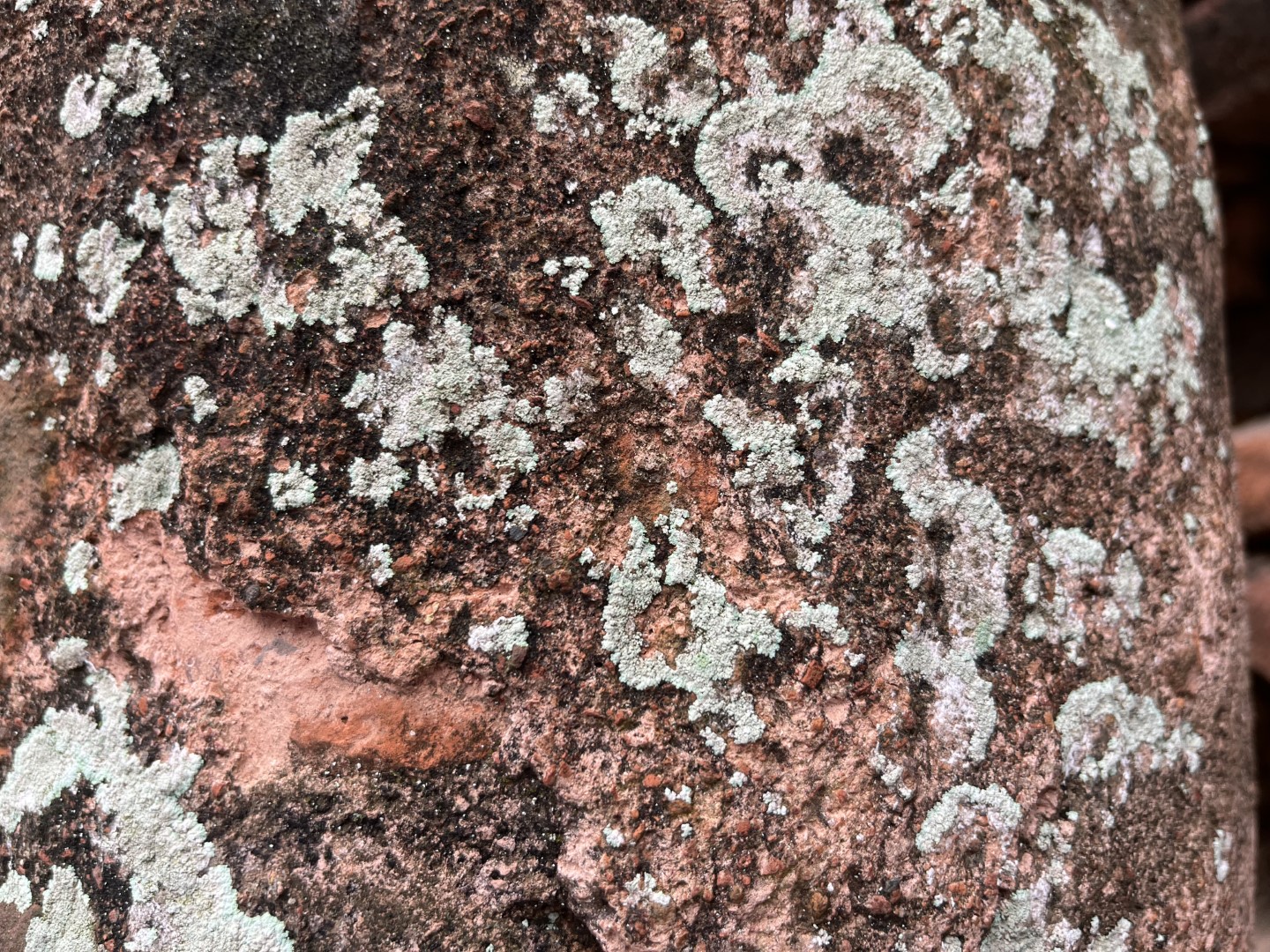

Supplement: Supplementary file 1 [file mmc1.zip › Demo_Historic_Place_Dataset/Corroded plaster/IMG_3376.JPG]

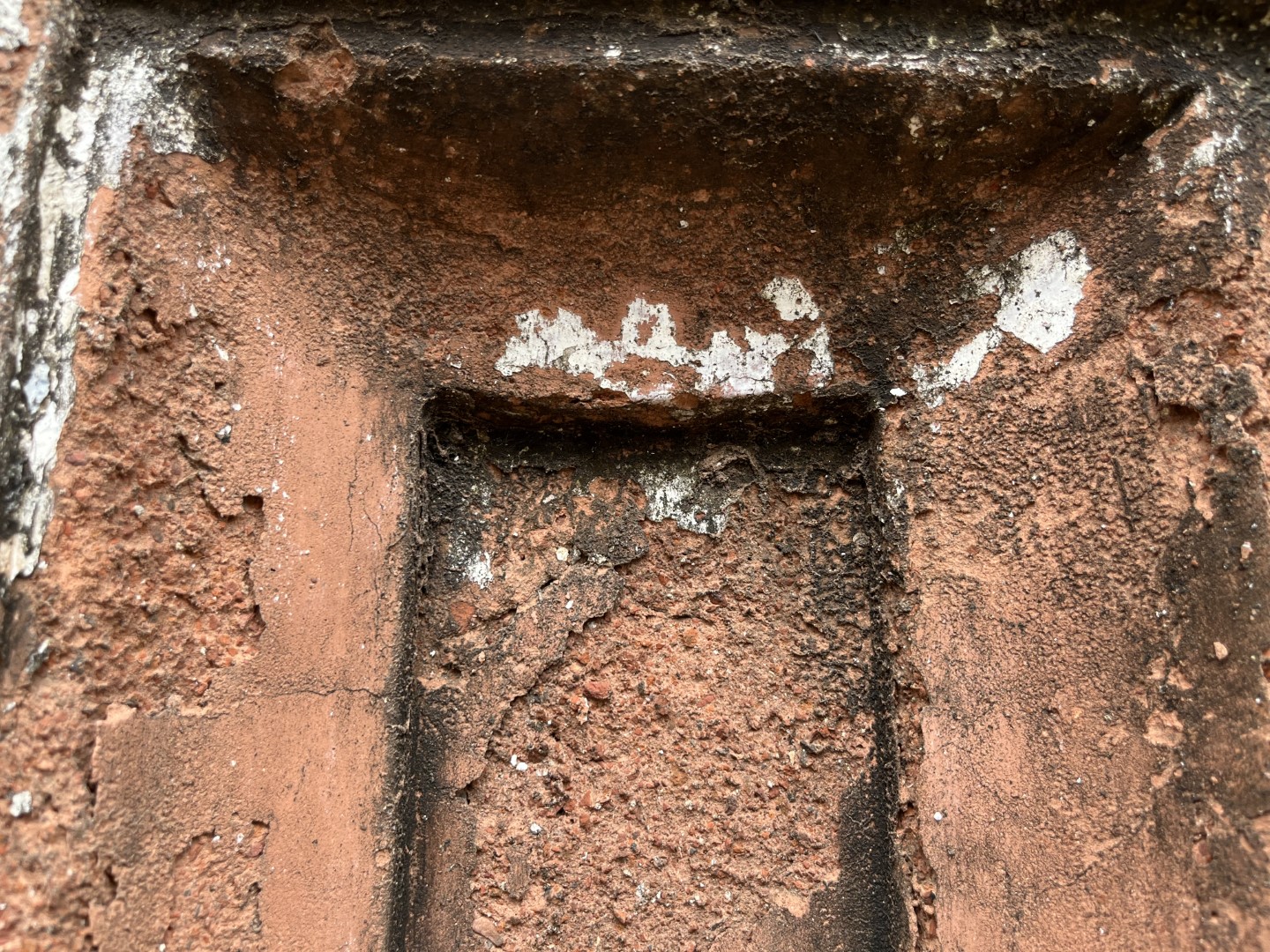

Supplement: Supplementary file 1 [file mmc1.zip › Demo_Historic_Place_Dataset/Corroded plaster/IMG_3378.JPG]

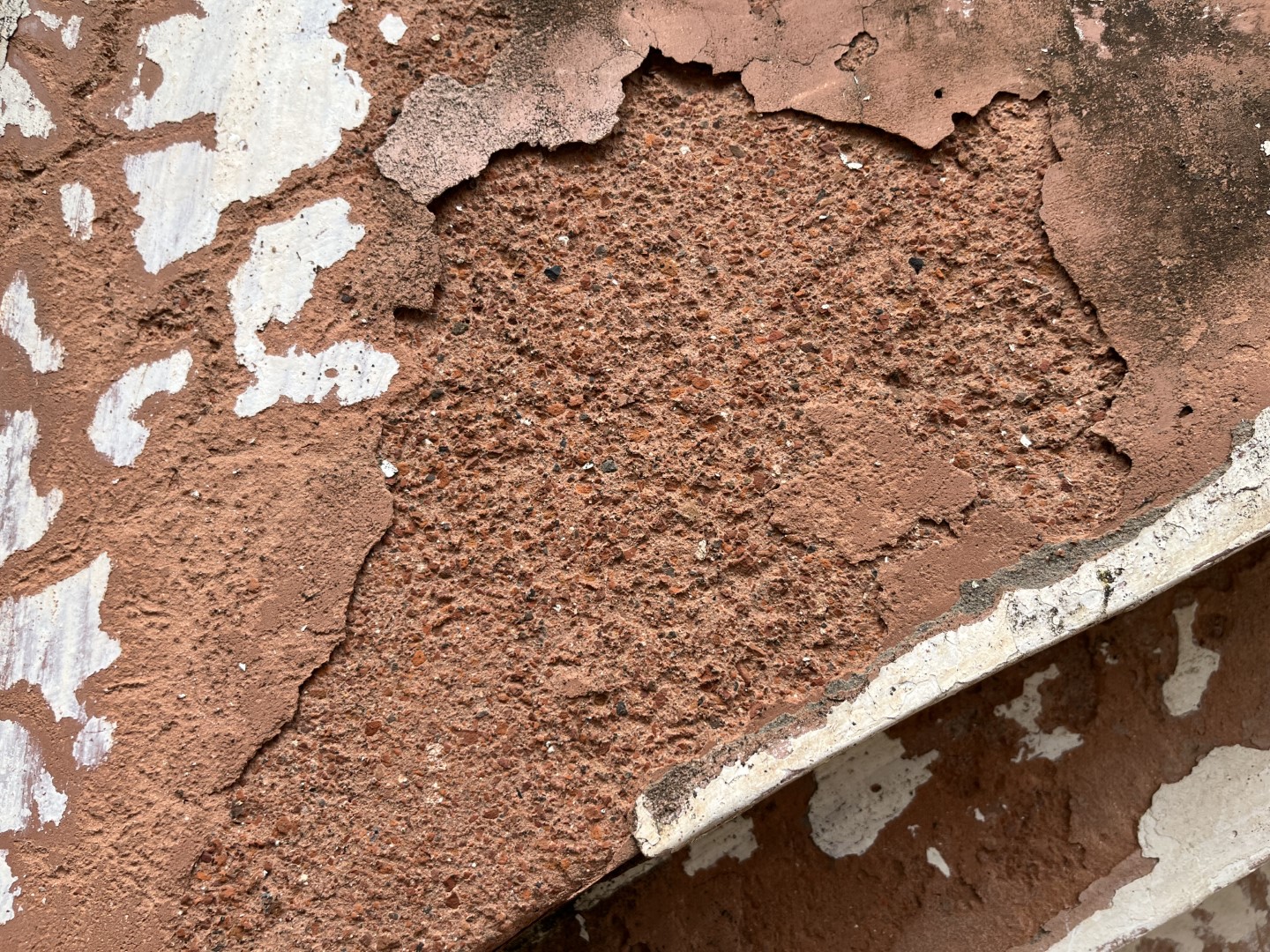

Supplement: Supplementary file 1 [file mmc1.zip › Demo_Historic_Place_Dataset/Corroded plaster/IMG_3379.JPG]

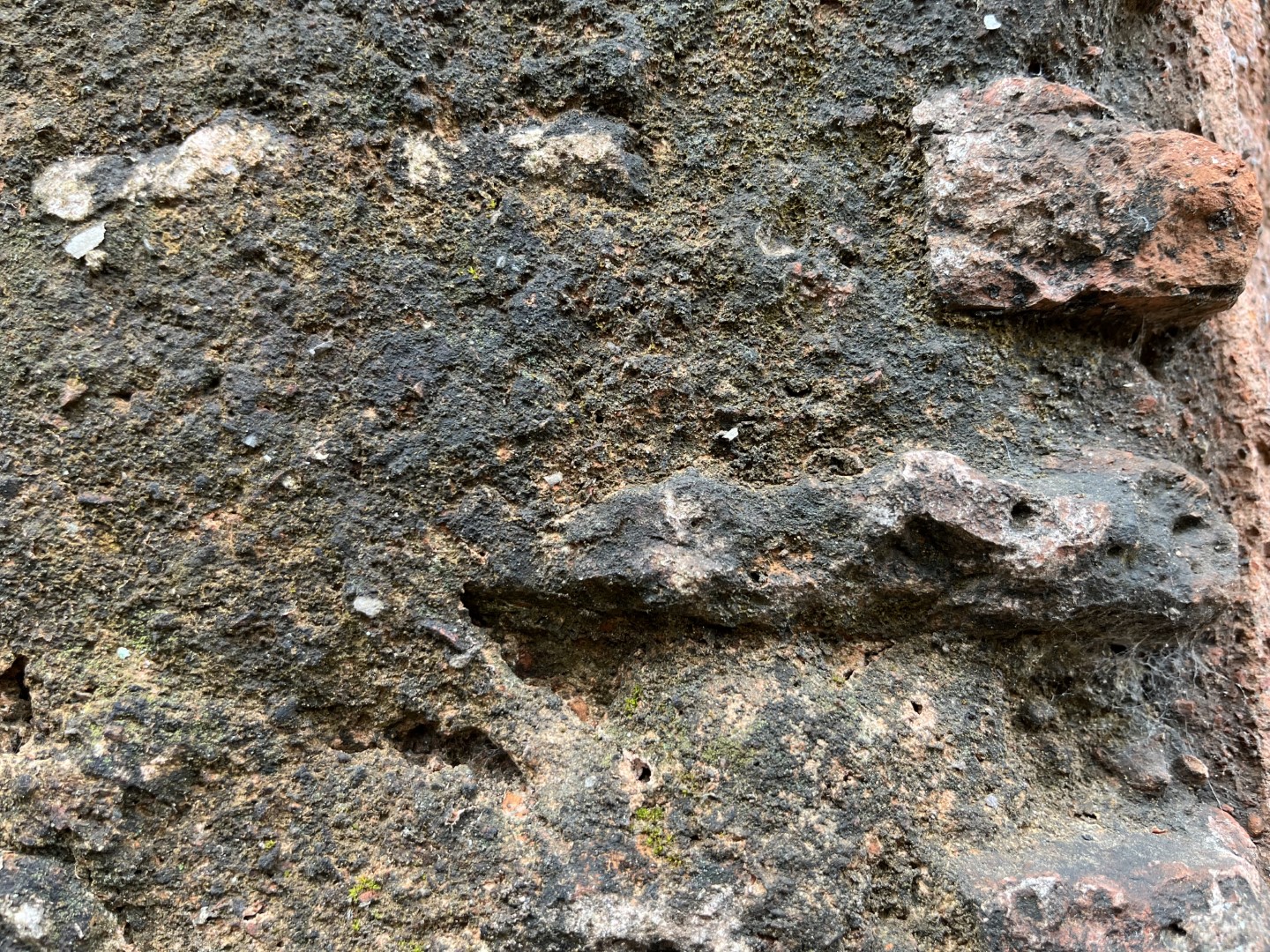

Supplement: Supplementary file 1 [file mmc1.zip › Demo_Historic_Place_Dataset/Corroded plaster/IMG_3380.JPG]

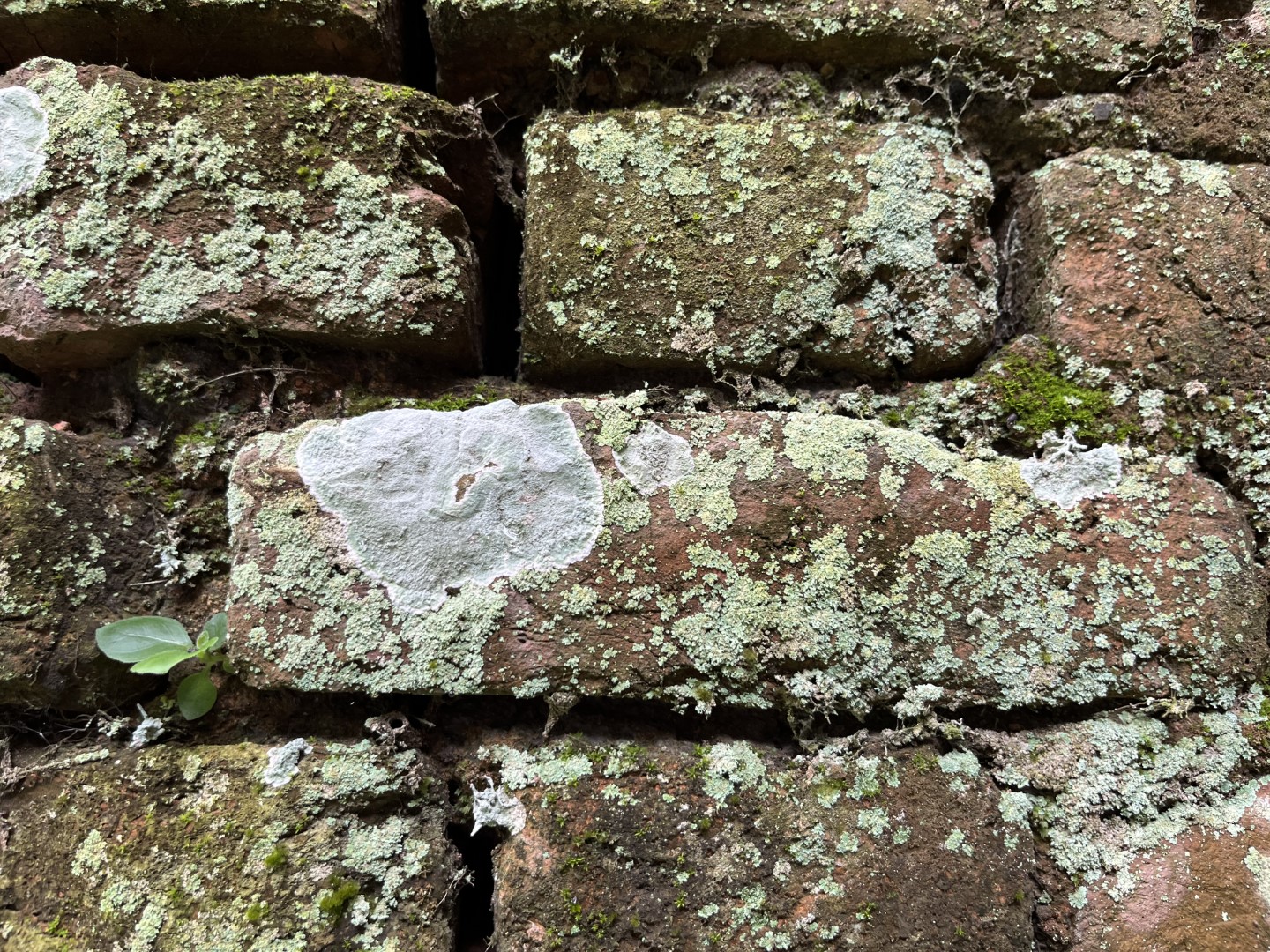

Supplement: Supplementary file 1 [file mmc1.zip › Demo_Historic_Place_Dataset/Corroded plaster/IMG_3398.JPG]

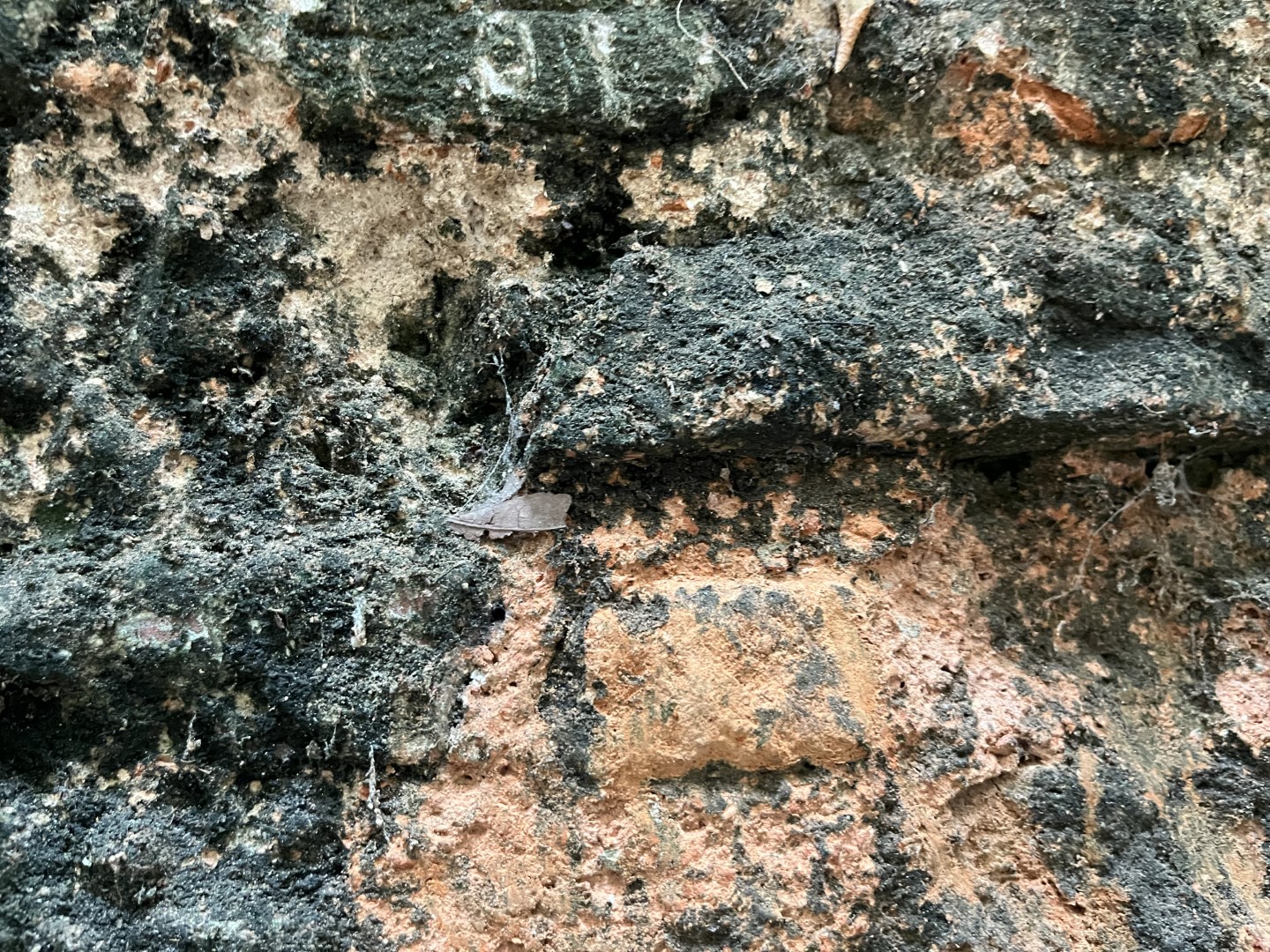

Supplement: Supplementary file 1 [file mmc1.zip › Demo_Historic_Place_Dataset/Fungus/IMG_3669.JPG]

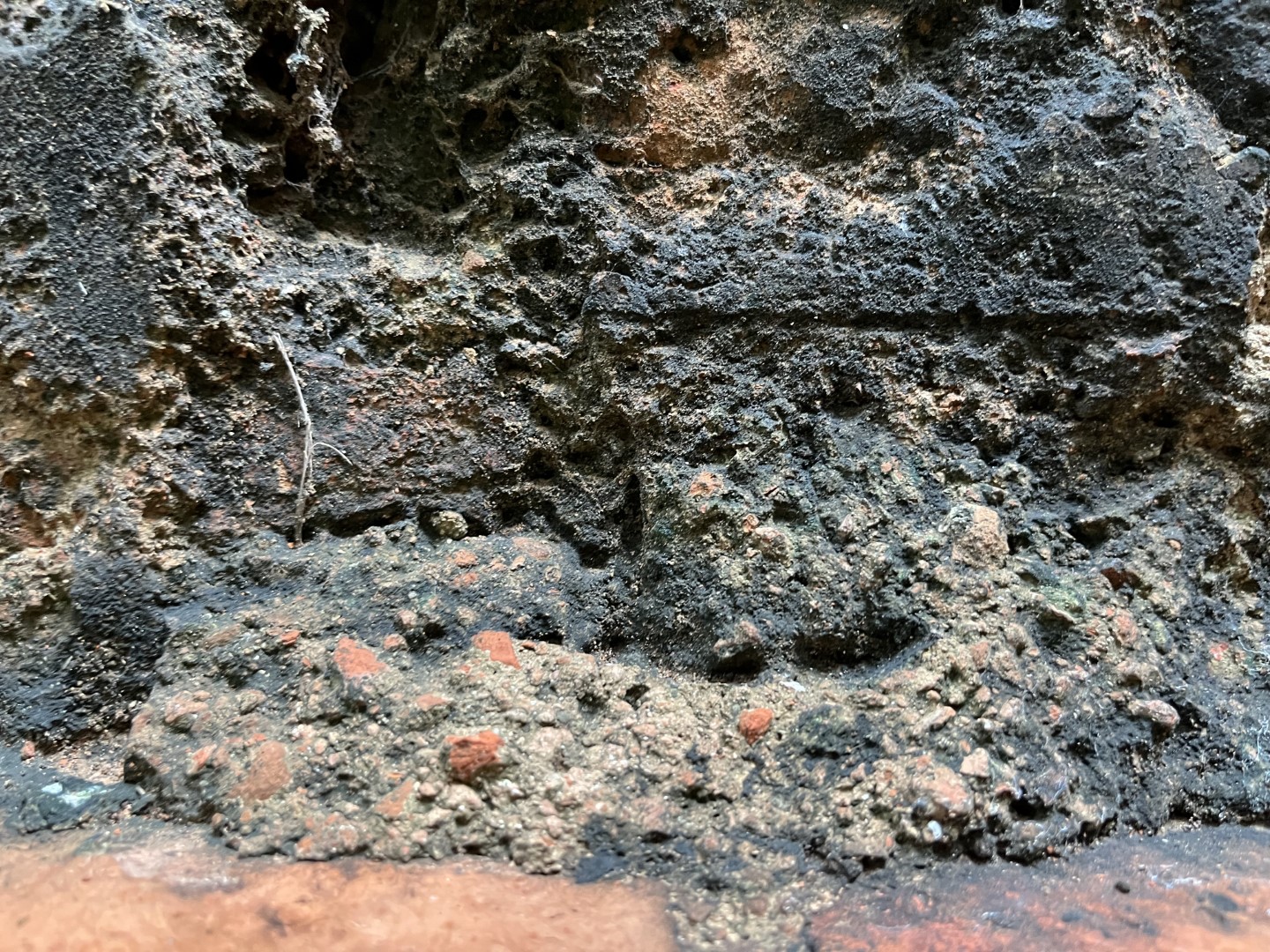

Supplement: Supplementary file 1 [file mmc1.zip › Demo_Historic_Place_Dataset/Fungus/IMG_3670.JPG]

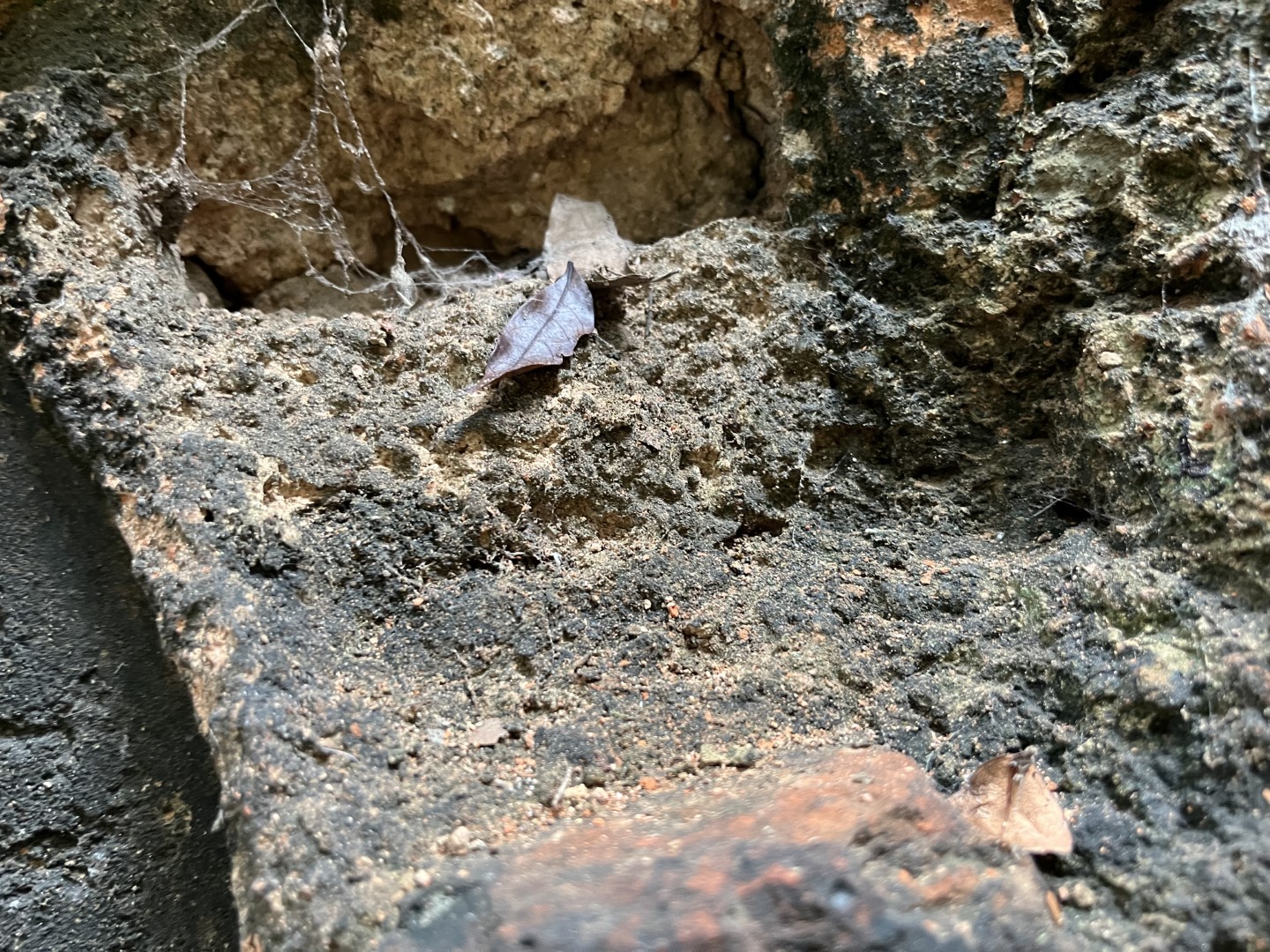

Supplement: Supplementary file 1 [file mmc1.zip › Demo_Historic_Place_Dataset/Fungus/IMG_3671.JPG]

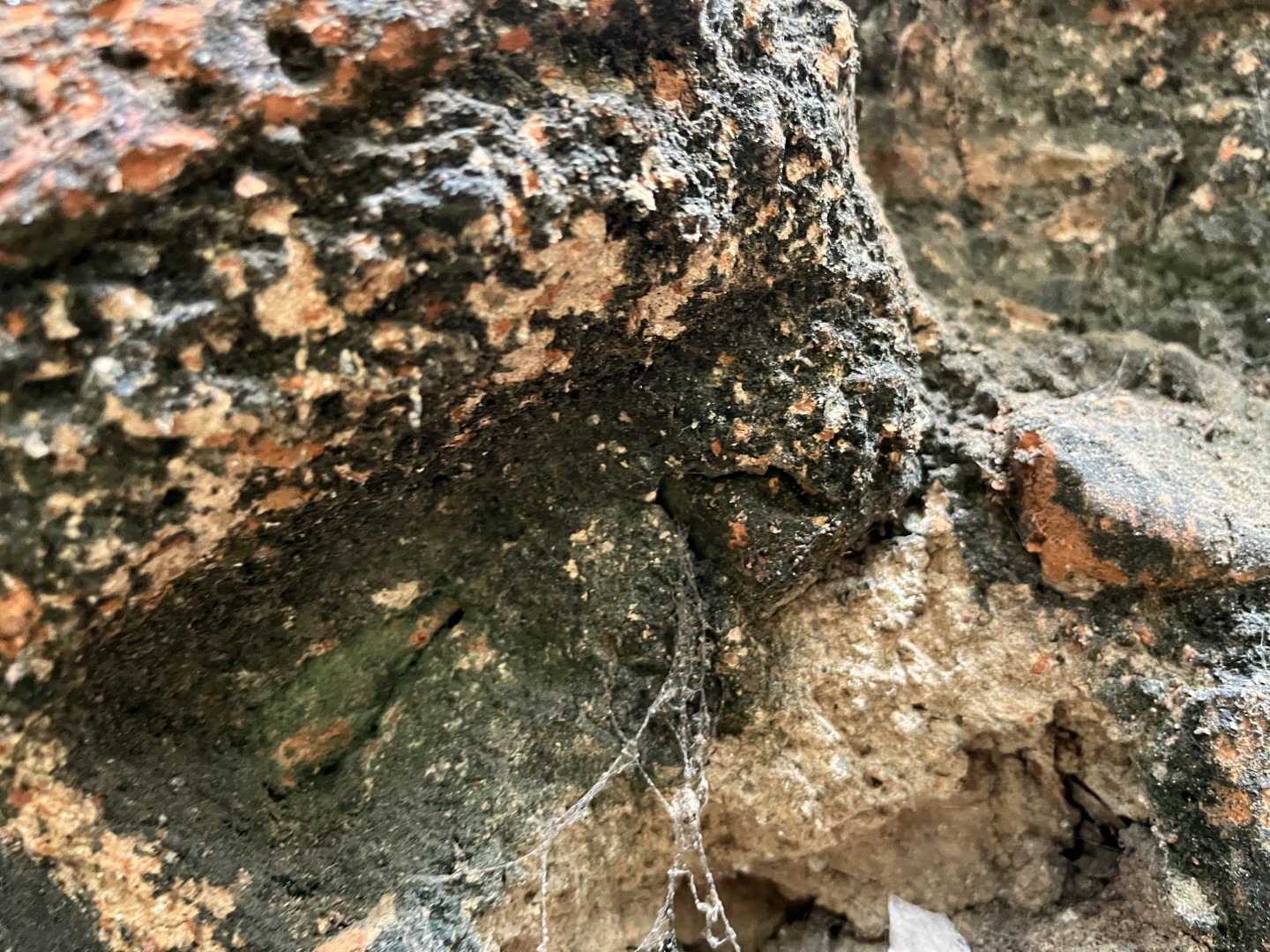

Supplement: Supplementary file 1 [file mmc1.zip › Demo_Historic_Place_Dataset/Fungus/IMG_3672.JPG]

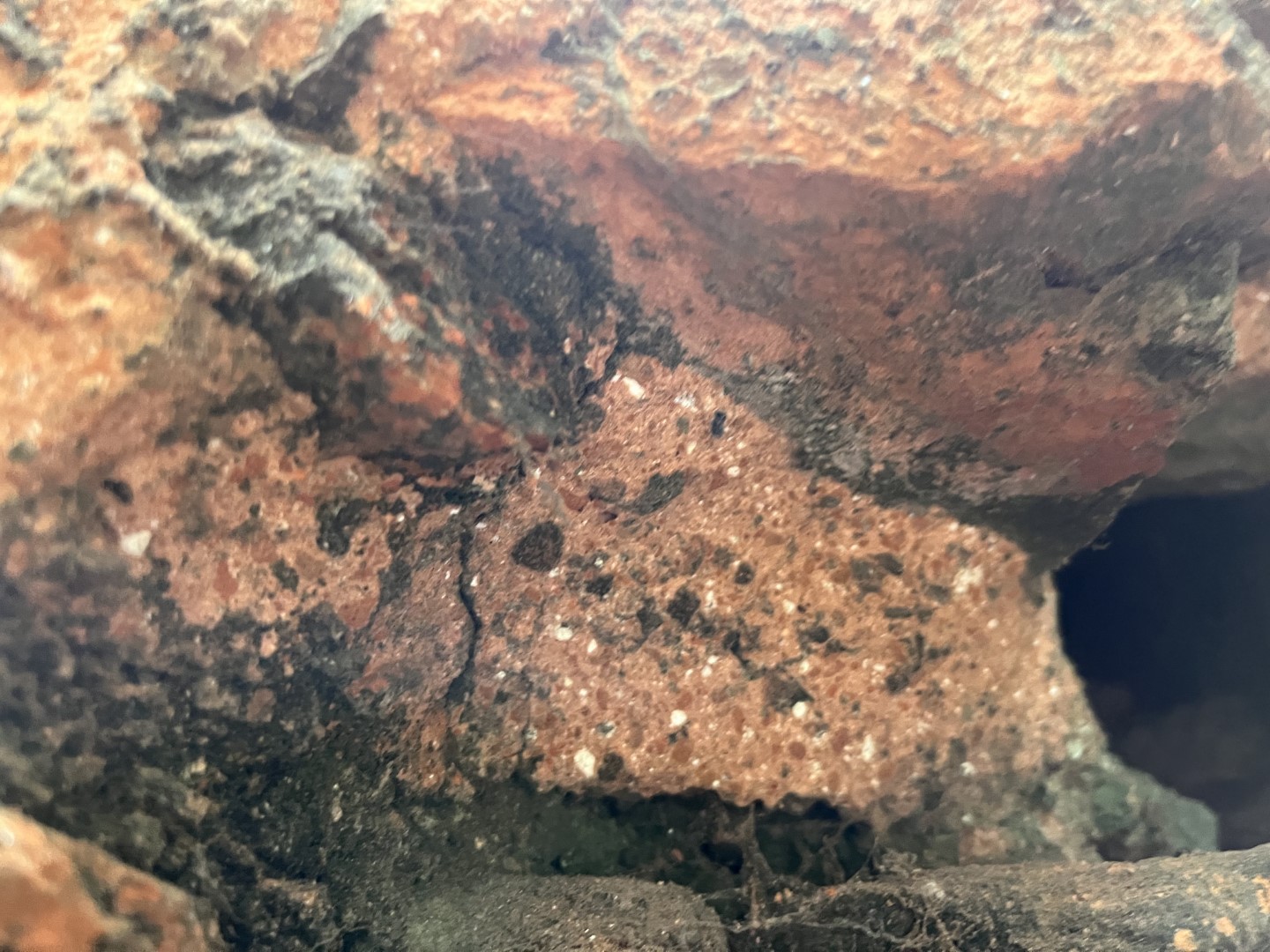

Supplement: Supplementary file 1 [file mmc1.zip › Demo_Historic_Place_Dataset/Fungus/IMG_3673.JPG]

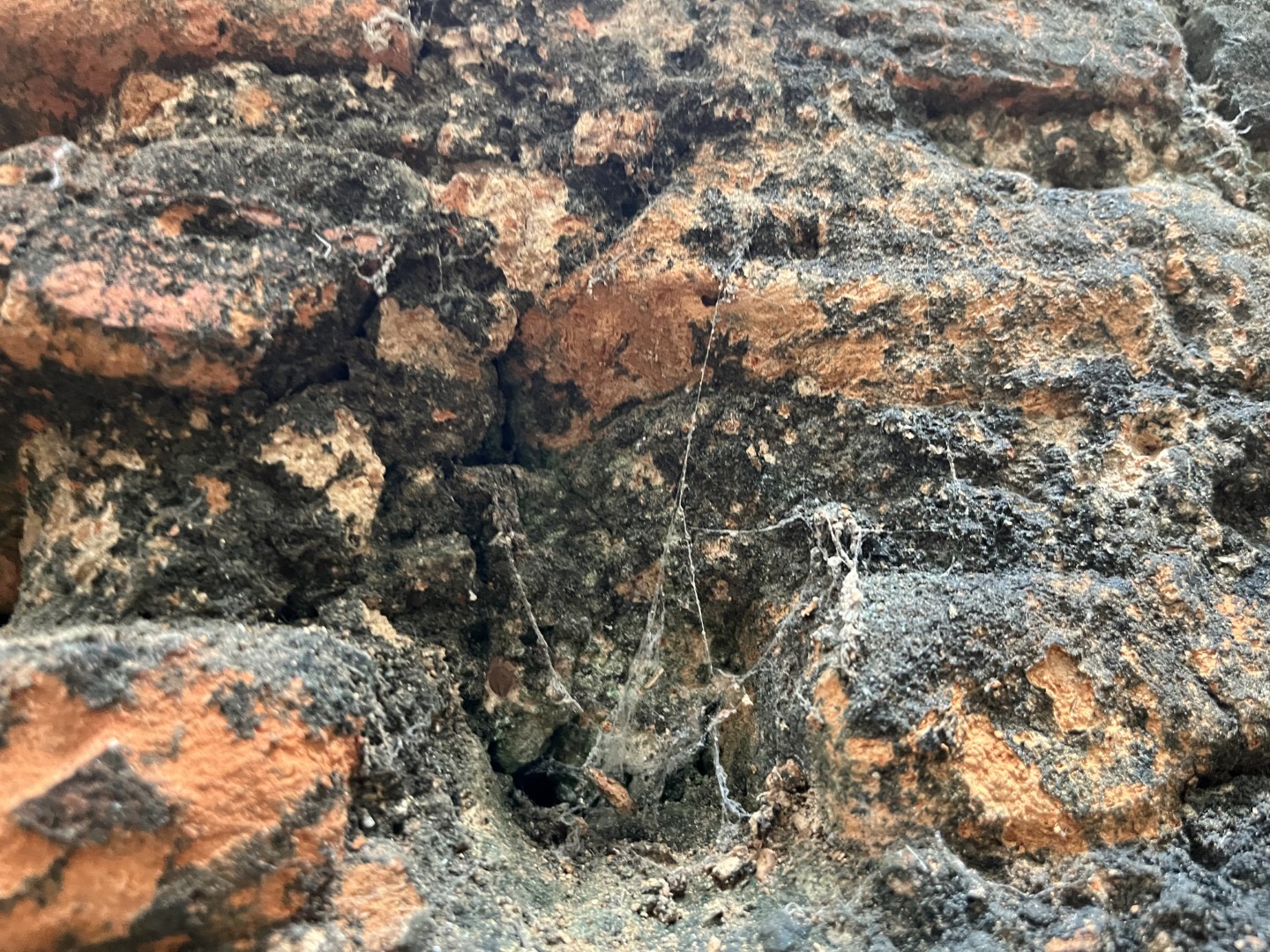

Supplement: Supplementary file 1 [file mmc1.zip › Demo_Historic_Place_Dataset/Fungus/IMG_3674.JPG]

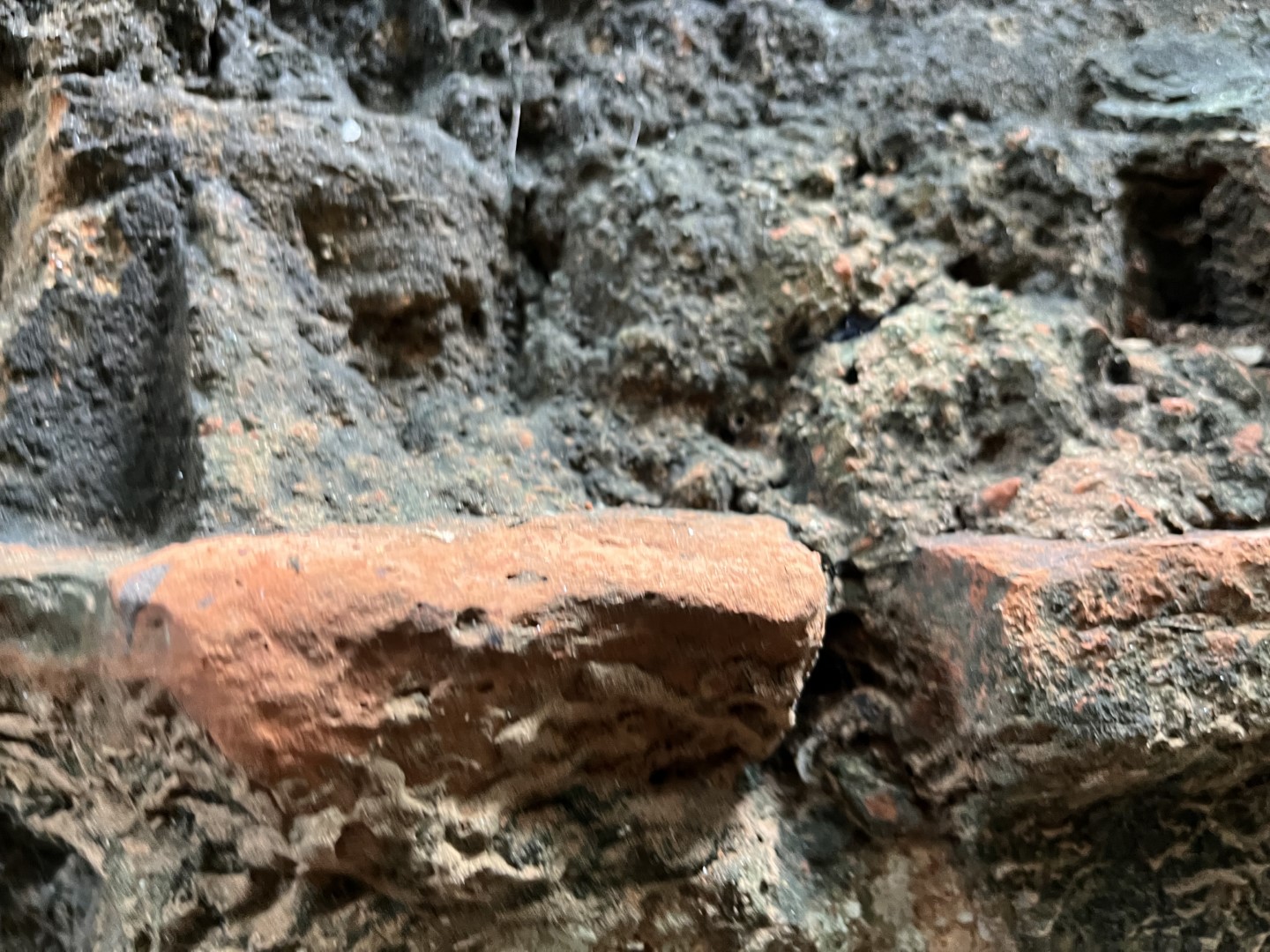

Supplement: Supplementary file 1 [file mmc1.zip › Demo_Historic_Place_Dataset/Fungus/IMG_3675.JPG]

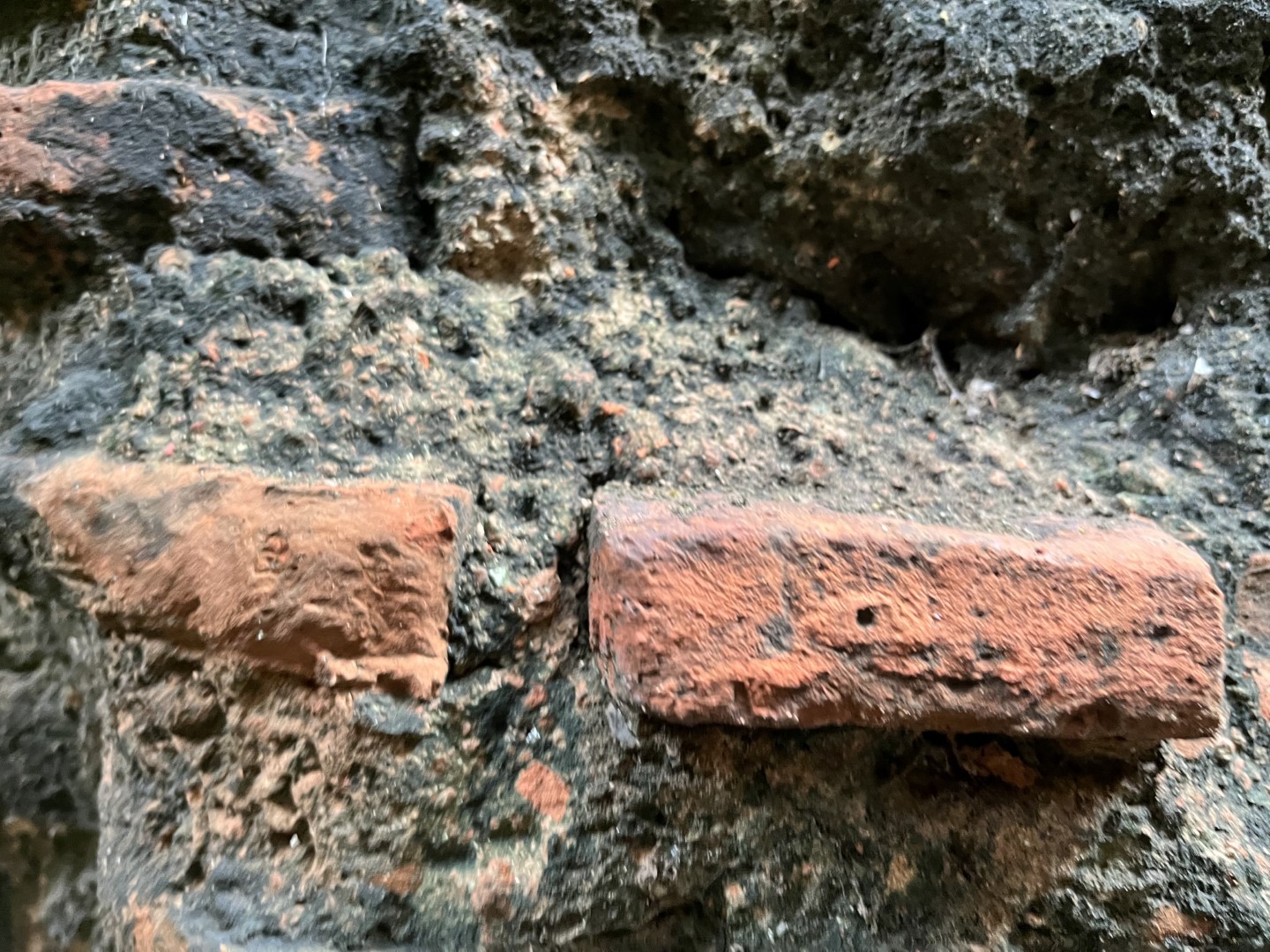

Supplement: Supplementary file 1 [file mmc1.zip › Demo_Historic_Place_Dataset/Fungus/IMG_3676.JPG]

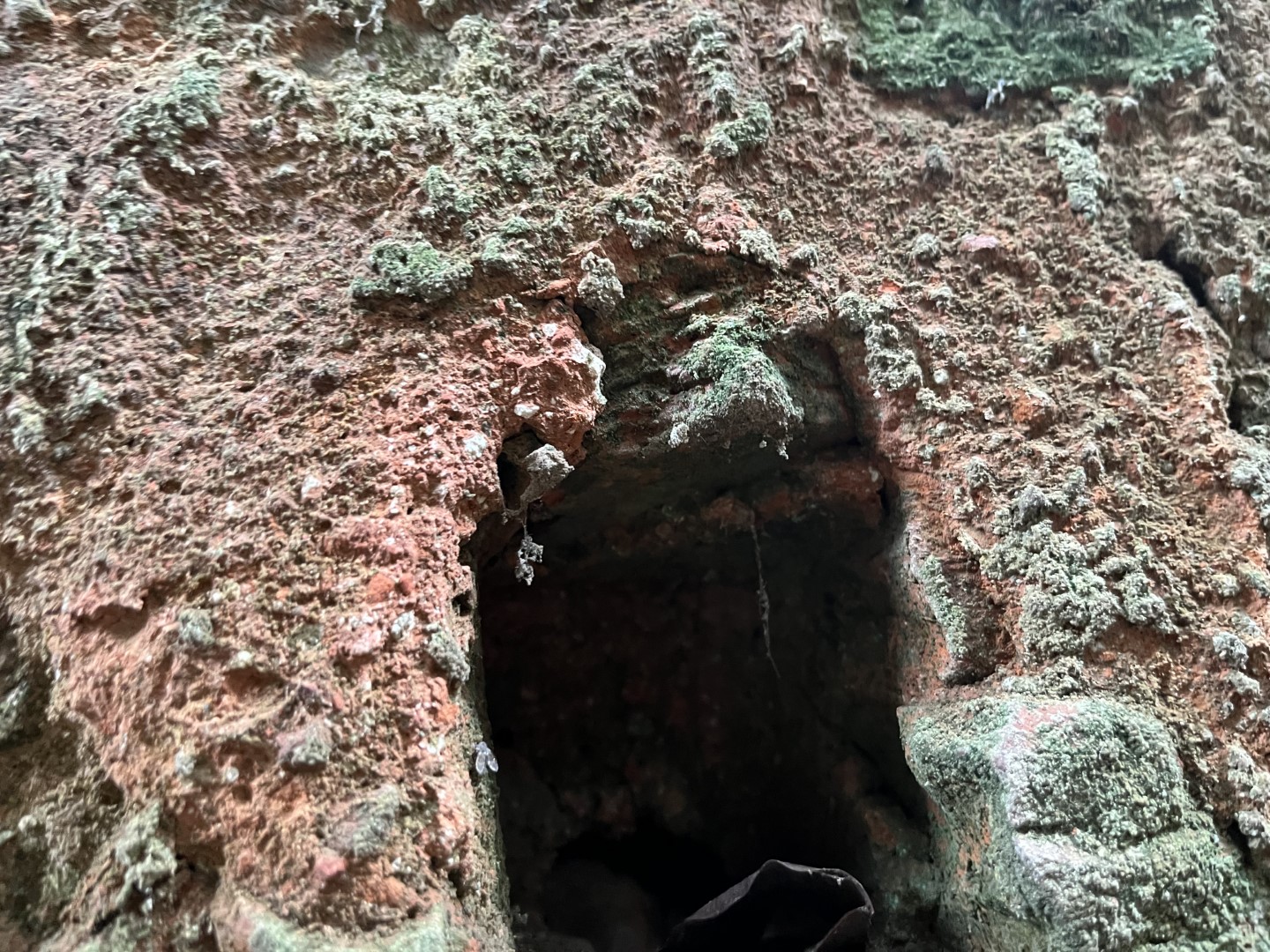

Supplement: Supplementary file 1 [file mmc1.zip › Demo_Historic_Place_Dataset/Fungus/IMG_3677.JPG]

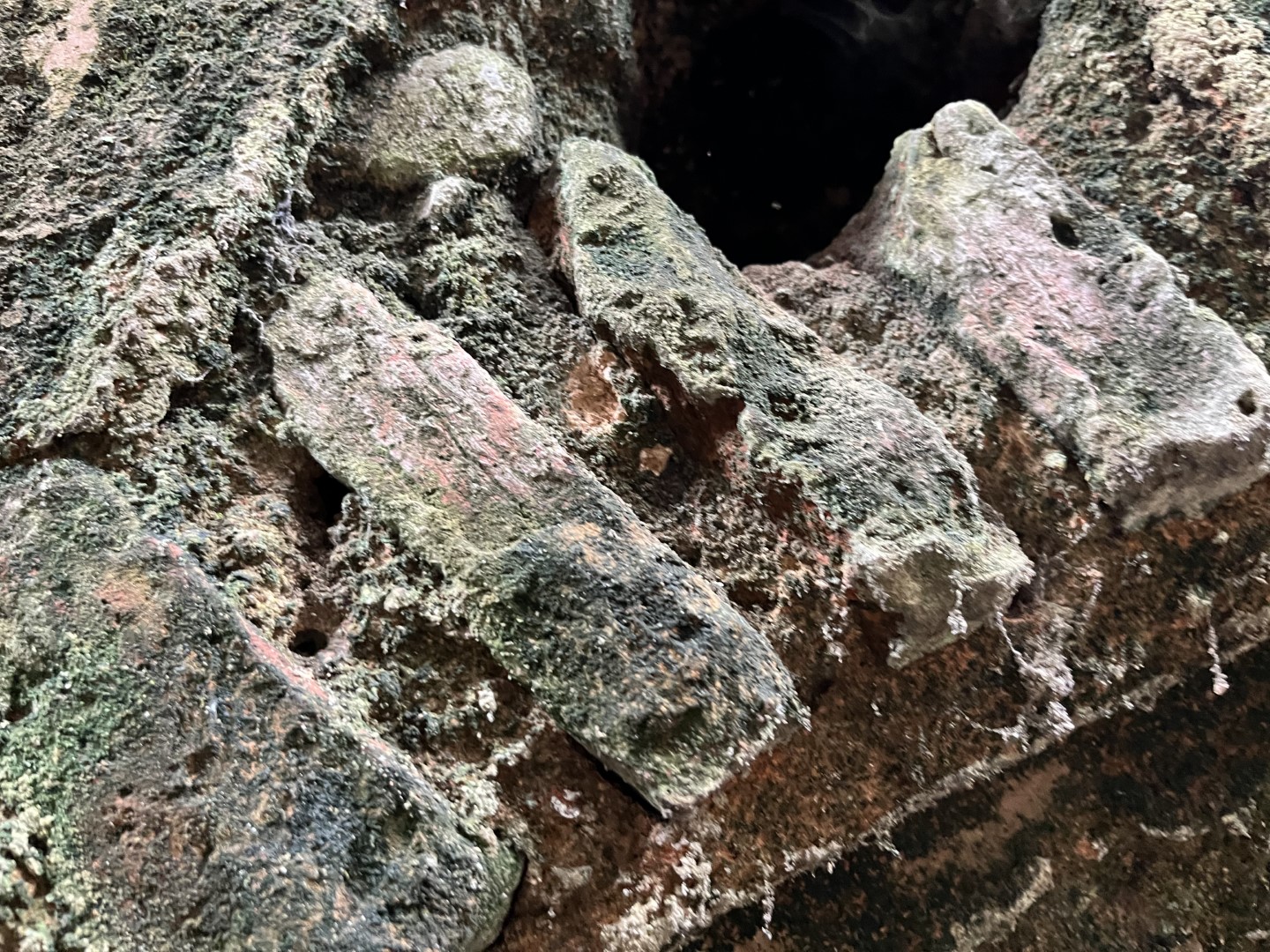

Supplement: Supplementary file 1 [file mmc1.zip › Demo_Historic_Place_Dataset/Fungus/IMG_3678.JPG]

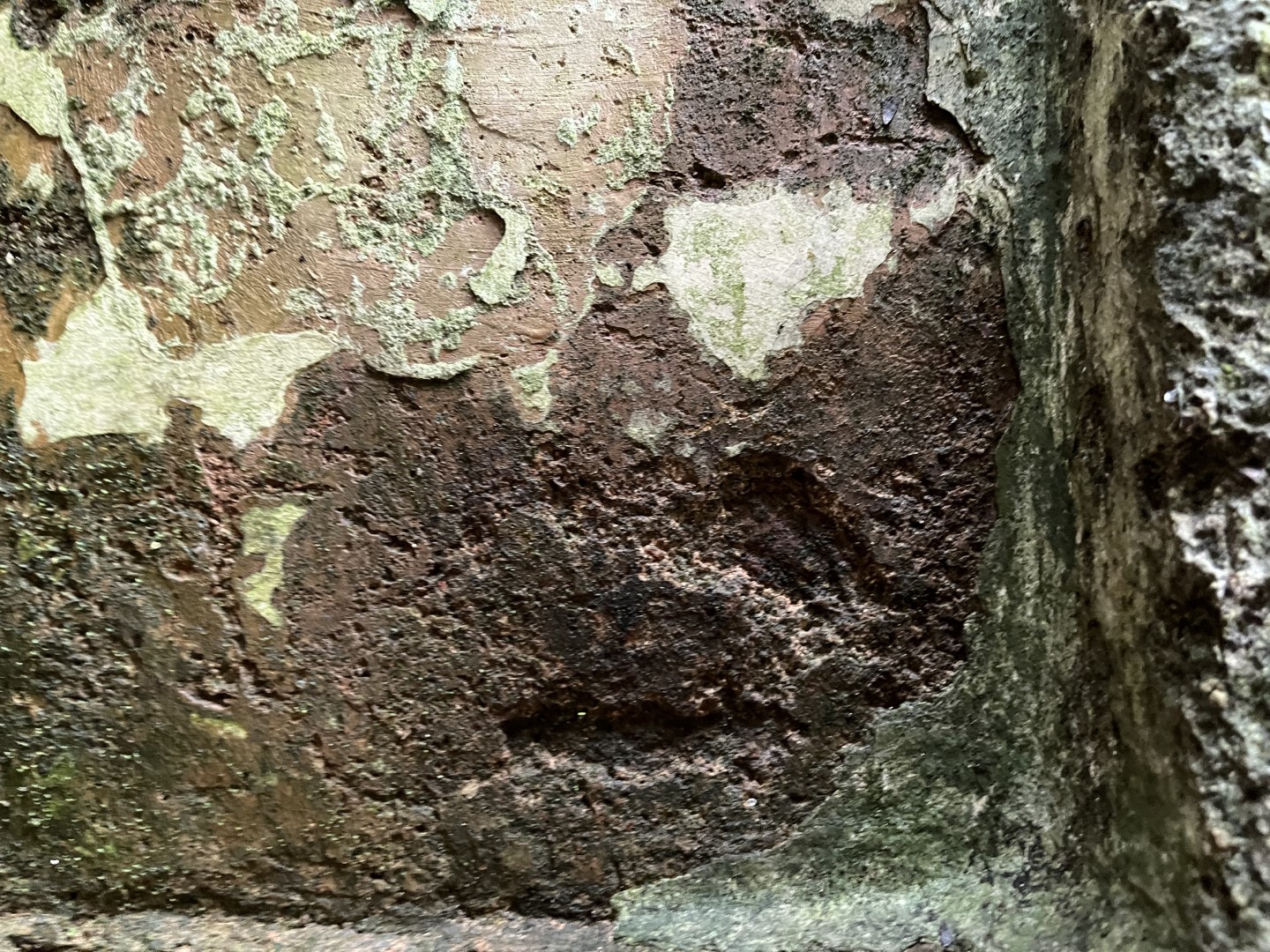

Supplement: Supplementary file 1 [file mmc1.zip › Demo_Historic_Place_Dataset/Fungus/IMG_3679.JPG]

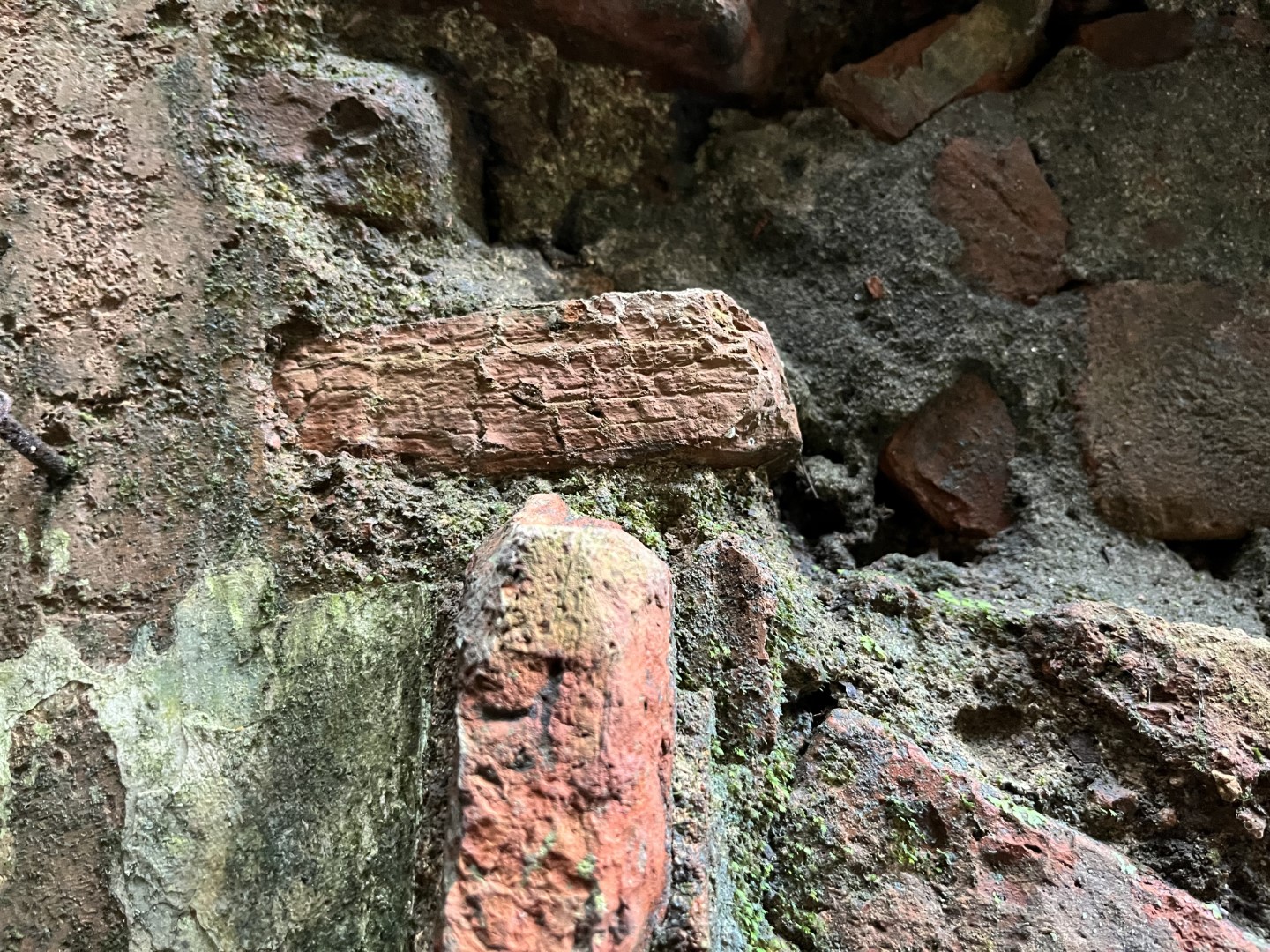

Supplement: Supplementary file 1 [file mmc1.zip › Demo_Historic_Place_Dataset/Fungus/IMG_3680.JPG]

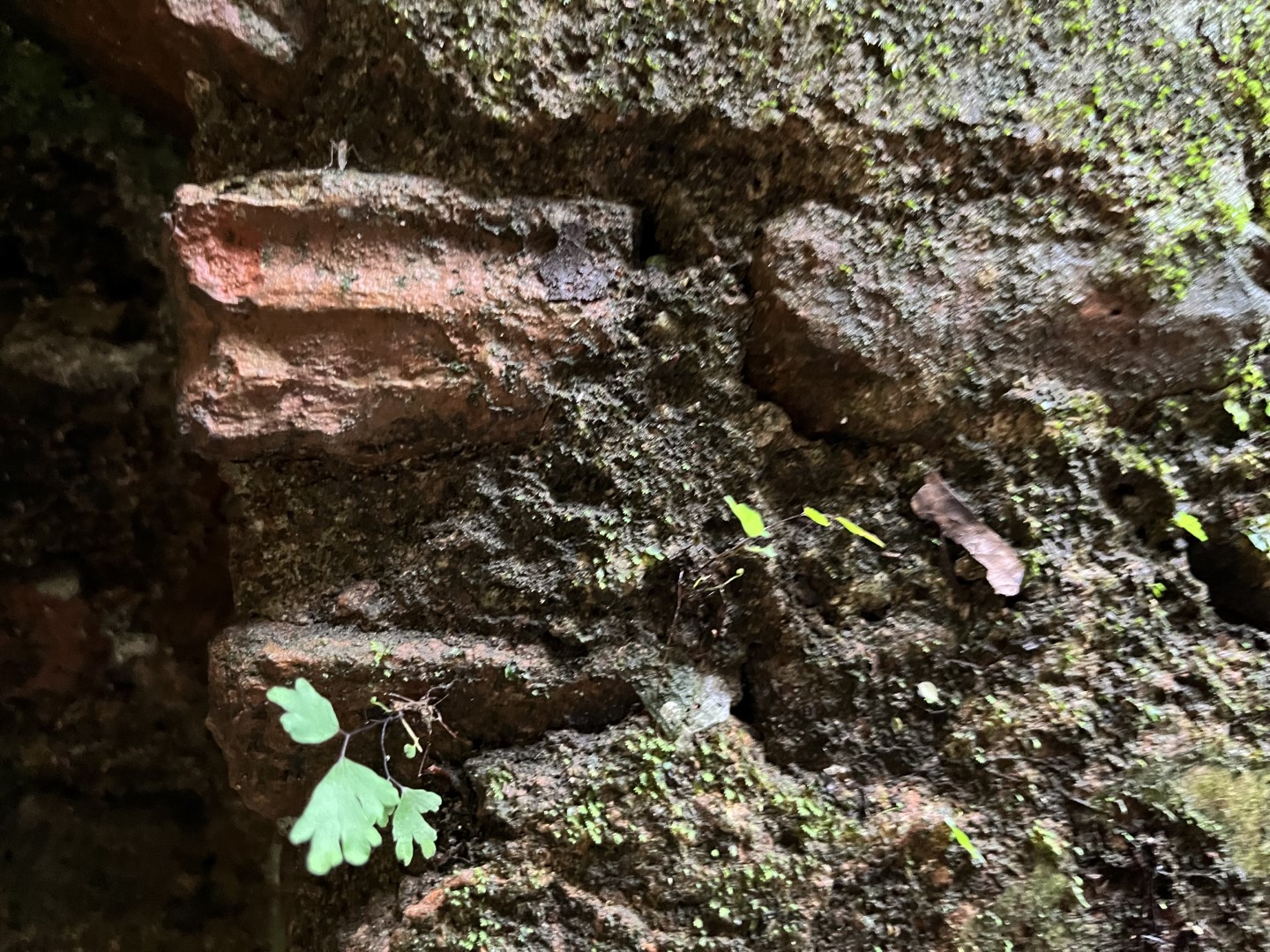

Supplement: Supplementary file 1 [file mmc1.zip › Demo_Historic_Place_Dataset/Fungus/IMG_3681.JPG]

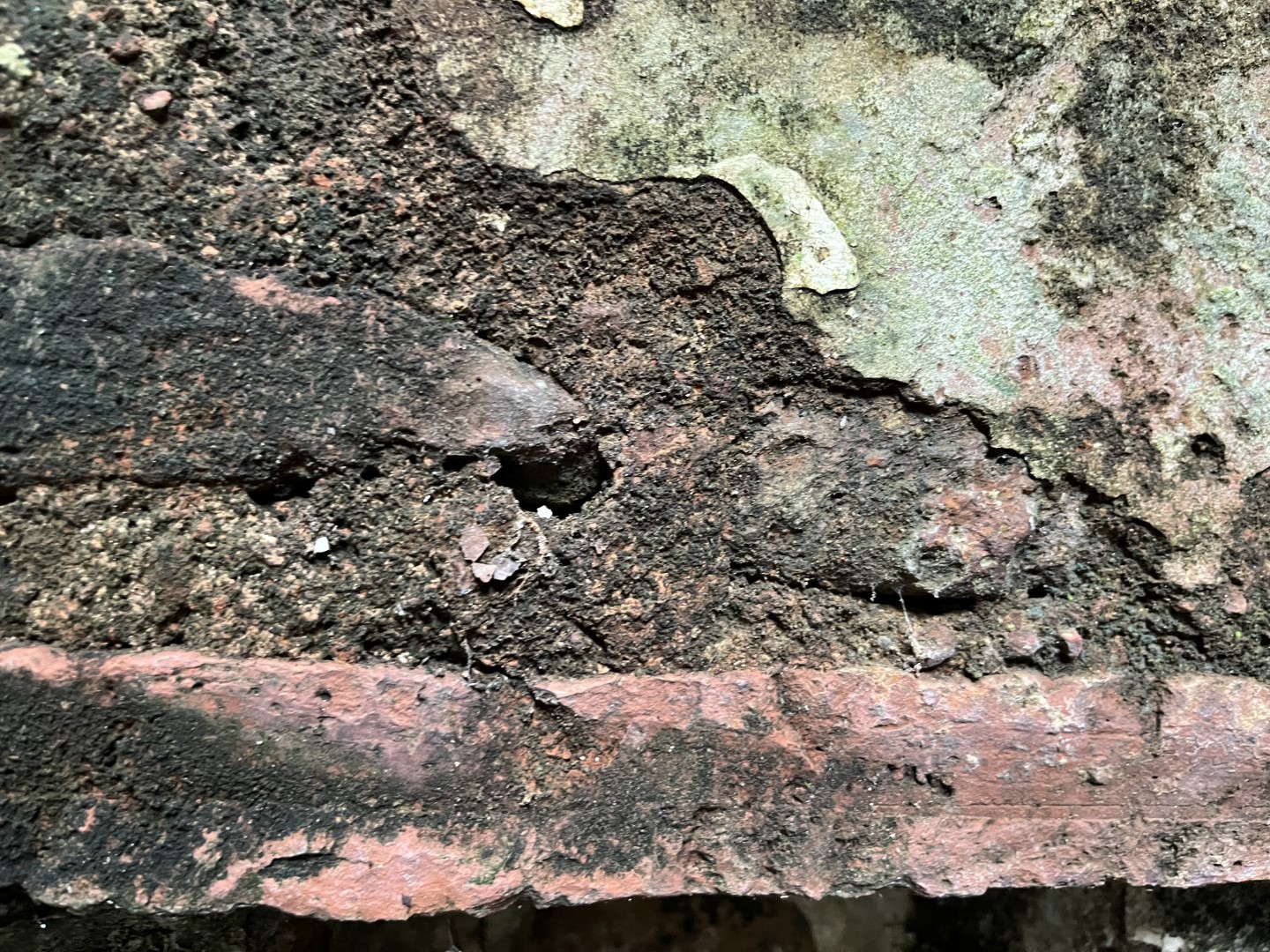

Supplement: Supplementary file 1 [file mmc1.zip › Demo_Historic_Place_Dataset/Fungus/IMG_3682.JPG]

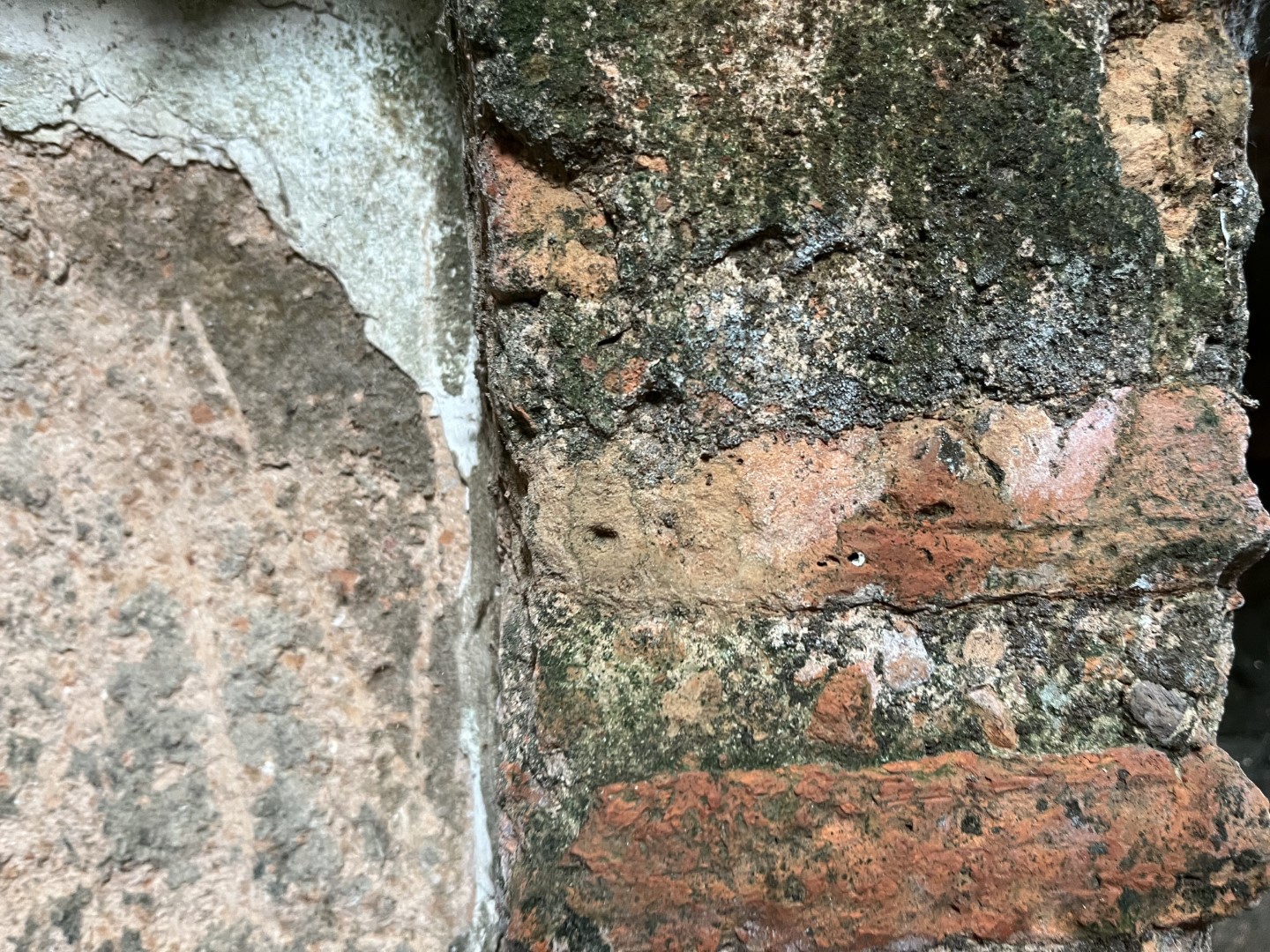

Supplement: Supplementary file 1 [file mmc1.zip › Demo_Historic_Place_Dataset/Fungus/IMG_3683.JPG]

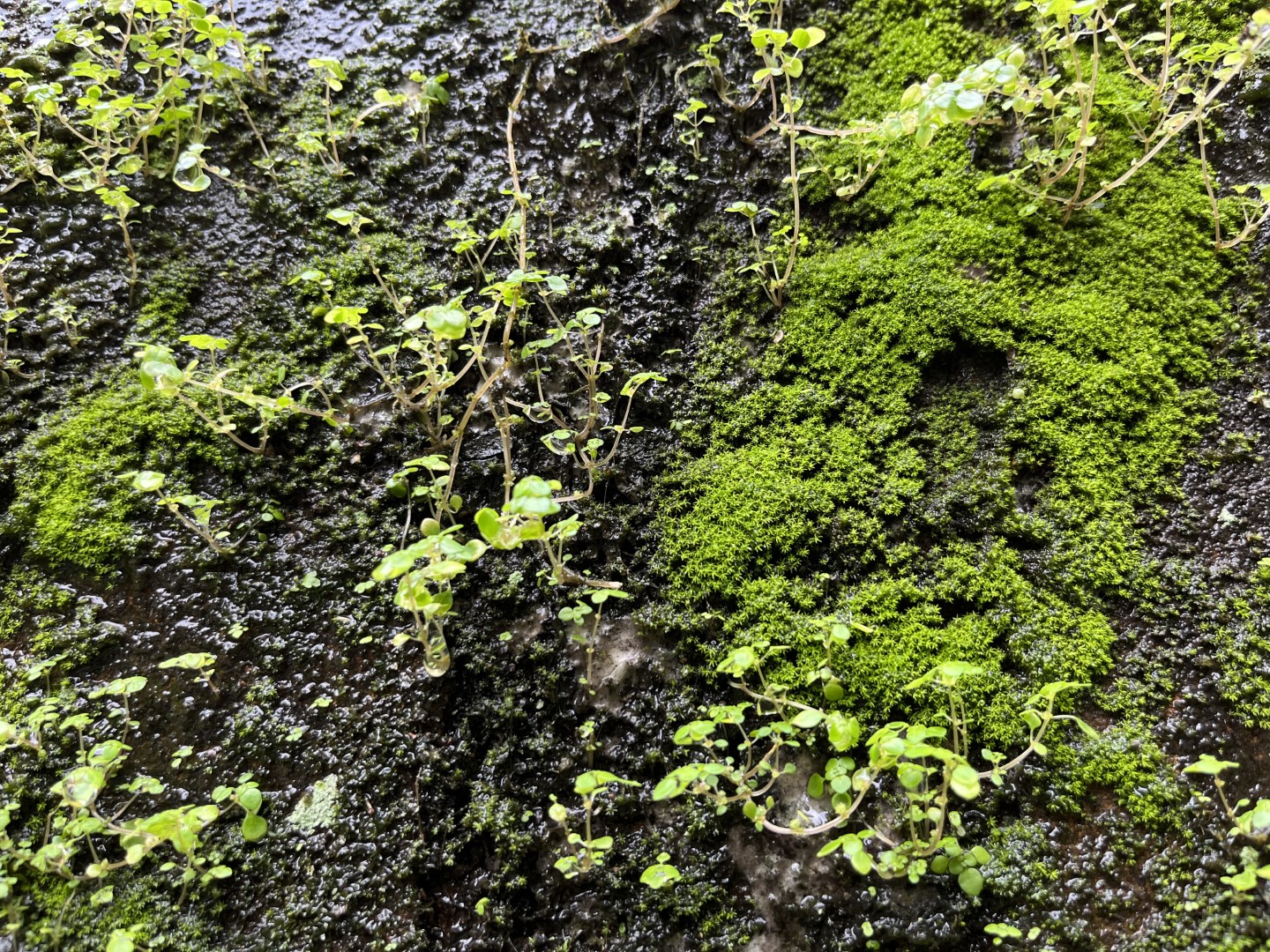

Supplement: Supplementary file 1 [file mmc1.zip › Demo_Historic_Place_Dataset/Living plant/IMG_3422.JPG]

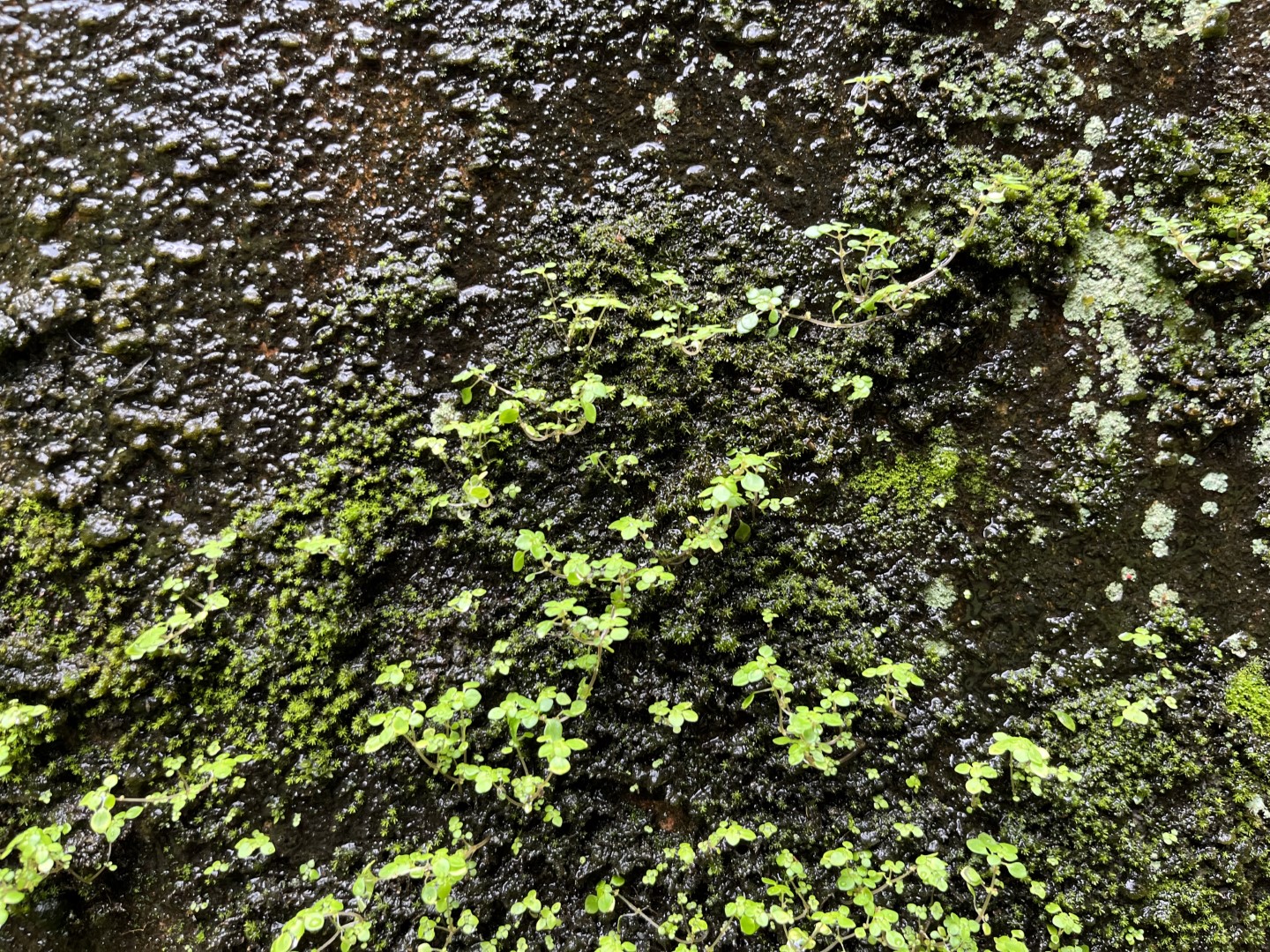

Supplement: Supplementary file 1 [file mmc1.zip › Demo_Historic_Place_Dataset/Living plant/IMG_3423.JPG]

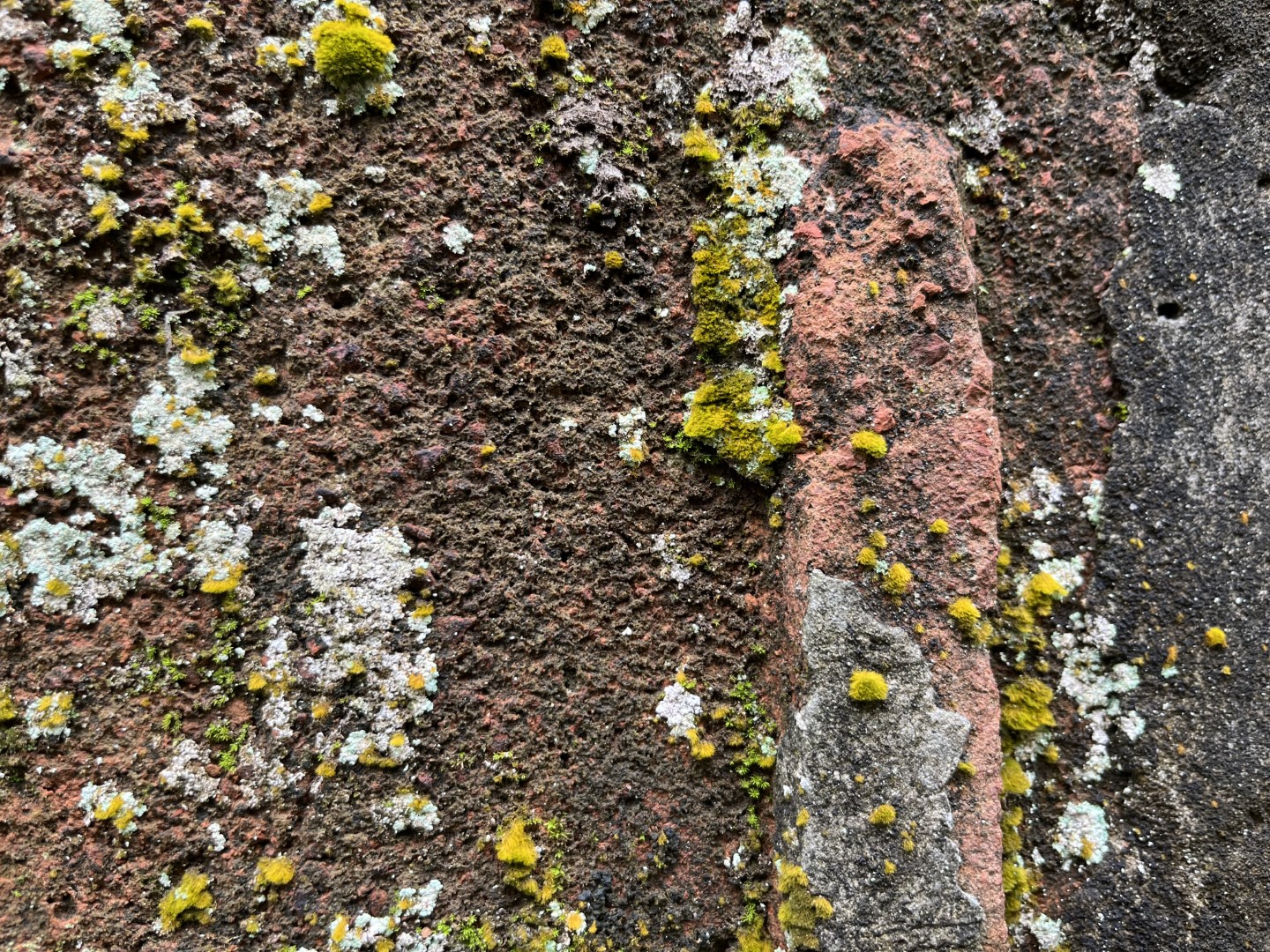

Supplement: Supplementary file 1 [file mmc1.zip › Demo_Historic_Place_Dataset/Living plant/IMG_3481.JPG]

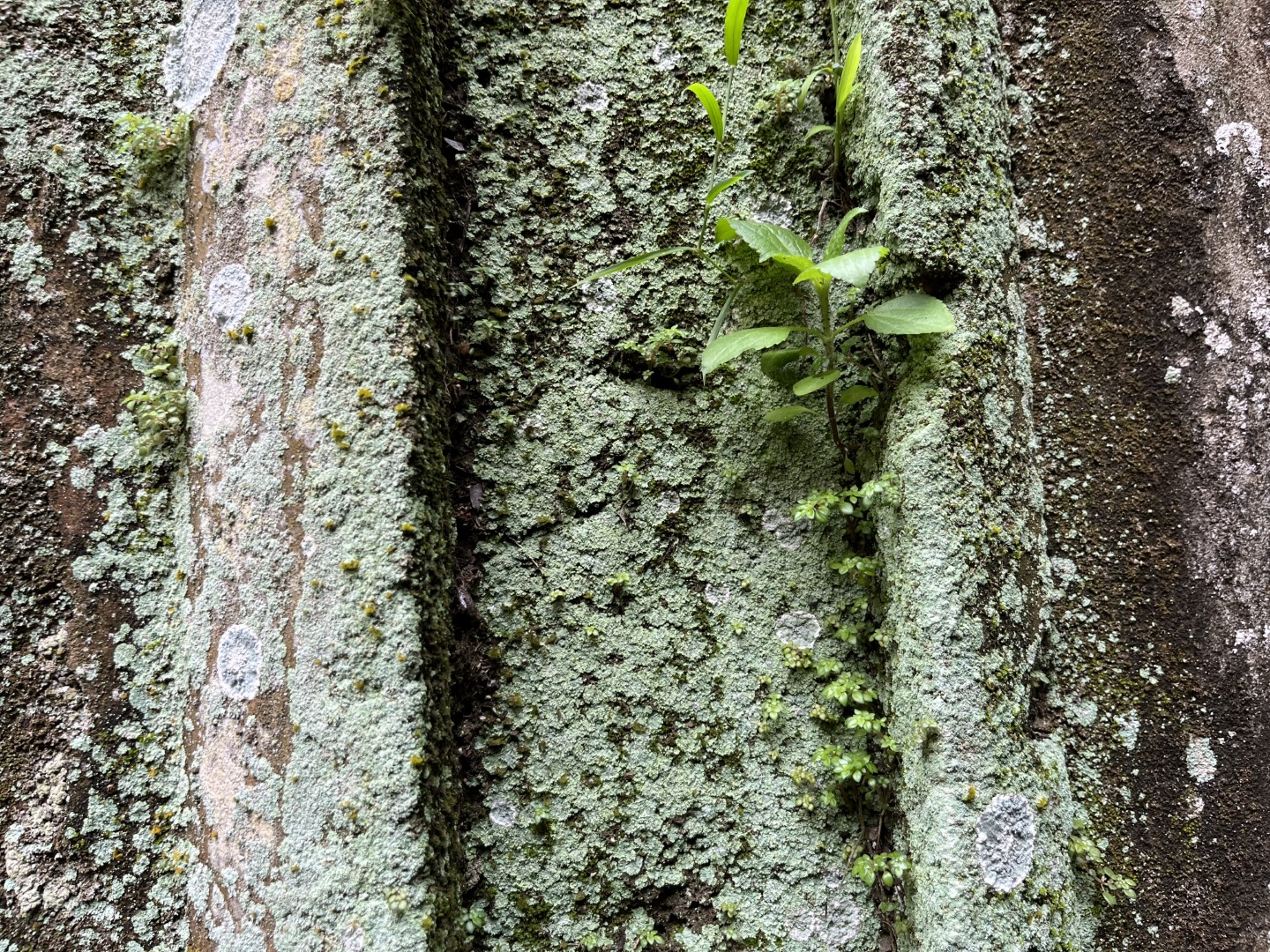

Supplement: Supplementary file 1 [file mmc1.zip › Demo_Historic_Place_Dataset/Living plant/IMG_3486.JPG]

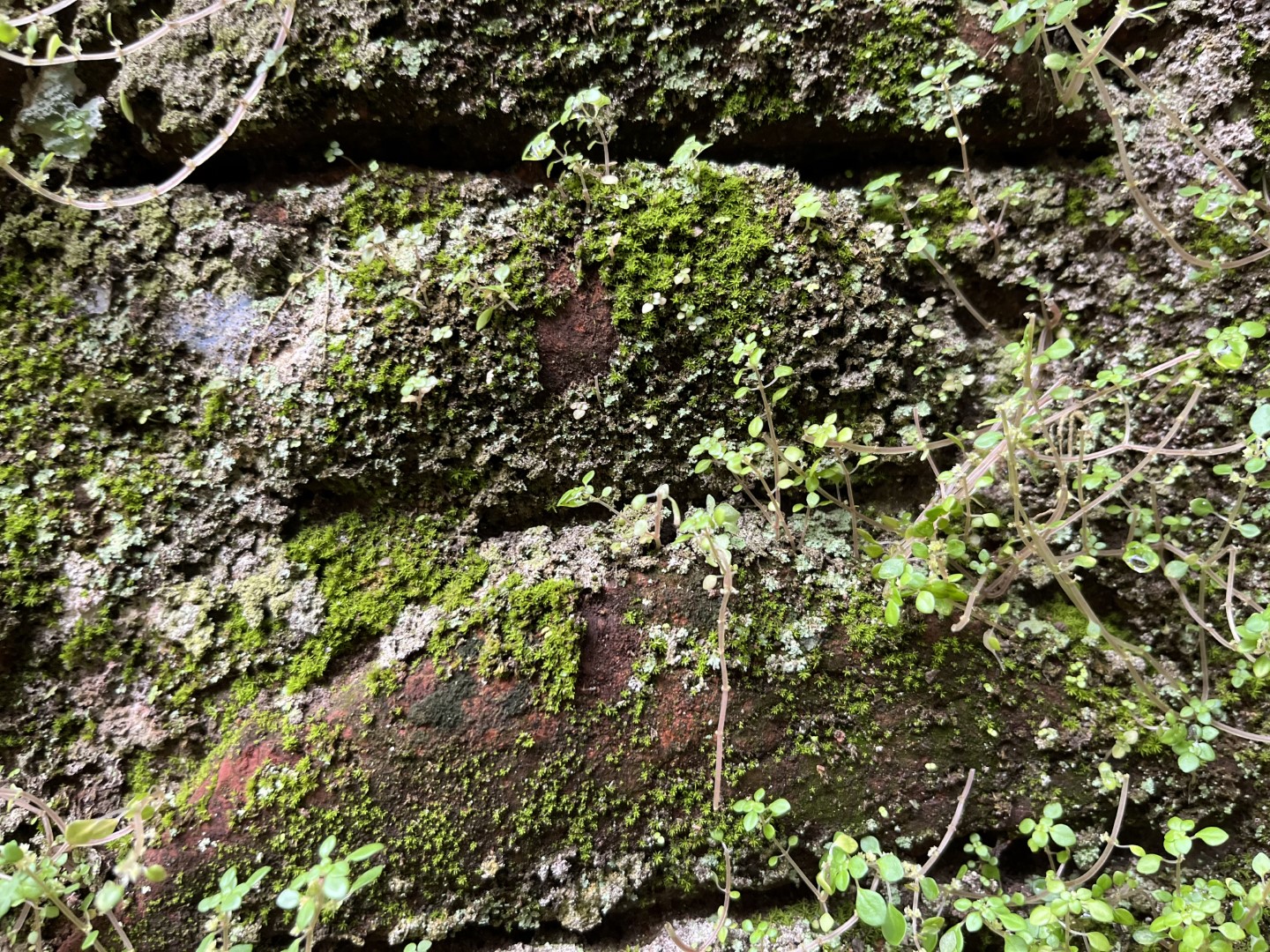

Supplement: Supplementary file 1 [file mmc1.zip › Demo_Historic_Place_Dataset/Living plant/IMG_3491.JPG]

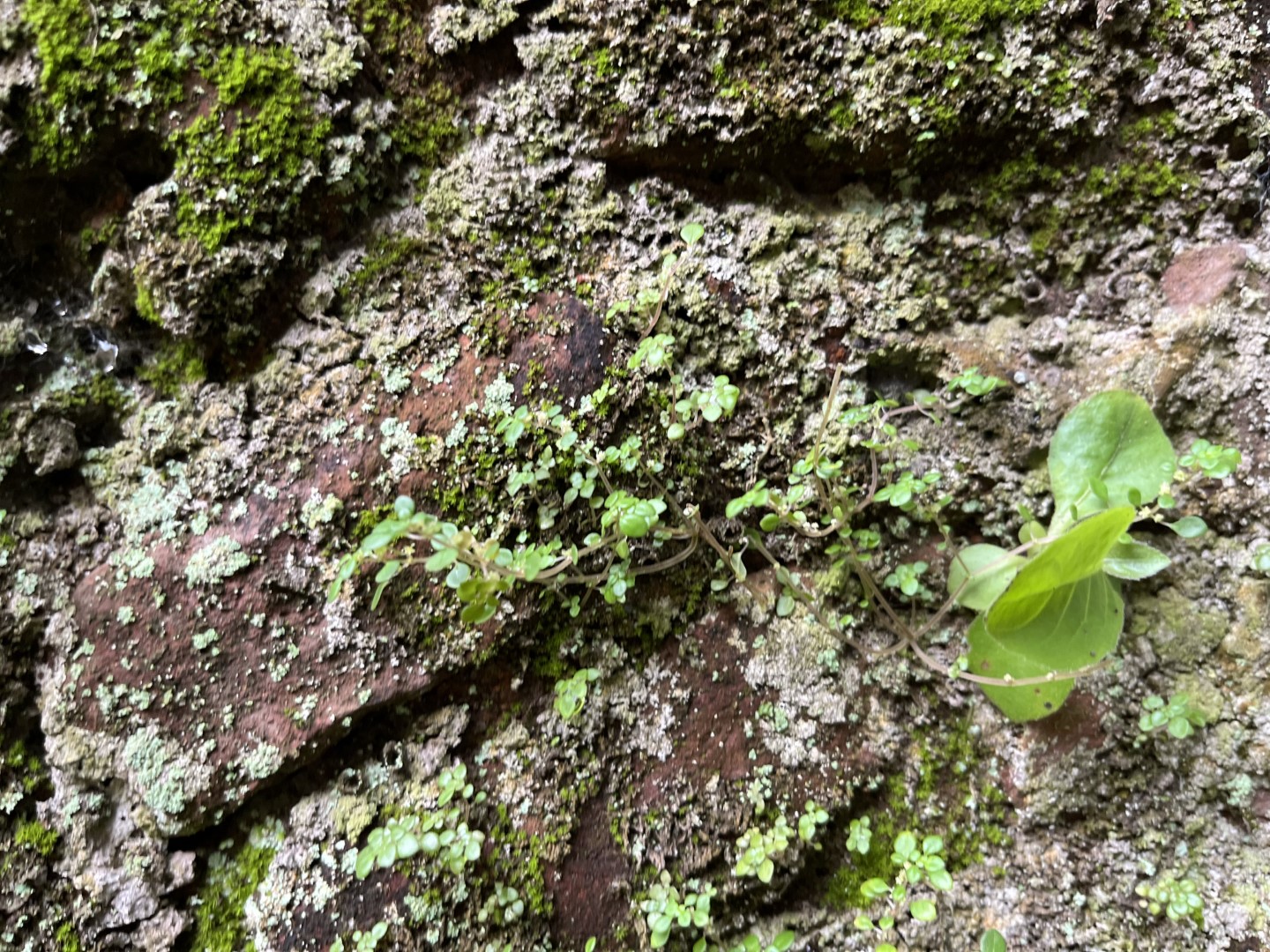

Supplement: Supplementary file 1 [file mmc1.zip › Demo_Historic_Place_Dataset/Living plant/IMG_3492.JPG]

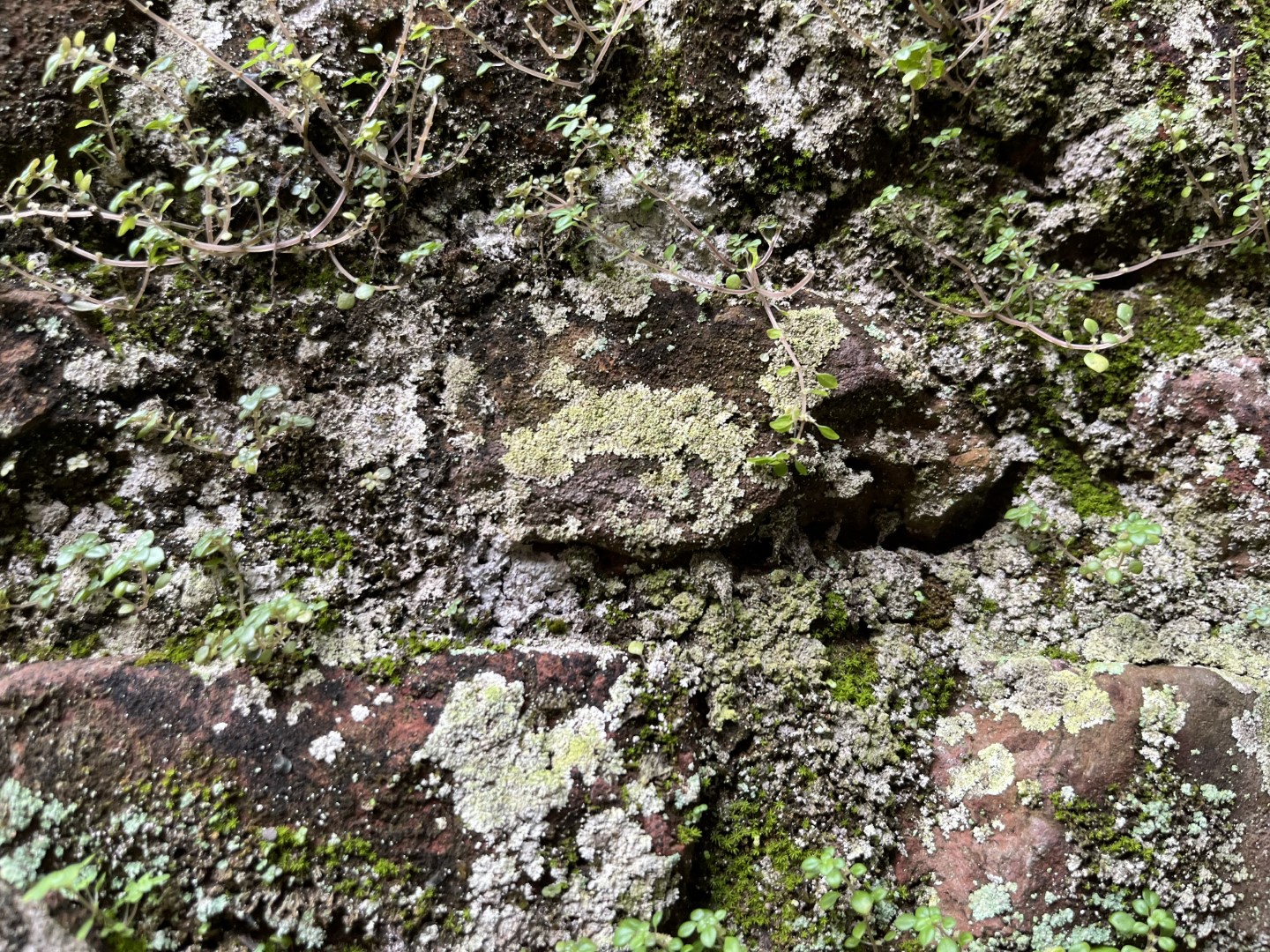

Supplement: Supplementary file 1 [file mmc1.zip › Demo_Historic_Place_Dataset/Living plant/IMG_3493.JPG]

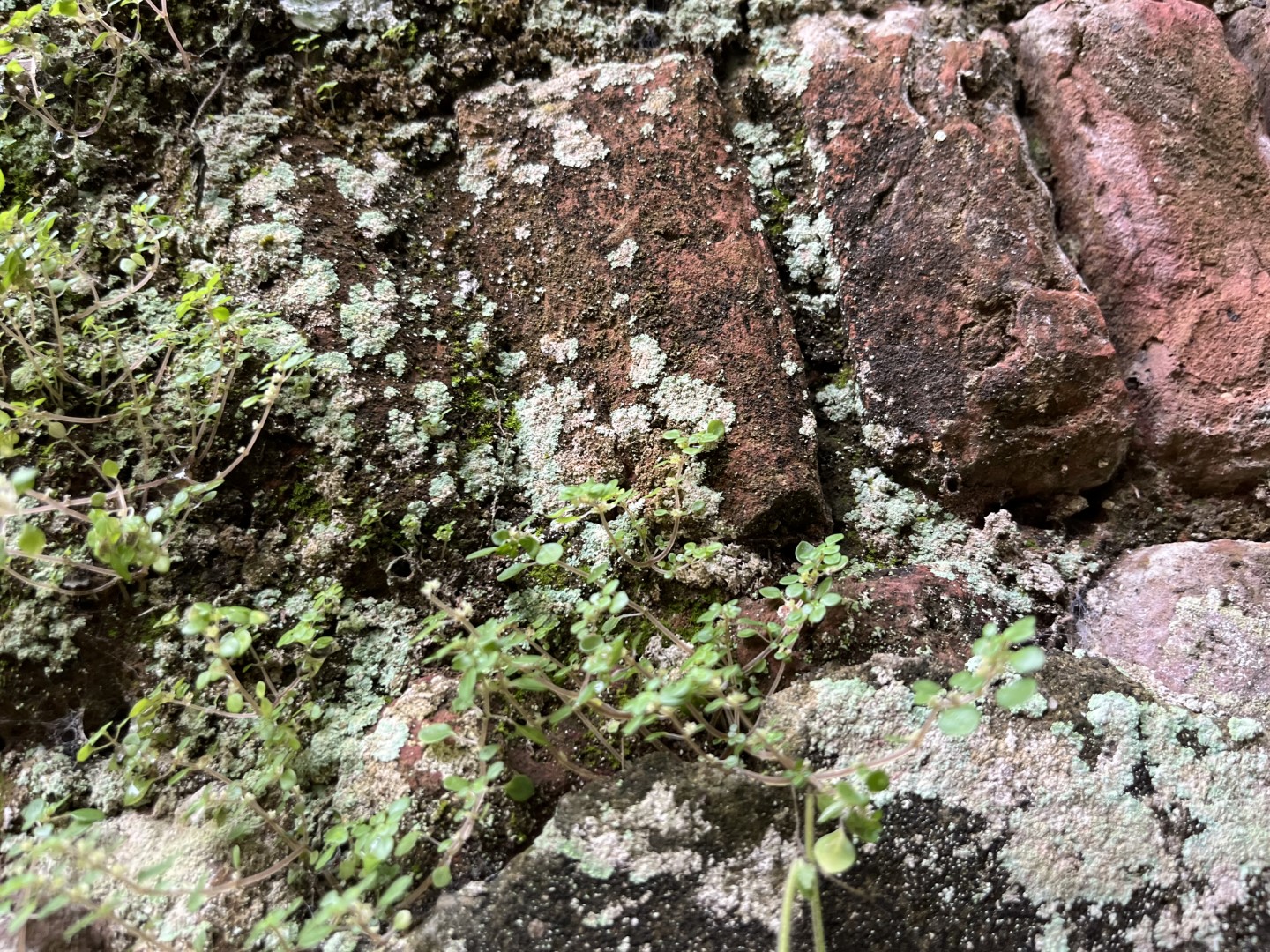

Supplement: Supplementary file 1 [file mmc1.zip › Demo_Historic_Place_Dataset/Living plant/IMG_3494.JPG]

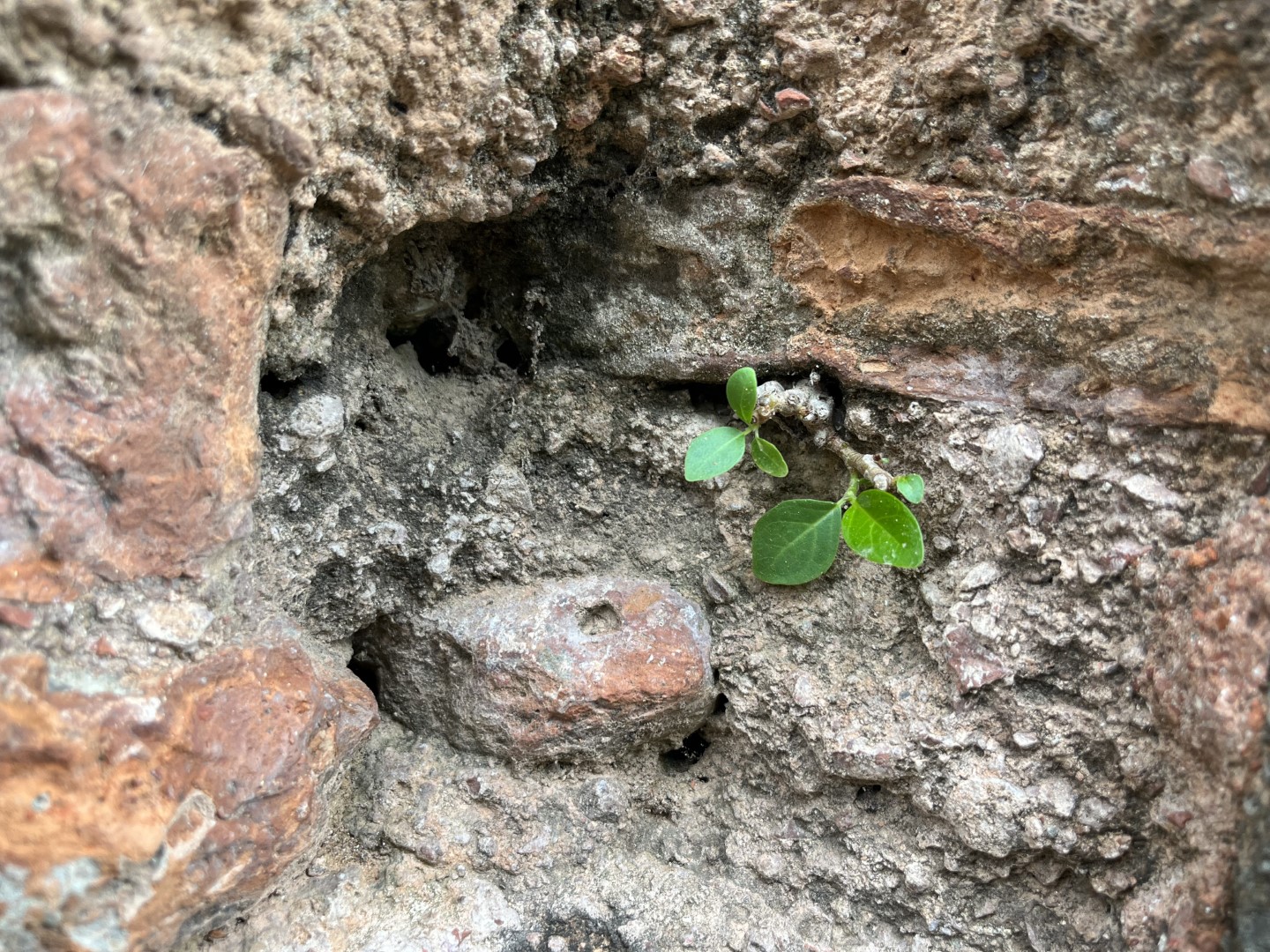

Supplement: Supplementary file 1 [file mmc1.zip › Demo_Historic_Place_Dataset/Living plant/IMG_3614.JPG]

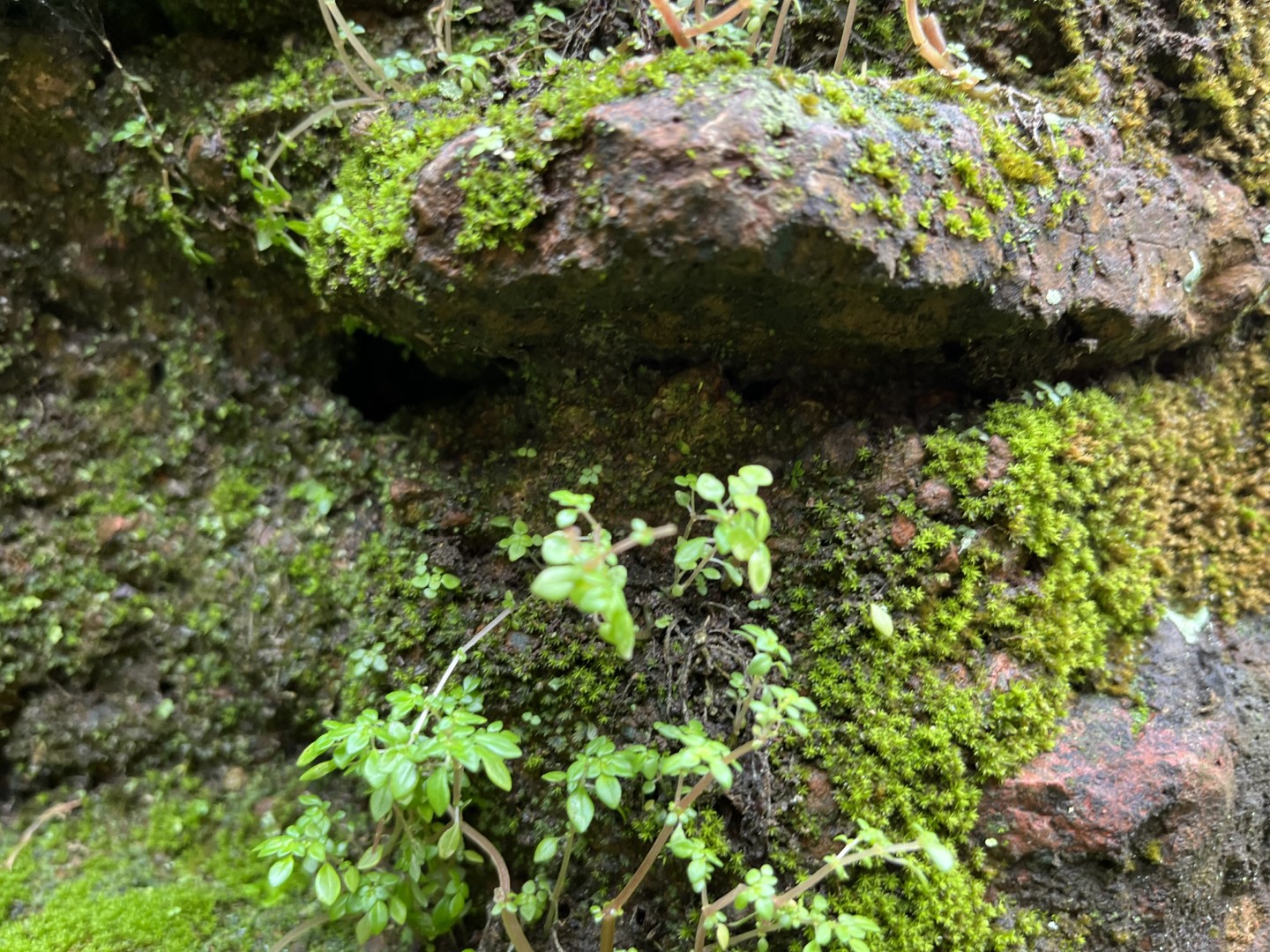

Supplement: Supplementary file 1 [file mmc1.zip › Demo_Historic_Place_Dataset/Living plant/IMG_3626.JPG]

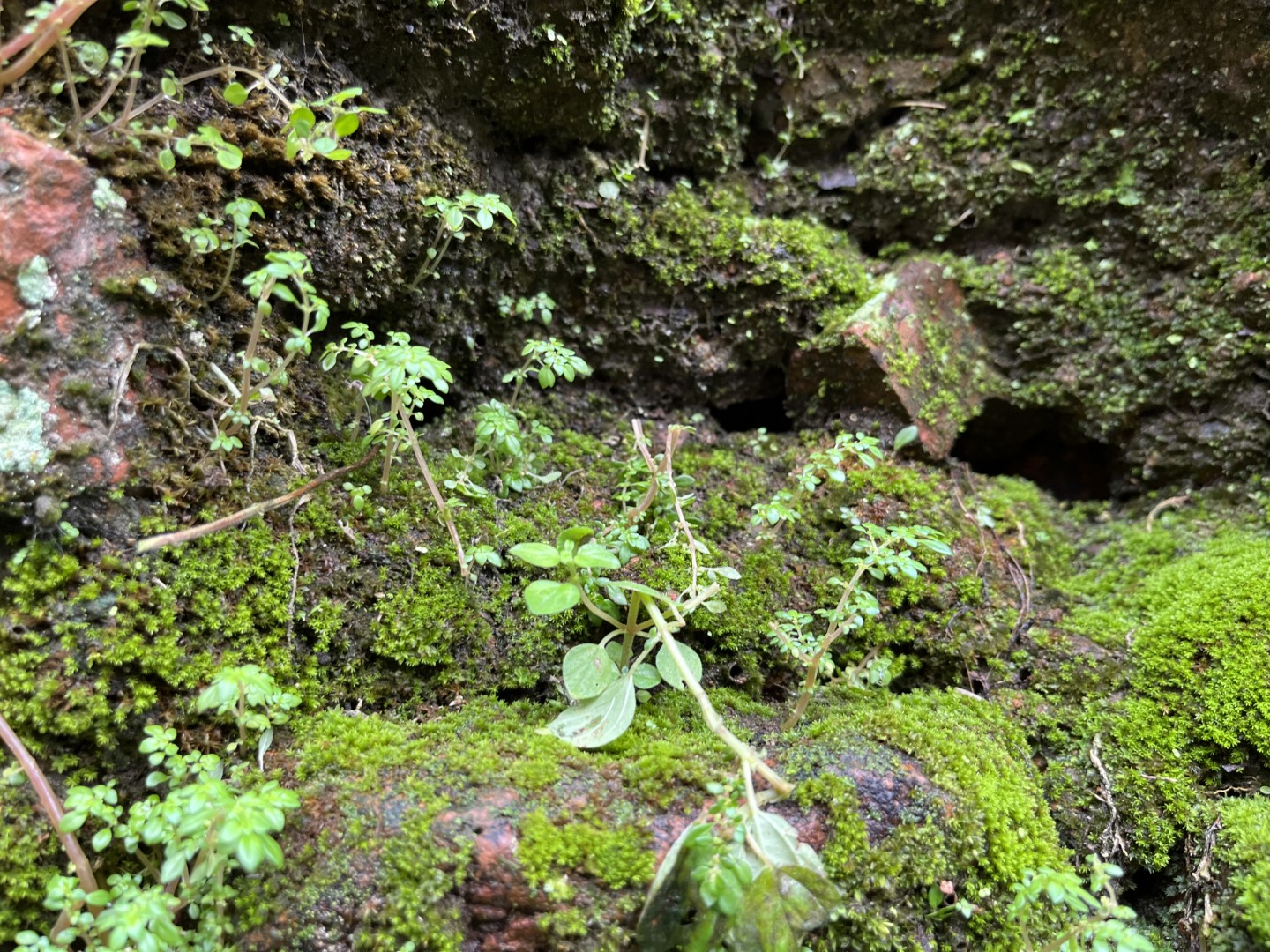

Supplement: Supplementary file 1 [file mmc1.zip › Demo_Historic_Place_Dataset/Living plant/IMG_3627.JPG]

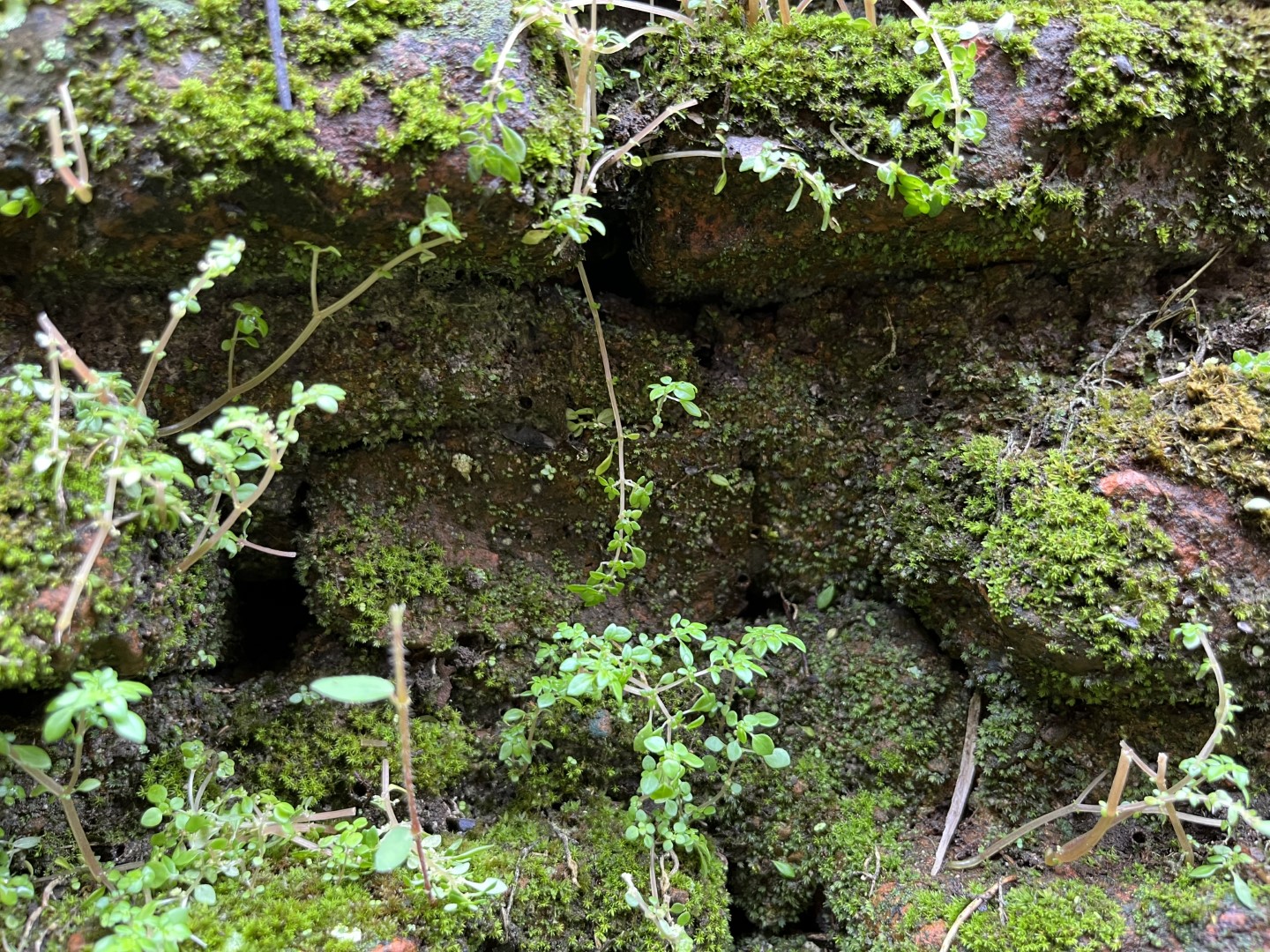

Supplement: Supplementary file 1 [file mmc1.zip › Demo_Historic_Place_Dataset/Living plant/IMG_3628.JPG]

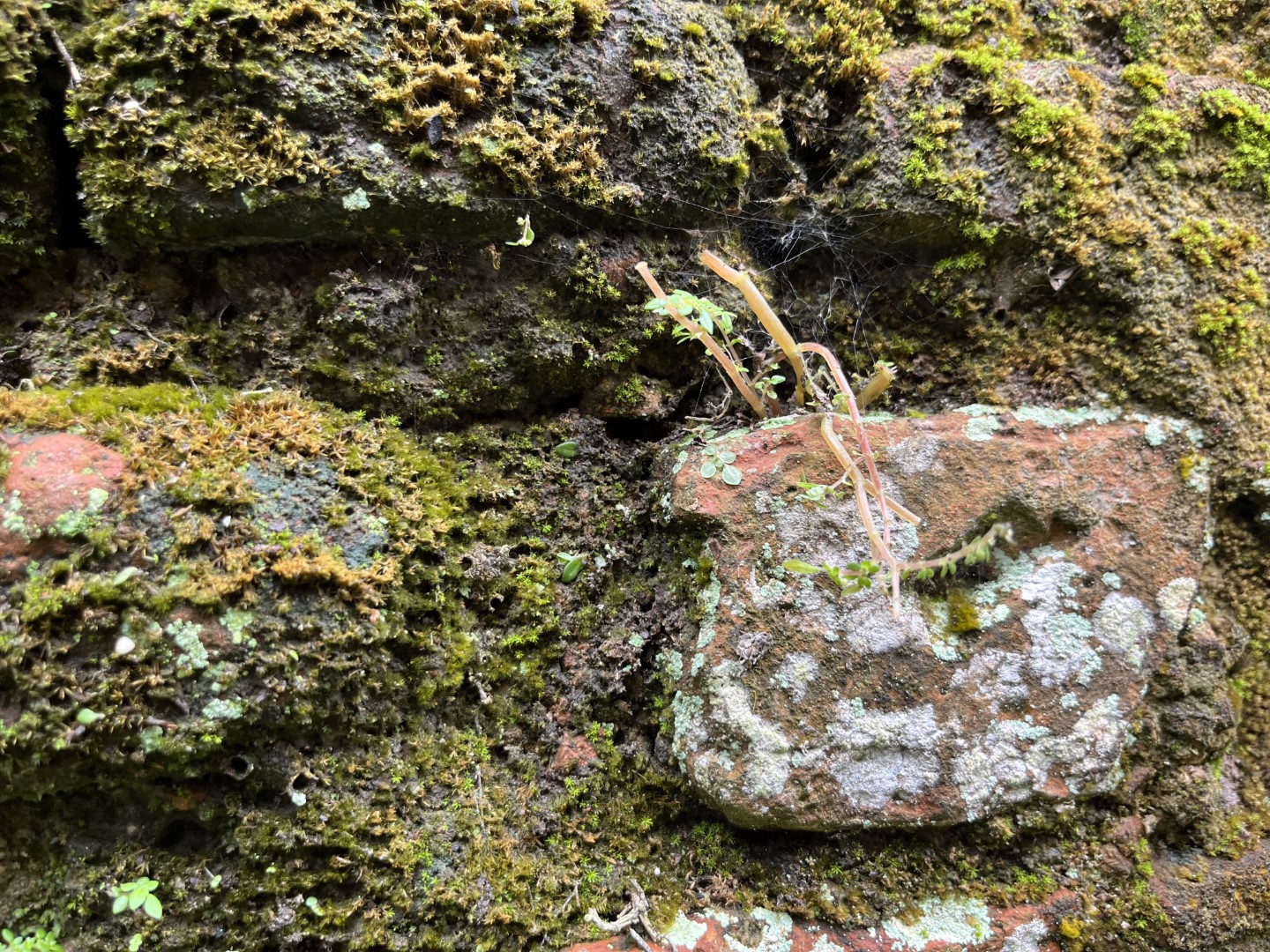

Supplement: Supplementary file 1 [file mmc1.zip › Demo_Historic_Place_Dataset/Living plant/IMG_3629.JPG]

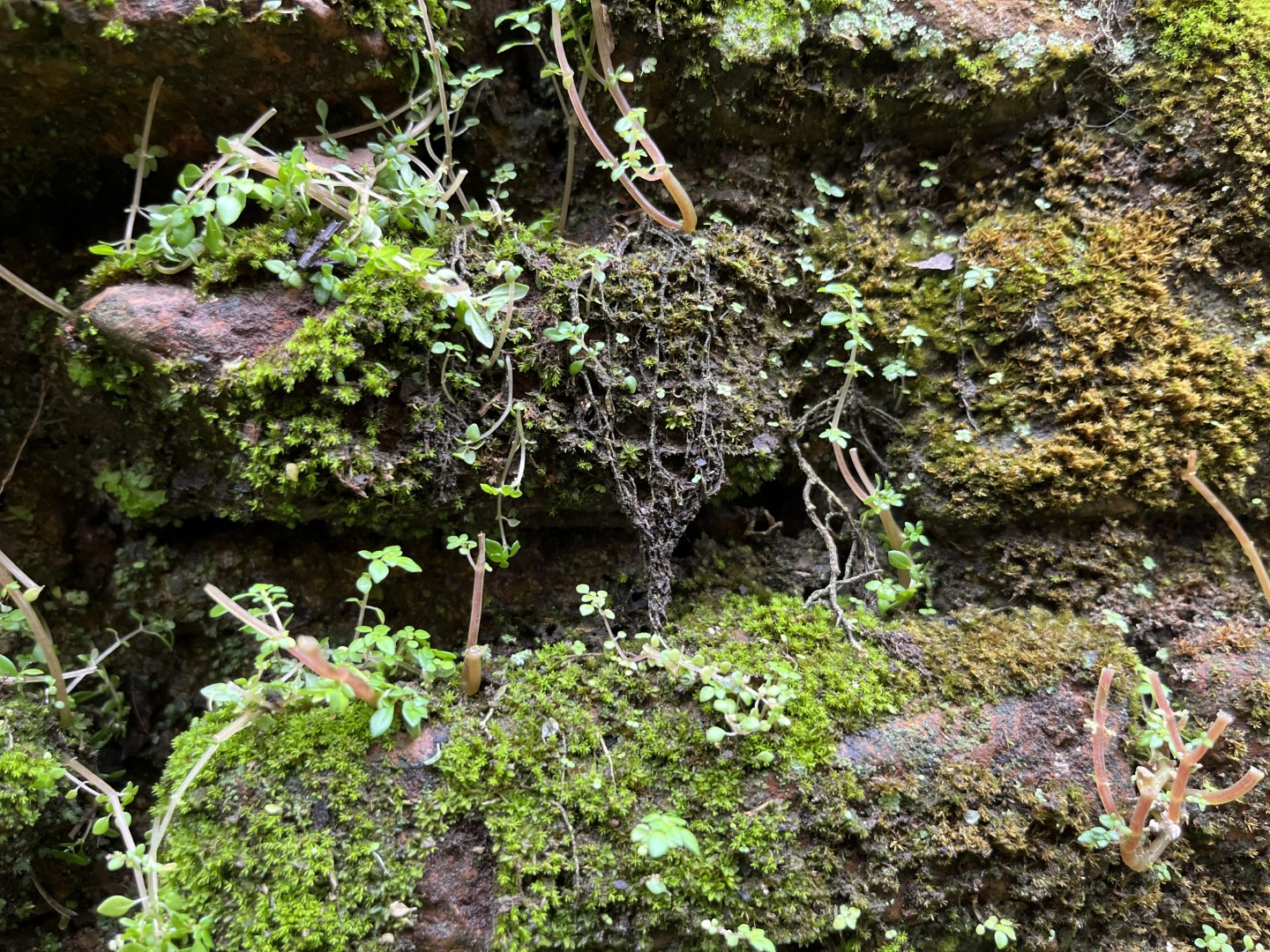

Supplement: Supplementary file 1 [file mmc1.zip › Demo_Historic_Place_Dataset/Living plant/IMG_3630.JPG]

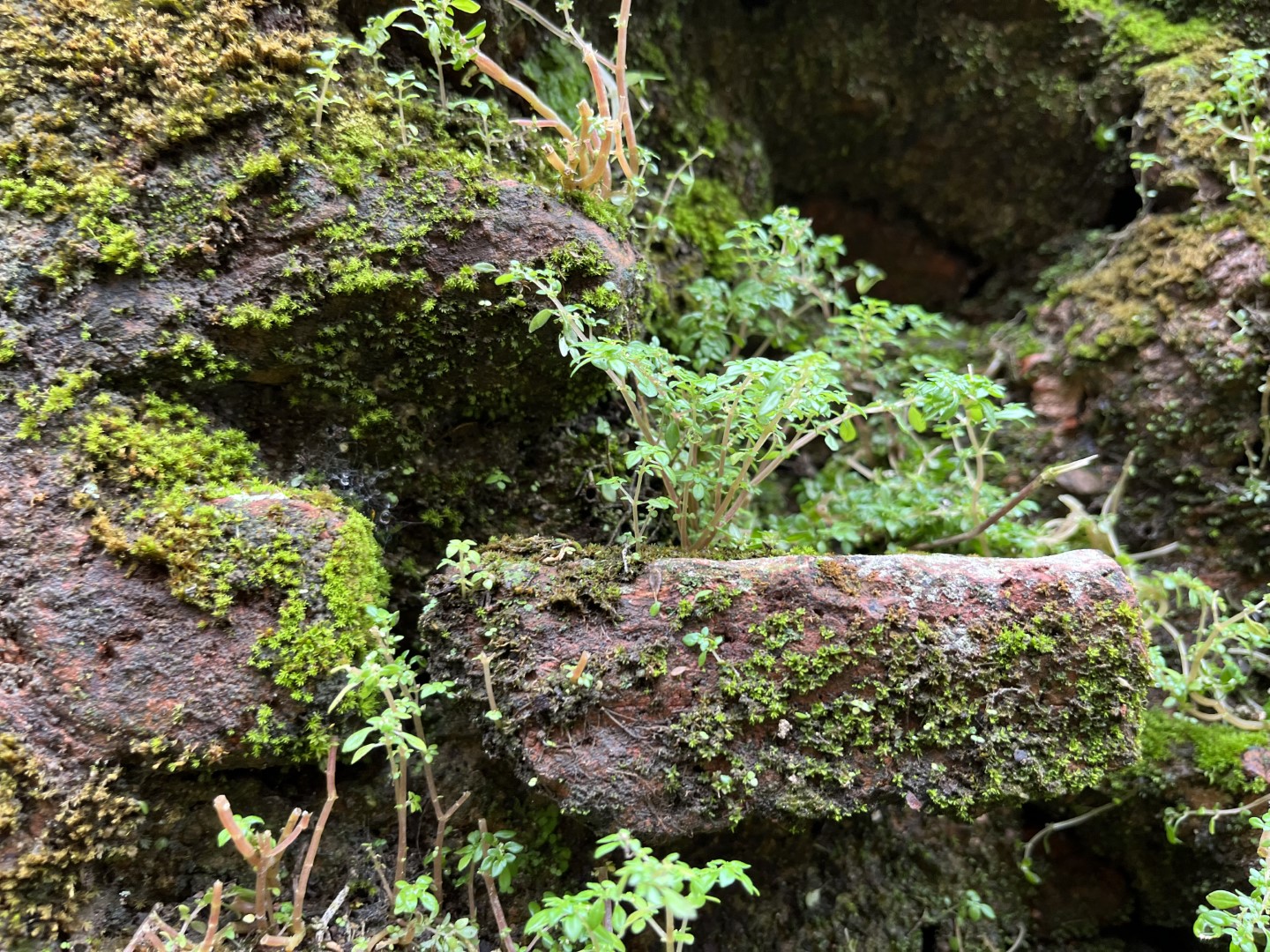

Supplement: Supplementary file 1 [file mmc1.zip › Demo_Historic_Place_Dataset/Living plant/IMG_3631.JPG]
